# School‐based interventions for reducing disciplinary school exclusion: a systematic review

**DOI:** 10.4073/csr.2018.1

**Published:** 2018-01-09

**Authors:** Sara Valdebenito, Manuel Eisner, David P. Farrington, Maria M. Ttofi, Alex Sutherland

## Abstract

This Campbell systematic review examines the impact of interventions to reduce exclusion from school. School exclusion, also known as suspension in some countries, is a disciplinary sanction imposed by a responsible school authority, in reaction to students' misbehaviour. Exclusion entails the removal of pupils from regular teaching for a period during which they are not allowed to be present in the classroom (in‐school) or on school premises (out‐of‐school). In some extreme cases the student is not allowed to come back to the same school (expulsion). The review summarises findings from 37 reports covering nine different types of intervention. Most studies were from the USA, and the remainder from the UK.

Included studies evaluated school‐based interventions or school‐supported interventions to reduce the rates of exclusion. Interventions were implemented in mainstream schools and targeted school‐aged children from four to 18, irrespective of nationality or social background. Only randomised controlled trials are included.

The evidence base covers 37 studies. Thirty‐three studies were from the USA, three from the UK, and for one study the country was not clear.

School‐based interventions cause a small and significant drop in exclusion rates during the first six months after intervention (on average), but this effect is not sustained. Interventions seemed to be more effective at reducing some types of exclusion such as expulsion and in‐school exclusion.

Four intervention types – enhancement of academic skills, counselling, mentoring/monitoring, and skills training for teachers – had significant desirable effects on exclusion. However, the number of studies in each case is low, so this result needs to be treated with caution.

There is no impact of the interventions on antisocial behaviour.

Variations in effect sizes are not explained by participants' characteristics, the theoretical basis of the interventions, or the quality of the intervention. Independent evaluator teams reported lower effect sizes than research teams who were also involved in the design and/or delivery of the intervention.

**Plain language summary:**

**Interventions can reduce school exclusion but the effect is temporary**

Some interventions – enhancement of academic skills, counselling, mentoring/monitoring, and skills training for teachers – appear to have significant effects on exclusion.

**The review in brief**

Interventions to reduce school exclusion are intended to mitigate the adverse effects of this school sanction. Some approaches, namely those involving enhancement of academic skills, counselling, mentoring/monitoring and those targeting skills training for teachers, have a temporary effect in reducing exclusion. More evaluations are needed to identify the most effective types of intervention; and whether similar effects are also found in different countries.

**What is the aim of this review?**

This Campbell systematic review examines the impact of interventions to reduce exclusion from school. School exclusion, also known as suspension in some countries, is a disciplinary sanction imposed by a responsible school authority, in reaction to students’ misbehaviour. Exclusion entails the removal of pupils from regular teaching for a period during which they are not allowed to be present in the classroom (in‐school) or on school premises (out‐of‐school). In some extreme cases the student is not allowed to come back to the same school (expulsion). The review summarises findings from 37 reports covering nine different types of intervention. Most studies were from the USA, and the remainder from the UK.

**What is this review about?**

School exclusion is associated with undesirable effects on developmental outcomes. It increases the likelihood of poor academic performance, antisocial behavior, and poor employment prospects. This school sanction disproportionally affects males, ethnic minorities, those who come from disadvantaged economic backgrounds, and those with special educational needs.

This review assesses the effectiveness of programmes to reduce the prevalence of exclusion.

**What are the main findings of this review?**

*What studies are included?*

Included studies evaluated school‐based interventions or school‐supported interventions to reduce the rates of exclusion. Interventions were implemented in mainstream schools and targeted school‐aged children from four to 18, irrespective of nationality or social background. Only randomised controlled trials are included.

The evidence base covers 37 studies. Thirty‐three studies were from the USA, three from the UK, and for one study the country was not clear.

School‐based interventions cause a small and significant drop in exclusion rates during the first six months after intervention (on average), but this effect is not sustained. Interventions seemed to be more effective at reducing some types of exclusion such as expulsion and in‐school exclusion.

Four intervention types ‐ enhancement of academic skills, counselling, mentoring/ monitoring, and skills training for teachers – had significant desirable effects on exclusion. However, the number of studies in each case is low, so this result needs to be treated with caution.

There is no impact of the interventions on antisocial behaviour.

Variations in effect sizes are not explained by participants’ characteristics, the theoretical basis of the interventions, or the quality of the intervention. Independent evaluator teams reported lower effect sizes than research teams who were also involved in the design and/or delivery of the intervention.

**What do the findings of this review mean?**

School‐based interventions are effective at reducing school exclusion immediately after, and for a few months after, the intervention (6 months on average). Four interventions presented promising and significant results in reducing exclusion, that is, enhancement of academic skills, counselling, mentoring/monitoring, skills training for teachers. However, since the number of studies for each sub‐type of intervention was low, we suggest these results should be treated with caution.

Most of the studies come from the USA. Evaluations are needed from other countries in which exclusion is common. Further research should take advantage of the possibility of conducting cluster‐randomised controlled trials, whilst ensuring that the sample size is sufficiently large.

**How up‐to‐date is this review?**

The review authors searched for studies published up to December 2015. This Campbell systematic review was published in January 2018.

**Executive Summary/Abstract:**

**BACKGROUND**

Schools are important institutions of formal social control (Maimon, Antonaccio, & French, 2012). They are, apart from families, the primary social system in which individuals are socialised to follow specific codes of conduct. Violating these codes of conduct may result in some form of punishment. School punishment is normally accepted by families and students as a consequence of transgression, and in that sense school isoften the place where children are first introduced to discipline, justice, or injustice (Whitford & Levine‐Donnerstein, 2014).

A wide range of punishments may be used in schools, from verbal reprimands to more serious actions such as detention, fixed term exclusion or even permanent exclusion from the mainstream education system. It must be said that in some way, these school sanctions resemble the penal system and its array of alternatives to punish those that break the law.

School exclusion, also known as suspension in some countries, is defined as a disciplinary sanction imposed by a responsible school authority, in reaction to students’ misbehaviour. Exclusion entails the removal of pupils from regular teaching for a period during which they are not allowed to be present in the classroom or, in more serious cases, on school premises.**Based on the previous definition, this review uses school exclusion and school suspension as synonyms, unless the contrary is explicitly stated.**

Most of the available research has found that exclusion correlates with subsequent negative sequels on developmental outcomes. Exclusion or suspension of students is associated with failure within the academic curriculum, aggravated antisocial behaviour, and an increased likelihood of involvement with punitive social control institutions (i.e., the Juvenile Justice System). In the long‐term, opportunities for training and employment seem to be considerably reduced for those who have repeatedly been excluded. In addition to these negative correlated outcomes, previous evidence suggest that the exclusion of students involves a high economic cost for taxpayers and society.

Research from the last 20 years has concluded quite consistently that this disciplinary measure disproportionally targets males, ethnic minorities, those who come from disadvantaged economic backgrounds, and those presenting special educational needs. In other words, suspension affects the most vulnerable children in schools.

Different programmes have attempted to reduce the prevalence of exclusion. Although some of them have shown promising results, so far, no comprehensive systematic review has examined these programmes’ overall effectiveness.

**OBJECTIVES**

The main goal of the present research is to systematically examine the available evidence for the effectiveness of different types of school‐based interventions aimed at reducing disciplinary school exclusion. Secondary goals include comparing different approaches and identifying those that could potentially demonstrate larger and more significant effects.

The research questions underlying this project are as follows:

- Do school‐based programmes reduce the use of exclusionary sanctions in schools?
- Are some school‐based approaches more effective than others in reducing exclusionary sanctions?
- Do participants’ characteristics (e.g., age, gender, ethnicity) affect the impact of school‐based programmes on exclusionary sanctions in schools?
- Do characteristics of the interventions, implementation, and methodology affect the impact of school‐based programmes on exclusionary sanctions in schools?

**SEARCH METHODS**

The authors conducted a comprehensive search to locate relevant studies reporting on the impact of school‐based interventions on exclusion from 1980 onwards. Twenty‐seven different databases were consulted, including databases that contained both published and unpublished literature. In addition, we contacted researchers in the field of school‐exclusion for further recommendations of relevant studies; we also assessed citation lists from previous systematic and narrative reviews and research reports. Searches were conducted from September 1 to December 1, 2015.

**SELECTION CRITERIA**

The inclusion and exclusion criteria for manuscripts were defined before we started our searches. To be eligible, studies needed to have: evaluated school‐based interventions or school‐supported interventions intended to reduce the rates of suspension; seen the interventions as an alternative to exclusion; targeted school‐aged children from four to 18 in mainstream schools irrespective of nationality or social background; and reported results of interventions delivered from 1980 onwards. In terms of methodological design, we included randomised controlled trialsonly, with at least one experimental group and onecontrol or placebo group.

**DATA COLLECTION AND ANALYSIS**

Initial searches produced a total of 42,749 references from 27 different electronic databases. After screening the title, abstract and key words, we kept 1,474 relevant hits. 22 additional manuscripts were identified through other sources (e.g., assessment of citation lists, contribution of authors). After removing duplicates, we ended up with a total of 517 manuscripts. Two independent coders evaluated each report, to determine inclusion or exclusion.

The second round of evaluation excluded 472 papers, with eight papers awaiting classification, and 37 studies kept for inclusion in meta‐analysis. Two independent evaluators assessed all the included manuscripts for risk of quality bias by using EPOC tool.

Due to the broad scope of our targeted programmes, meta‐analysis was conducted under a random‐effect model. We report the impact of the intervention using standardised differences of means, 95% confidence intervals along with the respective forest plots. Sub‐group analysis and meta‐regression were used for examining the impact of the programme. Funnel plots and Duval and Tweedie's trim‐and‐fill analysis were used to explore the effect of publication bias.

**RESULTS**

Based on our findings, interventions settled in school can produce a small and significant drop in exclusion rates (SMD=.30; *95% CI* .20 to .41; *p*<.001). This means that those participating in interventions are less likely to be suspended than those allocated to control/placebo groups. These results are based on measures of impact collected immediately during the first six months after treatment (on average). When the impact was tested in the long‐term (i.e., 12 or more months after treatment), the effects of the interventions were not sustained. In fact, there was a substantive reduction in the impact of school‐based programmes (SMD=.15; 95%CI ‐.06 to .35), and it was no longer statistically significant.

We ran analysis testing the impact of school‐based interventions on different types of exclusion. Evidence suggests that interventions are more effective at reducing expulsion and in‐school exclusion than out‐of‐school exclusion. In fact, the impact of intervention in out‐of‐school exclusion was close to zero and not statistically significant.

Nine different types of school‐based interventions were identified across the 37 studies included in the review. Four of them presented favourable and significant results in reducing exclusion (i.e., enhancement of academic skills, counselling, mentoring/monitoring, skills training for teachers). Since the number of studies for each sub‐type of intervention was low, we suggest that results should be treated with caution.

A priori defined moderators (i.e., participants’ characteristics, the theoretical basis of the interventions, and quality of the intervention)showed not to be effective at explaining the heterogeneity present in our results. Among three post‐hoc moderators, the role of the evaluator was found to be significant: independent evaluator teams reported lower effect sizes than research teams who were also involved in the design and/or delivery of the intervention.

Two researchers independently evaluated the quality of the evidence involved in this review by using the EPOC tool. Most of the studies did not present enough information for the judgement of quality bias.

**AUTHORS’ CONCLUSIONS**

The evidence suggests that school‐based interventions are effective at reducing school exclusion immediately after, and for a few months after, the intervention. Some specific types of interventions show more promising and stable results than others, namely those involving mentoring/monitoring and those targeting skills training for teachers. However, based on the number of studies involved in our calculations, we suggest that results must be cautiously interpreted. Implications for policy and practice arising from our results are discussed.

## 1. Background

### 1.1 THE PROBLEM, CONDITION OR ISSUE

#### 1.1.1 School discipline

Discipline problems are frequent in schools and they may have a harmful effect on pupils’ learning outcomes. A lack of discipline and the subsequent potential increase in school disorder (e.g. bullying, substance misuse) can seriously threaten the quality of instruction that teachers provide, hamper pupils acquisition of academic skills and subsequently reduce their attachment to the education system (Gottfredson, Cook, & Na, 2012).

As such, discipline represents a serious concern for parents and teachers, demanding significant efforts and resources from schools (Kaplan, Gheen, & Midgley, 2002). The PISA 2009 report (OECD, 2010) stated that schools registering higher levels of disciplinary problems result in teachers spending less time on learning in order to deal with such issues. In its 2012 version, the PISA report asked students about school discipline. Results found that “28% of students reported that teachers had to wait a long time to quiet down every class, or almost all classes”(OECD, 2013). Being more precise, the Teaching and Learning International Survey (TALIS) revealed that teachers spend at least 20% of lesson time dealing with disruption and administrative tasks. In the United Kingdom, the Behaviour Survey 2010 states that 80% of school teachers felt their ability to teach effectively was impaired by students’ poor behaviour (Massey, 2011). On a global level, evidence suggests that 13% of teachers’ time is, on average, spent maintaining order (OECD, 2009).

Schools use different procedures to manage discipline, including a range of punitive responses (e.g., loss of privileges, additional homework or detention). Among these, exclusion is normally seen as one of the most serious punishments. Although types and lengths vary from country to country, school exclusion (also known as school suspension in the United States)^1^ can be broadly defined as a disciplinary sanction imposed in reaction to students’ behaviour (i.e. violations of school policies) by the responsible authority. In concrete terms, exclusion entails a removal from regular teaching for a period of time during which students are also not allowed to be present on school premises. Specifically, fixed‐term exclusions consist of a limited number of hours or days (Cornell, Gregory, & Fan, 2011), whereas permanent exclusion (i.e., expulsion) involves the pupil being transferred to a different school, or educated outside of the regular education system (Spink, 2011; Webb & Vulliamy, 2004).

Even if school policies suggest that exclusion should be used as a last resort, reserved for only the most serious and persistent offences (Gregory & Weinstein, 2008a; Skiba & Peterson, 1999; Skiba, Trachok, Chung, Baker, & Hughes, 2012), research evidence suggests that minor offences can also provoke this type of punishment (Munn, Cullen, Johnstone, & Lloyd, 2001; Skiba, 2014). Fenning et al., (2012) provide a case in point: their research concluded that suspension and expulsion were the most common types of punishment for minor problems such astardiness and school truancy. These findings were also confirmed by Liu (2013) who found that 48% of suspensions lasting a maximum of five days targeted minor disorder or disruptive behaviours.

In terms of prevalence, data provided by the UK Department for Education (academic year 2011/12) shows that in England fixed‐term exclusion affects 3.5% of the school population whereas permanent exclusion applies to only 0.06%. The national figures suggest that students in secondary‐level education (6.8% of the school population) as well as those in special education (14.7%) are the most likely to experience fixed‐term exclusion (DfE, 2013). In the United States, data provided by the Department of Education (academic year 2011/12) concluded that 7.4% (3.5 million) of students were suspended in school, 7% (3.45 million) were suspended out‐of‐school, and less than one per cent were subject to expulsion (around 130,000 students). Black students and those presenting disabilities are, respectively, three and two times more likely to be excluded compared to White and non‐disabled pupils (U.S. Department of Education, 2012).

International comparisons of exclusion prevalence rates are not available in the literature examined. Indeed, differences in use, extent and recording (i.e., unreported exclusions) make an international estimation challenging. In Table 1, the reader will find information regarding the use of exclusion in a sample of high‐ and middle‐income countries. The information is limited to a convenience sample involving twelve different cases to allow an overview of i) the types of exclusion used in these countries, ii) the length of the sanction, iii) the authority responsible for determining this sanction, iv) the behaviours for which school exclusion is permissible, and, in cases where information was available, the table also includes v) the local prevalence of exclusion. This does not claim to be a representative sample of all countries, but as an initial approach will help provide a more complex picture of the phenomenon. In addition, this comparison was intended to help with searches for studies that could be potentially included in the systematic review. For instance, by comparing exclusion in different countries, it was found that the same school sanction had different names in different countries (e.g., “stand‐down” in New Zealand, “exclusion” in the UK and “suspension” in the US).

**Table 1:** Comparative description of school exclusion in a sample of high‐ and middle‐income countries

**Country**	**Name given**	**Type of exclusions**	**Length (for fixed exclusions)**	**Who makes the decision?**	**Legal reasons for exclusion**	**Prevalence** ^2^
**Australia** ^3^ **(New South Wales)**	Suspension	Short suspensions	4 school days.	School Principal	Continued disobedience and aggressive behaviour	Unknown
		Long suspensions	Up to 20 school days.	School Principal	Physical violence, use or possession of prohibited weapons, firearms or knife, possession, use or supply of a suspected illegal substance, serious criminal behaviour, use a weapon, persistent or serious misbehaviour	
	Expulsion	Permanent	Permanent	School Principal	In serious circumstances of misbehaviour, the principal may expel a student of any age from their school. The principal may also expel a student who is over 17 years of age for unsatisfactory participation in learning.	
**Canada** ^4^ **(Ontario)**	Suspension	Short‐term Long‐term	1 to 20 school days. More than five school days are considered long‐term.	School Principal. Parents must be informed within 24 hours. All suspensions can be appealed to the school board.	Threat to inflict serious bodily harm on another person, possessing alcohol or illegal drugs, being under the influence of alcohol, swearing at a teacher or at another person in a position of authority, committing an act of vandalism that causes extensive damage to school property, or bullying.	2.76% of enrolled students (N= 2,014,407). Academic Year 2013‐2014^5^
	Expulsion	From school From all schools (in this case, the students must be offered alternative education)	Indefinite.	School Principal should recommend expulsion to the school board. Parents must be informed within 24 hours. All expulsions can be appealed at a tribunal.	Possessing or using a weapon, physical assault on another person that causes bodily harm requiring treatment by a medical practitioner, sexual assault, trafficking in weapons, trafficking in illegal drugs, robbery, drinking alcohol.	0.02% of enrolled students (N= 2,014,407) Academic Year 2013‐2014^6^
**Chile** ^7^	Suspension	Fixed. Implemented inside the school premises	The law does not limit the duration of fixed suspensions. Each school community issues their own disciplinary code and defines disciplinary sanctions and their duration.	Disciplinary Board	Defined for each school, but it must be used in exceptional cases	Unknown
	Expulsion			School Principal	Conduct that directly threatens the physical or psychological integrity of any member of the school community^8^	Unknown
**Colombia** ^9^	Suspension	Fixed	Each school community issues their disciplinary codes and defines disciplinary sanctions and their duration. Normally fixed exclusion lasts 3 days.	Discretionary	Violation to the code of conduct	Unknown
		Definitive			Unknown	
**Costa Rica** ^10^	Suspension	Fixed	Up to 8 school days.	School Principal	Not clearly stated	Unknown
		Permanent		School Board	Permanent disruptive/defiant behaviour, non‐compliance with previous sanctions, violence and aggressions towards a member of the school community, lack of moral integrity.	
**England** ^11^	Exclusion	Fixed (in‐school, out‐of‐school)	1‐45 days per year. After 5 days of fixed out‐of‐school exclusion, the school must provide alternative education.	Discretionary School principal	Repeated failure to follow academic instruction, failure to complete a behavioural sanction (e.g. with a detention, a decision to change the sanction to exclusion would not automatically be unlawful), repeated and persistent breaches of the schools’ behavioural policy.	Academic year 2014‐2015^12^ 3.8% of students (all schools) 7.51% of students (secondary schools)
		Permanent				0.07% of students (all schools) 0.15% of students (secondary schools)
**France** ^13^	Exclusion	Temporary exclusion from the classroom	Maximum of 8 days.	Consultation between the various members of the pedagogical and educational team	Serious cases of violence (physical or psychological) against the school community	Unknown
		Temporary exclusion from school	Maximum of 8 days.	School principal or school board		
		Definitive exclusion	Permanent	Disciplinary board. The student should be represented on the disciplinary board.		
**Finland** ^14^	Exclusion	In‐school exclusion and out‐of‐school exclusion with the school obligated to provide education at home.	In‐school exclusion: remainder of the day. Out‐of‐school exclusion: no more than 3 months. It is a very infrequent measure.	Teacher and school principal using a formal procedure. In cases of out‐of‐school exclusion, a personal plan of education must be provided and local social services should be informed.	Threats or serious violence that would endanger the safety of other members of the school community	Unknown
		Permanent exclusion does not exist in the local law.				
**Malta**	Suspension	Fixed term suspension	Suspension for the rest of the day or for a few days. The number of days is not stated in the law.	Must be applied by the Head of School after the student's parent or guardian has been informed. The National Board for School Behaviour should be consulted.	The law defines 3 levels of misbehaviour. Suspension and expulsion are restricted for level 3, meaning serious offenses only. No further details.	Unknown
	Expulsion	Expulsion	Permanent			
**Norway** ^15^	Exclusion	Fixed exclusion, expulsion for the rest of the year and loss of rights to education.	Primary education (level 1‐7): exclusion from specific lessons or for the rest of the day. Secondary education (level 8‐10): maximum of 3 days. Expulsion and loss of rights are defined in the Educational Law but its use is extremely rare.	The school principal in consultation with the pupil's teacher, unless the local authority defines a different procedure.	Exclusion is used as a last resort and can be justified only for serious issues of violence. The law suggests the use of alternatives such as mediation before imposing an exclusion.	Unknown
**New Zealand** ^16^	Stand‐down	Stand‐down	The student is removed from school for 5 school days in a term or 10 school days in a year.	School Principal, through a formal procedure that includes informing the family, the Education Authority and the school board.	Drugs (including substance abuse), continual disobedience and physical assault on other students were the most prevalent causes for stand‐down, suspensions, exclusion and expulsion.	1.5% of school population (2015)
	Suspension	Suspension	The student is removed from school for no more than 7 days.	School Board		0.3% of school population (2015)
	Exclusion	Expulsion	Maximum of 10 days in a year.	School Board		0.1% of the total student population under 16 years old (2015)
	Exclusion	Expulsion	A student under the age of 16 would be excluded from the school, with the requirement that the student enrolls elsewhere	School Board		0.2% of the total student population over 16 years old (2015) ^17^
			A student aged 16 or over would be expelled from the school, and the student may or may not enroll at another school.			
**The US, Washington DC** ^18^	Suspension	Suspension (short‐term and long‐term) is a restriction in attending school or school activities.	Short‐term suspension: maximum of 10 consecutive days. Long‐term suspension: more than 10 consecutive days.	Certified teachers can decide a suspension but it must be communicated to the school principal. Short‐term suspensions must be formally communicated to the student/parents. Long‐term suspensions and expulsions require a formal process (i.e., written notice by the school district) and should be known by the School Principal.	Violation of school district rules	3.89% of all Washington students have been suspended or expelled (2014–15) The rate of suspensions and expulsions across districts range between nearly 0% to over 10% of students^19^.
	Expulsion	Expulsion makes this restriction indefinite.	Maximum: 1 calendar year		Violation of school district rules, serious violence, gang activity on school grounds.	
		Emergency expulsion	Temporary. The student would go back once the danger ceases		The student's presence poses an immediate and continuing danger to others. The student's presence poses a threat of substantial disruption in the classroom.	
**The US, Virginia** ^20^	Removal from classes	In‐school		Teacher	Disruptive behaviour	Unknown
	Suspension	Suspension (short‐term and long‐term) is a restriction in attending school or school activities.	Short‐term suspension: 10 consecutive or 10 cumulative school days in a school year Long‐term suspension: more than 10 school days but less than 365 calendar days.	Imposed by the school principal, any assistant principal or, in their absence, any teacher. The suspension should entail a formal process. The student must be heard.	Violation of school code of conduct	
	Expulsion	Expulsion makes the restriction last longer.	A student is not permitted to attend school within the school division and is ineligible for readmission for 365 calendar days after expulsion.	Imposed by a committee from the school board. Includes a formal process, written notice and appeal.	Criminal activity, carrying a weapon, drug related offences, or when the pupil presence is a clear threat for the school community.	
**The US, Texas** ^21^	Suspension	In‐school suspension (e.g., seclusion units)	In‐school suspension lasts between 1 class and several days.	Low‐level offences are dealt with on a discretionary basis (according to a defined code of conduct) by the designated administrator (usually the principal or vice principal). Higher‐level offences require mandatory removal from the classroom. Rules for a due process are defined.	Violation of school code of conduct (unruly, disruptive, or abusive behaviours)	9.24% (2014‐2015)^22^
	Expulsion	Out‐of‐school suspension	Out‐of‐school suspension should be no longer than 3 days.	Unknown	Weapon carrying, serious violence or crimes.	4.33% (2014‐2015)
		In the case of serious offences, a student can be expelled from school.	At least 1 year Disciplinary Alternative Education Program (DAEP) for students removed for over 3 days (no maximum period provided).	Unknown		3.39% (2014‐2015)

2 Prevalence was calculated by dividing the number of excluded students per year (numerator) by the total number of students per year (denominator).

3 Information retrieved from “Suspension and Expulsion of School Students” New South Wales Government. Updated in October 2014 https://www.det.nsw.edu.au/policies/student_serv/discipline/stu_discip_gov/suspol_07.pdf

4 http://www.edu.gov.on.ca/eng/safeschools/NeedtoKnowSExp.pdf

5 http://www.edu.gov.on.ca/eng/safeschools/statistics.html

6 http://www.edu.gov.on.ca/eng/safeschools/statistics.html

7 http://www.supereduc.cl/. Additionally, the information can be found in Torche & Mizala (2012)

8 http://denuncias.supereduc.cl/cuestionario1/denuncias_tematicos.html

9 In Colombia, each school must define school exclusion length. This is established in the Ley General de Educación N° 115, February 1994. http://www.mineducacion.gov.co/1621/articles‐85906_archivo_pdf.pdf. Additional information can be retrieved from http://www.mineducacion.gov.co/1621/article‐86906.html

10 http://www.mep.go.cr/sites/default/files/Reglamento_General_Establecimientos_Oficiales_de_Educacion_Media.pdf

11 In England, exclusions are regulated by the Education Act of 2002

12 https://www.gov.uk/government/uploads/system/uploads/attachment_data/file/539704/SFR_26_2016_text.pdf

13 In France, school exclusions are regulated by the Code de l'education: http://www.education.gouv.fr/cid56670/sanctions‐scolaires‐reforme‐des‐procedures‐disciplinaires‐dans‐les‐etablissements‐scolaires.html

14 Basic Education Act 628/1998 (Amendments up to 1136/2010). http://www.finlex.fi/en/laki/kaannokset/1998/en19980628.pdf

15 LOV 1998‐07‐17 nr 61: Law on Primary and Secondary Education (The Education Act)

16 In New Zealand, the guidance for suspensions is based on the Education Act of 1989 and the Education Rules 1999 (Stand‐down, Suspension, Exclusion, and Expulsion)

17 All data referring to prevalence was extracted from a governmental report informing data from academic years 2015. http://www.educationcounts.govt.nz/__data/assets/pdf_file/0011/147764/SSEE‐Indicator‐Report‐2015‐Data.pdf

18 In the US, procedures and definitions of school suspension vary among states. Here, we use Washington State as an example. For more details, see www.k12.wa.us/Safetycenter/Discipline/pubdocs/Suspension‐expulsion‐rights.pdf

19 Data extracted from Office of Super Intendent of Education (OSIP), State of Washington. http://www.k12.wa.us/DataAdmin/PerformanceIndicators/DataAnalytics.aspx#discipline

20 See the specific section for Virginia, p. 10‐16 in https://safesupportivelearning.ed.gov/sites/default/files/disciplinecompendium/School%20Discipline%20Laws%20and%20Regulations%20Compendium.pdf

21 See the specific section for Texas, p. 14–27 in https://safesupportivelearning.ed.gov/sites/default/files/discipline‐compendium/School%20Discipline%20Laws%20and%20Regulations%20Compendium.pdf

22 Data extracted from the Texas Education Agency based on categories which count students once. https://rptsvr1.tea.texas.gov/cgi/sas/broker

The comparative data incorporated in the table above suggests heterogeneity in the application of exclusion. For instance, in the US, Norway and England, educational systems distinguish between fixed and permanent exclusion. However, in some educational systems, such as Finland's, the law only permits fixed‐term exclusion. Concerning length, England limits fixed‐term exclusions to a maximum of 45 days per school year while New Zealand's legislation allows exclusions for a maximum of 10 days per year. On the other hand, it is important to note that in some countries – such as France – specific laws define and regulate exclusion, whereas in others – like Chile and Colombia – the ability to set the length of the sanction is granted to each school.

Although the data on prevalence is limited to a few countries, the percentage of in‐school exclusion seems to be larger than out‐of‐school exclusion and expulsion. In New Zealand, the data suggests that the use of exclusion is marginal particularly when compared with some areas in the US and the UK.

### 1.2 PREDICTORS AND OUTCOMES

The research concerning predictors and outcomes of school exclusion has some limitations it is necessary to address before arriving at any final conclusions. Regarding predictors, only ethnicity seems to have a clear role in predicting exclusion. For other variables of interest such as sex, age or socio‐economic status most of the evidence is limited to bivariate associations.

Regarding the outcomes, while there is a stark link between misbehaviour (e.g., school drop‐out and delinquency) and school exclusion, there is no clear causal relationship. Notwithstanding decades of research on school exclusion and its impact on later behaviour, we are still at an initial stage for testing causal associations in these matters. The association between exclusion and these negative outcomes may simply reflect underlying behavioural tendencies that lead to conduct problems, exclusion and poor outcomes later in life – that is, the antisocial syndrome described by Farrington (1997). In fact, school exclusion and the behaviours outlined here as “negatives” could be explained by the personality traits of the syndrome.

As stated by Sutherland and Eisner (2014) “it is currently unclear whether the disciplinary action itself has a causal effect over and beyond the social, familial and behavioural characteristics of the affected children. To date, studies have used analytical approaches that are unable to reliably establish a robust link between exclusion and outcomes such as criminal behavior.” Some longitudinal studies have attempted to deal with this matter by controlling for previous behavioural characteristics that could alter the impact of the sanction. When this is the case, the methodological details are explicitly presented in this review.

Keeping these reservations in mind, the following section describes variables associated with the prediction of school exclusion, as well as some negative outcomes linked to exclusion.

#### 1.2.1 Predictors of school exclusion

From a normative point of view, school exclusion is a punitive response for misbehaviour. In that sense, behavioural problems seem to be the most obvious empirical predictor for exclusion. Reinke, Herman, Petras, & Ialongo, (2008) illustrate the role of problem behaviour in exclusion by conducting a latent class analysis. Participants in the subclass of boys exhibiting behavioural problems only (i.e., isolating other academic/learning difficulties) were almost 4 times more likely to be suspended (*OR* = 3.42; *95%CI* 1.36 to 8.58; *p* < .05) than their non‐problematic peers. Similarly, Pas, Bradshaw, Hershfeldt, & Leaf, (2010) found that after controlling for student, teacher, classroom, and school level covariates, the strongest predictor for out‐of‐school suspension was disruptive behaviour (*OR* = 4.83; *95%CI* 4.10 to 5.68; *p* < .05).

Despite the role of behaviour in school exclusion, research suggests that it is not the sole or even the most prominent predictor. In fact, previous findings show a more complex scenario where exclusion is also strongly predicted by gender, ethnicity, age, economic background, and special educational needs (Costenbader & Markson, 1998a; Mcloughlin & Noltemeyer, 2010; Monroe, 2005; Nickerson & Spears, 2007; Noltemeyer & Mcloughlin, 2010; Skiba et al., 2011; Yudof, 1975). In the following paragraphs, we offer an overview of the role of these variables in predicting school exclusion.

##### Gender as a predictor of exclusion

Data provided by the Department for Education in England (DfE) 2011/12 suggests that male pupils are around three times more likely to be punished by exclusion than female pupils (DfE, 2013). The same trend can be observed in the study published by Liu (2013) based on longitudinal data from 13,875 American students. The study reports the predominance of males being excluded, but recognises that the proportion of females excluded tends to increase from elementary (23.7%), to secondary (32.7%), to high school (35.2%). More specifically, Bowman‐Perrott et al. (2013, p. 91) concluded that, based on a sample of 2,597 pupils, the predominance of males in exclusion rates (OR = 2.28) was even larger in the case of pupils with learning disabilities (OR = 4.31).^2^

##### Ethnicity

Research outcomes suggest a clear and consistent disproportionality in the prevalence of ethnic minorities as a target for disciplinary exclusion (Anyon et al., 2014; Gregory, Skiba, & Noguera, 2010). In the US, different sources of data show that school exclusion overly affects minorities such as Afro‐Caribbean (Noltemeyer & Mcloughlin, 2010), Latino (Skiba et al., 2011) and American Indian students (Gregory et al., 2010) in comparison with their White peers. In the UK, data from the (DfE, 2012) showed that: “The rate of exclusions was highest for Travellers of Irish Heritage, Black Caribbean and Gypsy/Roman ethnic groups. Black Caribbean pupils were nearly 4 times more likely to receive a permanent exclusion than the school population as a whole and were twice as likely to receive a fixed period exclusion.” Notably, recent multivariate analysis points out that racial disproportionality in exclusion still remains significant after controlling by behaviour, number and type of school offences, age, gender, teacher's ethnicity, and socio‐economic status (Fabelo et al., 2011; Noltemeyer & Mcloughlin, 2010; Rocque & Paternoster, 2011; Skiba, Michael, Nardo, & Peterson, 2002). Consider, for instance, a substantial longitudinal report produced by Fabelo et al., (2011) in Texas (N=928,940), intended to isolate the effect of race alone on disciplinary actions. The study used a multivariate analysis controlling for 83 different variables. The findings suggest that African‐American students were 31% more likely to be removed from classrooms compared to White and Hispanic students. In the same vein, Skiba (2015) has argued that, in the United States at least, racial disproportionality in school discipline is ubiquitous. In his opinion, ethnic minorities are overrepresented in almost all types of school punishment. Even more worryingly, instances of exclusionary discipline among African Americans have continued to increase over the years.

Possible reasons for this overrepresentation of Black students, even when controlling for demographic and risk factors, have been addresses by some scholars, who suggest that a racist bias could explain the phenomenon (Losen, 2011; Skiba et al., 2002; Skiba, 2015). In particular, Simson (2014) asserts that racial stereotyping (conscious or unconscious) as well as a cultural mismatch between teachers and students can explain at least some part of the existing racial disproportionality in school discipline.

It is important to say that, as stated by Theriot, Craun, & Dupper (2010, p.14), “the over‐representation of ethnic minority students, especially African American students, in school suspension and expulsion is one of the most consistent—and perhaps most controversial—findings in the extant literature on school discipline.” In general, studies using solid and strong multivariate models highlight the discrimination against racial minorities compared to White students.

##### Age as a predictor of exclusion

The likelihood of being punished by exclusion increases with age, being more frequent during adolescence. In England, 52% of permanent exclusions are imposed on pupils aged between 13 and 14 (DfE, 2013). In the case of American students, the results follow a similar trend. In fact, data reported by Liu, (2013) pointed out that suspensions reach a peak in ninth grade (i.e., 14 to 15 years of age). Also based on a sample of American students, Raush & Skiba, (2004) concluded that the number of out‐of‐school suspensions was significantly higher in secondary schools compared to elementary schools.

##### Socio‐economic status (SES)

Low SES has also been identified as a predictor of high rates of disciplinary exclusion. The UK Department of Education (DfE, 2012) compared the rates of exclusion by eligibility for free school meals (FSM). Those eligible for FSM were 4 times more likely to be punished by a permanent exclusion and around 3 times more likely to get a fixed‐period exclusion than children who were not eligible. In the US, Nichols, (2004) using a sample of 52 schools (37,000 students), found a similar pattern – but the correlation between FSM and exclusion was higher and more significant for pupils in middle school (*r* = .84; *p* <. 01) than for elementary (*r* = ‐.12) or high school pupils (*r* = .48). In Australia, Hemphill et al., (2010), using multilevel mixed‐effects logistic regression (*N* = 8,028 students), concluded that pupils settled in low SES neighbourhoods were exposed to higher rates of exclusion (8.7%) when compared with pupils in high SES areas (2.9%).

However, the evidence still seems to be inconclusive in this respect. Recently, Skiba et al., (2012), using a multilevel approach, tested data from 365 schools and a total number of 43,320 students. They concluded that when comparing those students eligible for free or reduced‐cost lunches with their non‐eligible peers, the first were more likely to get out‐of‐school exclusions (OR= 1.27; p<.05). However, contrary to expectations, the eligibility for free or reduced meals resulted in a negative predictor of permanent exclusion (OR= 0.03; p<.05).

##### Special educational needs (SEN)

Although an increasing amount of research has focused on predictors of school exclusion, analysis of the role of SEN still seems to be limited. In 2007, Achilles, McLaughlin, and Croninger differentiated the role of three different SEN, namely emotional/behavioural disorders (EBD), attention‐deficit/hyperactivity disorders (ADHD), and learning disabilities (LD). Higher rates of exclusion were more likely among those with EBD (OR = 1.49; p<. 001) compared with ADHD (OR = 2.58; p < .001) or LD (OR = 5.44; p < .001). Recently, Bowman‐Perrott et al. (2013), using three waves from the Special Education Elementary Longitudinal Study (SEELS), confirmed that children with emotional or behavioural disorders (OR = 3.95; p <.05) and attention‐deficit or hyperactivity disorders (OR = 4.96; p <.05) were more likely to get suspended or expelled from school than children with learning disabilities (OR = 2.54; p <.05). In a study involving 2,750 students and 39 American schools, Sullivan, Van Norman, & Klingbeil (2014) also observed differences between types of disabilities: those presenting an EBD were at a far greater risk of exclusion (OR = 6.78; SE=0.21) than those presenting other health impairments (i.e., a specific learning disability, intellectual disability, speech and language impairment). When controlling for race and gender, and parents’ education, this trend remained stable and significant. It is important to emphasize that the associations between this disability and exclusion mainly reflect differences in behaviour, respectively psychological or chronic behavioural problems.

#### 1.2.2 Negative outcomes linked to school exclusion

Supporters of zero tolerance policies have pointed out that the use of exclusion can persuade students to account for their behaviour and lead to a decrease in rule‐breaking (Bear, 2012). However, most of the research has consistently documented the negative impact of these types of sanctions (APA Zero Tolerance Task Force, 2008; Chin, Dowdy, Jimerson, & Rime, 2012; Hemphill, Toumbourou, Herrenkohl, McMorris, & Catalano, 2006; Sharkey &Fenning, 2012). In particular, previous research suggests that school exclusion is related to serious negative outcomes in at least three dimensions of young people's development: behavioural, academic, and future social inclusion.

##### Behaviour

Some literature related to the relationship between exclusionary punishments and behaviour suggests that such harsh punishments could result in a spiral into more defiant behaviour by students. Raffaele‐Mendez, (2003), for instance, found a moderate and significant correlation (*r* = .39) between out‐of‐school exclusion (grades 4 to 5) and subsequent exclusion (grade 6). Similarly, Theriot, Craun, & Dupper, (2010) found that pupils punished by in‐school and out‐of‐school exclusion were slightly more likely to get the same punishment again (*OR_in‐school_* = 1.25; *p* < .001; *N* = 9706 and *OR_out‐of‐school_* = 1.32; *p* < .001; *N* = 9706).

Using longitudinal data, Arcia, (2006:366) concluded that school dropout was another behavioural consequence of exclusion. In fact, “43% of students who were suspended 21 or more days dropped out 3 years after their ninth‐grade enrolment.” Similarly, Cratty (2012:649) found a positive correlation between out‐of‐school suspensions and dropout rates. In particular, “those who had an early record of multiple exclusions registered 60% dropout during high school” when compared with non‐excluded students.

The use of exclusion, in turn, is linked with more serious behavioural outcomes such as antisocial conduct, delinquency and entry into the juvenile justice system. Longitudinal research carried out by Hemphill et al. (2006:736) argues that “school suspensions significantly increased antisocial behaviour 12 months later, after holding constant established risk and protective factors (*OR* = 1.5; *95%CI* 1.1 to 2.1; *p*< .05; *N* = 3655)”In terms of the involvement of school excludees in the criminal justice system, Costenbader & Markson, (1998) found significant differences between excluded students and those never excluded. In their view, “while 6% of the students who had never been suspended reported having been arrested, on probation, or on parole, 32% of the externally suspended subsample and 14% of the internally suspended subsample responded positively to this question. Males reported significantly more involvement with the legal system than did females.” (p.67). Meanwhile, Challen & Walton, (2004), studying a population of males in the criminal justice system, concluded that more than 80% had been previously excluded from school^3^.

##### Academic achievements

Evidence suggests that periods of exclusion may have detrimental effects on pupils’ learning outcomes. Exclusion is accompanied by missed academic activities, alienation, and demotivation in relation to academic goals (Brown, 2007; Michail, 2011). In particular, Hemphill et al., (2006) found that excluded pupils were slightly more prone to fail in the academic curriculum when compared with non‐excluded students (*OR* = 1.3, *95% CI* 1.1 to 1.5, *p*< .01). Along similar lines, Arcia, (2006) produced a longitudinal retrospective study regarding the associations between exclusions and achievements from fourth to seventh grade. After three years, non‐excluded students displayed substantially higher reading achievement scores when compared with their non‐excluded peers. In fact, seventh‐grade students who were excluded for 21 days or more achieved scores similar to fourth‐grade students that had not been excluded. Finally, Raffaele‐Mendez, (2003) added that those excluded were also less likely to graduate from high school on schedule.

##### Future social inclusion

Some studies have pointed out that young people excluded from school can also register a high risk of becoming “Not in Education, Employment, or Training” (NEET) in the future. In 2007, Brookes, Goodall, & Heady stated that students who had been excluded were 37% more likely to be unemployed during adulthood. Spielhofer et al. (2009) showed that among individuals with long‐term status as NEET, the majority had previous experienced of exclusions and truancy. More precisely, Massey (2011) argued that approximately one out of two excluded children will be NEET within two years of their exclusion.

Research has also illustrated the long‐term implications of exclusion for society as a whole. In economic terms, the cost of excluding children from school places a demand on public resources. Although the literature on this matter is still limited, Brookes et al. (2007) produced a report regarding the costs of permanent exclusion in the United Kingdom. The analysis encompasses an estimation of costs for the individual as well as for the educational, health, social and criminal justice services. Overall the cost, in 2005 prices, of permanently excluding a student was estimated at £63,851 per year to society.

While there is a stark link between the aforementioned negative outcomes and school exclusion, these should not be regarded as causal. Notwithstanding decades of research on school exclusion and its impact on later behaviour, we are still at an initial stage for testing causal associations in these matters. The association between exclusion and these negative outcomes may simply reflect underlying behavioural tendencies that lead to conduct problems, exclusion and poor outcomes later in life – that is, the antisocial syndrome depicted by Farrington (1997). In fact, school exclusion and the behaviours described here as “negative outcomes” could be explained by the same underlying factors or personality traits characterising the syndrome.

Despite the lack of empirical support for a causal association, some criminological theories provide a plausible explanatory framework to understand the connection between punishment and the persistence of deviant behaviour. Labelling theory, for example, suggests that those punished (by exclusion) and labelled as “deviant” may start behaving in ways that conform to their newly formed self‐image: by limiting their interactions with integrated students, for example, and shunning conventional social systems such as school (Krohn, Lopes, & Ward, 2014, p. 179). Likewise, Sherman's defiance theory (1993) elucidates the circumstances in which a punishment can produce more antisocial behaviour, such as defiance, instead of compliance with rules. In his view, punishment can increase the prevalence, incidence or seriousness of future offending when offenders deny responsibility, and when they perceive sanctions as unfair, stigmatising and imposed by an illegitimate authority.

Finally, in addition to all these findings and the rationale around the negative outcomes linked to school exclusion, it is important to mention that, so far, there is no evidence demonstrating that exclusion is effective for improving school discipline (Skiba, 2014). What is more, in the short term, exclusion seems to directly deny students’ right to access education as well as reducing adult supervision for those who are most at risk of further deviant behaviour, or most in need of teachers’ support.

### 1.3 THE INTERVENTION

#### 1.3.1 School‐based programmes

The prevalence of exclusion and its adverse correlated consequences have caught the attention of policy makers and programme developers. As a result, a range of interventions have been designed and implemented to improve school discipline. In the present review, we include different types of school‐based intervention aimed at reducing school exclusion as a punishment for inappropriate behaviour. These interventions include those targeting individual risk factors or school‐related factors, as well as those using a more comprehensive strategy that includes parents, teachers, school administrators, and the community.

Interventions targeting individual risk factors include, for instance, cognitive‐behavioural approaches such as anger management programmes or skills training for children (e.g., Humphrey & Brooks, 2006). Another type of intervention focusing on student behaviour – or, more precisely, students’ skills for conflict resolution – are restorative justice programmes (e.g., Schellenberg & Parks‐Savage, 2007; Shapiro, Burgoon, Welker, & Clough, 2002)In general, these interventions target motivated children and train them in practical skills to deal with anger, solve conflicts or become more assertive in social relationships. Such interventions are normally organised within a curriculum and implemented during school hours. The curriculum involves a package of group or one‐to‐one sessions using a wide range of techniques such as instruction, modelling, role‐play, feedback, and reinforcement, among others (Gottfredson, Cook, & Na, 2012; Schindler & Yoshikawa, 2012).

At the classroom level, interventions may target teachers’ abilities in classroom management (Pane, Rocco, Miller, & Salmon, 2013). The training for teachers encompasses instructional skills, such as guidelines for teaching rules and maintaining attendance, and non‐instructional skills, such as group management techniques, reinforcing positive conduct, and techniques to explain expected behaviour. Both skill sets are aimed at improving the learning process, preventing misbehaviour and encouraging positive participation by pupils (Averdijk, Eisner, Luciano, Valdebenito, & Obsuth, 2014).

Some schools offer mental health services independently or via community agencies. Experienced clinicians are located in schools in order to deliver individual, group, and/or family therapy. Clinicians may also be available for teacher consultation on matters related to students’ behavioural and emotional issues. All these interventions may target a reduction in out‐of‐school exclusion (Bruns, Moore, Stephan, Pruitt, & Weist, 2005).

Alternatively, comprehensive prevention strategies target students, families, teachers and school managers as well as the community as a whole (Bradshaw, Waasdorp, & Leaf, 2012; Flay & Allred, 2003; Colin Pritchard & Williams, 2001; Snyder et al., 2010). A well‐known comprehensive programme is the School‐Wide Positive Behavioural Interventions and Supports (SWPBIS). The programme aims to provide support for positive conduct by building proactive school‐wide disciplinary procedures (i.e., improving school climate and reducing problem behaviours). SWPBIS incorporates a multi‐level approach: from whole school prevention, to group‐based intervention for problematic pupils, and personalised, tailored interventions for high‐risk students. The basic elements of the programme are: i) building a school culture for both social and academic attainment, ii) early prevention of problem behaviours, iii) teaching social skills to all students, iv) using behaviour support practices, and v) actively using data for decision‐making. Research reports promising results, although further and stronger evaluation designs need to be undertaken (Gottfredson et al., 2012; Maag, 2012).

##### Previous reviews

In 2013/14 we conducted a systematic search of reviews and meta‐analyses assessing the effectiveness of school‐based programmes for promoting early prevention of risks (Averdijk et al., 2014). The results suggested there had been no previous meta‐analysis aimed at assessing the effectiveness of different types of interventions for reducing disciplinary school exclusion. Probably the most similar study is one published by Burrell, Zirbel, & Allen, (2003) who conducted a meta‐analysis on the effectiveness of mediation programmes in educational settings. Among many other outcomes, the analysis suggested that these interventions had a desirable effect (r = ‐.287, k = 17, N = 5,706, p < .05) on administrative suspensions, expulsions and disciplinary actions. However, in this meta‐analysis suspension was reported along with other disciplinary actions, and the study did not compare mediation with any other intervention (as proposed in the present meta‐analysis). The authors also call for a cautious interpretation given the high heterogeneity of primary results. A similar type of analysis was followed by Durlak, Weissberg, Dymnicki, Taylor, & Schellinger, (2011) and Gottfredson, Wilson, & Najaka, (2002). In both studies, school exclusion was coded as an outcome, but the final meta‐analysis did not report on the impact of the intervention specifically in relation to this targeted outcome.

Likewise, Solomon, Klein, Hintze, Cressey, & Peller, (2012) conducted a meta‐analysis exclusively testing the effectiveness of School‐Wide Positive Behavioural Interventions and Supports (SWPBIS) programme. Despite a small number of included studies reporting data on exclusion, the review does not report effect sizes by measuring their increase/decrease. Rather, the review reports effect sizes on the reduction of office discipline referrals and problematic behaviour.

In addition, two narrative reviews have recently been produced looking at intervention as a means of reducing disciplinary exclusion. Spink, (2011) explored qualitative, quantitative and mixed methods studies. Overall, 10 reports were found. The review concluded that multi‐agency interventions were the most frequent and that they could have a positive effect on reducing exclusion of pupils who are at risk. As expected, the study did not report a meta‐analysis of effect sizes. In 2012, Johnson produced another narrative review identifying programmes that may be an alternative for suspension in school systems. The search strategies were not clear enough to allow replication and, again, the nature of the design does not allow for the calculation of effect sizes.

### 1.4 WHY IT IS IMPORTANT TO DO THE REVIEW

Despite a growing body of research on the negative side effects of exclusion, no previous meta‐analysis based on a comprehensive systematic review has been conducted to synthesize evidence assessing the impact of school‐based interventions in reducing disciplinary exclusion. The current review addresses this gap by meta‐analysing results from existing published and unpublished studies, providing a statistical assessment of the overall effect of school‐based interventions at reducing exclusion.

This meta‐analytic investigation has clear implications for policy making. The results provided by the present study would produce a much‐needed evidence base for school managers, policymakers and researchers alike. These results can contribute to tackling the adverse developmental, social and economic effects of school exclusion mentioned in the previous pages, as well as potentially identifying alternative and less punitive approaches to school discipline.

## 2. Objectives

The main goal of the present research is to systematically examine the available evidence for the effectiveness of different types of school‐based interventions for reducing disciplinary school exclusion. Secondary goals include comparing different types of interventions (e.g., school‐wide management, classroom management, restorative justice, cognitive‐behavioural interventions) and identifying those that could potentially demonstrate larger and more significant effects.

We also aim – potentially – to run analysis controlling for characteristics of *participants* (e.g., age, ethnicity, level of risk); *interventions* (e.g., theoretical bases, components); *implementation* (e.g., facilitators’ training, doses, quality), and *methodology* (e.g., research design).

The research questions underlying this project are as follows:

- Do school‐based programmes reduce the use of exclusionary sanctions in schools?
- Are some school‐based approaches more effective than others in reducing exclusionary sanctions?
- Do participants’ characteristics (e.g., age, gender, ethnicity) affect the impact of school‐based programmes on exclusionary sanctions in schools?
- Do characteristics of the interventions, implementation, and methodology affect the impact of school‐based programmes on exclusionary sanctions in schools?

## 3. Methods

### 3.1 TITLE REGISTRATION AND REVIEW PROTOCOL

The title of the present review was registered in The Campbell Collaboration Library of Systematic Reviews on January 2015. The final version of the review protocol was approved in November 2015. The title registration and the respective protocol are available at: https://www.campbellcollaboration.org/library/reducing‐school‐exclusion‐school‐based‐interventions.html

### 3.2 CRITERIA FOR CONSIDERING STUDIES FOR THIS REVIEW

#### 3.2.1 Research design

Our original proposal was to include both randomised controlled trials and high‐quality quasi‐experimental studies (defined as studies using a comparison group, pre‐post testing and a statistical matching approach). To be eligible for inclusion, we stated that manuscripts must clearly report the method used to ensure equivalence between treatment and control groups, taking into account major risk factors (e.g. behavioural measures) and demographic characteristics.^4^

In this review, we only present results from randomised controlled trials (RCTs). There were three reasons for our decision:

1. First, even though a number of quasi‐experimental studies initially fulfilling our inclusion criteria were found by our searches, many of them fail to report baseline measures (e.g., Guardino, 2013; Hawkins, Catalano, Kosterman, Abbott, & Hill, 1999; Munoz, Fischetti, & Prather, 2014), the matching procedures were not described (e.g., Hasson, 2011; Risner, Vanderhaar, Muñoz, & Abati, 2005; St James‐Roberts & Singh, 2001) or the balance procedures did not produce statistical equivalence (e.g., Gao, Hallar, & Hartman, 2014).
2. A number of the school‐based intervention programmes included in this review presented several studies, involving quasi‐experiments as well as RCTs. Some examples involve interventions such as the Positive Action Program or the School‐Wide Positive Behavioural Interventions and Supports (e.g., Lewis et al., 2013; Snyder et al., 2010). In both cases, RCTs (e.g., Lewis et al., 2013; Snyder et al., 2010) were preceded by quasi‐experimental studies (Barrett, Bradshaw, & Lewis‐Palmer, 2008; Flay & Allred, 2003) . In this context, we decided to keep the strongest study design.
3. RCTs are regarded to be the most compelling methodological design to test the impact of a particular treatment. This type of study has the strengths of isolating confounding factors, reducing the likelihood of alternative explanations for observed effects (Shadish, Cook, & Campbell, 2002; Sherman, Farrington, Welsh, & Mackenzie, 2002). We believe that by selecting only these studies we will achieve a more precise final estimation of the effect of school‐based interventions.

To offer a broad overview of the research testing the impact of school‐based intervention at reducing school exclusion, a list of the quasi‐experimental studies can be provided on request.

Qualitative studies were excluded from the present review as stated in the published protocol.

#### 3.2.2 Types of participants

The present review is focused on the general population of students in primary and secondary schools irrespective of nationality, ethnicity, language, and cultural or socio‐economical background. By targeting primary and secondary schools, participants could theoretically be aged from 4 to 18 years of age.

Reports involving students who presented special education needs, disabilities or learning problems but were educated in mainstream schools were included in this review. However, reports involving students with serious mental disabilities or those in need of special schools were excluded. The rationale for this is that the results of this review are intended to be generalisable to mainstream populations of students in non‐specialised schools from all the included countries.

Students in college or higher levels of education have been excluded. Their exclusion from the review is based on previous evidence suggesting the largest number of exclusions affect pupils aged about 10 to 15 (e.g., Liu, 2013; Raush & Skiba, 2004; DfE, 2012).

#### 3.2.3 Included interventions

We include interventions defined as school‐based: that is, delivered on school premises, or supported by schools with at least one component implemented in the school setting. In the present review, we include interventions explicitly aimed at preventing/reducing school exclusion or those measuring exclusion as an outcome.

Interventions in the present review cover a wide range of psychosocial strategies for targeting students (e.g., Cook et al., 2014), teachers (e.g., Ialongo, Poduska, Werthamer, & Kellam, 2001), or the whole school (e.g., Bradshaw, Waasdorp, & Leaf, 2012). Types of intervention include, for example, those focused on:

- instructing students to identify risky behaviours and expanding their alternatives for responding appropriately to risks or harms (e.g., social skills training)
- developing teachers’ skills to improve the quality of their classroom management (e.g., reward schemes)
- cognitive‐behavioural treatment, such as anger management, counselling, social work, and mentoring programmes;
- school‐wide interventions.

Since there was no previous review analysing school‐based prevention programmes for reducing exclusionary discipline, we wanted to include a wide range of school‐based interventions that could be effective for reducing exclusionary practices.

#### 3.2.4 Excluded interventions

We excluded studies where the intervention was not school‐based or school supported. Even though some of these interventions targeted school students, they were community programmes or purely focused on mental health issues without any connection to schools (e.g., Henderson & Green, 2014; Schwartz, Rhodes, Spencer, & Grossman, 2013; Wiggins et al., 2009).

We also excluded interventions designed for children or adolescents who have committed a crime, that is, specialised interventions aimed at reducing reoffending or reconviction. Although suspended students may commit offences, such specialised interventions were excluded from the present review because they exceed the strategies used by schools to prevent misbehaviour and their levels of complexity make them too specific for a general population of students. School‐based prevention programmes targeting outcomes related only to students’ physical health (e.g., AIDS/ HIV prevention programmes, programmes to develop healthy eating programmes) were also excluded.

#### 3.2.5 Types of outcome measures

##### Primary outcomes

Eligible studies addressed school exclusion as an outcome. As mentioned in the background section, school suspension or exclusion is defined as an official disciplinary sanction imposed by an authority and consisting of the removal of a child from their normal schooling. This removal happens as a reaction to student behaviour that violates the school rules. We included studies testing fixed or permanent, long‐term or short‐term suspension as well as in‐school and out‐of‐school suspensions.

We excluded studies testing other disciplinary sanctions implemented in schools if they do not share the criteria described above. For instance, we excluded disciplinary sanctions such as loss of privileges, extra work, break/lunch detention, and after‐school detentions. These interventions do not involve exclusion from school or exclusion from regular teaching hours, and as such they are not covered by this review.

##### Secondary outcomes

For any identified study that reported findings on school exclusion as an outcome, we also coded the effects of the intervention on specific behaviour domains, focusing on internalising (e.g., inhibition, social withdrawal, anxiety or depression) and externalising (e.g., defiant or delinquent behaviours or aggressive behaviours such as bullying)problem behaviour (Achenbach & Edelbrock, 1979; Achenbach, 1978; Farrington, 1989)

By coding secondary outcomes, we aimed to assess the extent to which reductions in problem behaviour are a mediator of treatment effects on school exclusion. Indeed, interventions may affect exclusion in two different ways. The first is by improving behaviour that might otherwise lead to an exclusionary measure. The second possibility is that behaviour stays the same, but that the school develops an alternative strategy to deal with the disciplinary problems.

#### 3.2.6 Included literature

Databases and journals were searched from 1980 onwards with the aim of comprising more contemporary interventions or prevention programmes. Eligible studies included both published and unpublished book chapters, journal articles, government reports, and Doctoral theses. When the same data was published in more than one source (e.g., a book chapter and a journal article) we used all the linked manuscripts but the most complete report or the report measuring suspension was defined as the main source of data (see Section 3.4.5). That way we kept as much information as possible from a specific study but avoided over‐estimation of effect sizes. In cases where it was not clear if the manuscripts referred to the same study, we contacted the main author for further information (e.g., email communication with Barnes, Bauza, & Treiber, 2003; Bradshaw et al., 2012; Lewis, Romi, & Roache, 2012; Snyder et al., 2010; Sprague, Biglan, Rusby, Gau, & Vincent, 2016).

### 3.3 SEARCH METHODS FOR IDENTIFICATION OF STUDIES

The electronic searches were conducted between September 1 and December 1, 2015. In order to reduce the effect of publication bias, an attempt was made to locate the most complete collection of published and unpublished papers.

#### 3.3.1 Electronic searches

Below we list details of the 27 electronic databases searched. As noted above, these databases included both published (e.g., ISI web of knowledge, PsycINFO) and unpublished reports (e.g., Dissertation Abstracts, EThOS) as well as reports from Latin‐American countries (e.g., Scientific Electronic Library Online – SciELO).

**Table 2:** Electronic searches

**Databases**
1. Australian Education Index (AEI) 2. British Education Index (BEI) 3. The Campbell Collaboration Social, Psychological, Educational and Criminological Trials Register (C2‐SPECTR) 4. BMJ controlled trials 5. CBCA Education (Canada) 6. ClinicalTrial.gov 7. Criminal Justice Abstracts 8. Cochrane Central Register of Controlled Trials (CENTRAL) 9. Database of Abstracts of Reviews of Effects (DARE) 10. Dissertation Abstracts 11. Educational Resources Information Centre (ERIC) 12. EThOS (Beta) 13. EMBASE 14. Google 15. Google Scholar 16. Index to Theses Database 17. Institute of Education Sciences ‐ What Works Clearinghouse 18. ISI Web of Knowledge 19. MEDLINE 20. The National Dropout Prevention Centre/Network 21. The Netherlands National Trial Register (NTR) 22. Open Grey 23. Psych INFO 24. Sociological Abstracts 25. Social Sciences Citation Index (SSCI) 26. Scientific Electronic Library Online (SciELO). Electronic database collecting scientific production from developing countries (Spanish and Portuguese) 27. World Health Organisation International Clinical Trials Registry (WHO ICTRP)

For each database, we ran pilot searches including the key terms described in Table 3. Four categories of key words were used, including: i) type of study; ii) type of intervention; iii) population; and iv) outcomes. The pilot searches were useful to adjust the terms, synonyms and wildcards as appropriate. They were also helpful in creating combinations of terms that capture relevant sets of studies in each database.

**Table 3:** Key words for searches

**Type of study**	**Interventions**	**Population**	**Outcomes**
Evaluation Effectiveness Intervention Program Programme Programme effectiveness Impact Effect Experimental evaluation Quasi‐experimental evaluation RCT Random evaluation Efficacy trial	Disciplinary methods Token economy Classroom management program/ intervention/ strategies School management Early interventions School support projects Skills training	Schoolchildren Pupils Children Adolescents School‐aged children Student Youth Adolescent Young people	School exclusion Suspension Out‐of‐school suspension In‐school suspension Out‐of‐school exclusion In‐school exclusion Suspended Expelled Expulsion Outdoor suspension Stand‐down Exclusionary discipline Discipline

We kept a record with the date of searches, number of reports found, number of reports retrieved, key terms included, synonyms, and wildcards used when appropriate. Further details of electronic searches are presented in Section 13.

#### 3.3.2 Other resources searched

As planned, we contacted key authors requesting information on primary studies that could potentially be integrated in this systematic review and meta‐analysis. We also reviewed reference lists of previous primary studies or reviews related to the intervention/outcomes (e.g., Burrell, Zirbel, & Allen, 2003; Gottfredson, Cook, & Na, 2012; Johnson, 2012; Mytton, DiGuiseppi, Gough, Taylor, & Logan, 2006; Wilson, Tanner‐Smith, Lipsey, Steinka‐Fry, & Morrison, 2011).

### 3.4 DATA COLLECTION AND ANALYSIS

#### 3.4.1 Selection of studies

Eligible studies met the following criteria:

- Reported results of interventions from 1980 onwards
- Tested the impact of a school‐based intervention on different types of exclusion (e.g., in‐school, out‐of‐school, expulsion)
- Included students from primary and secondary school levels settled in mainstream schools
- Based on an experimental design, where participants are randomly allocated treatment or control conditions
- Reported statistical results for computed an effect size

#### 3.4.2 Data extraction and management

Data extraction was the responsibility of two researchers (AC & SV). Descriptive data of all studies potentially includable in the meta‐analysis was extracted using the data collection instrument presented in Section 12.2. The instrument facilitated the extraction of the following information:

- Bibliographical data (e.g., type of publication, year of publication, name of the publication, main author discipline)
- Ethics (e.g. declaration of conflicts of interest, use of informed consent)
- Research methods (e.g., type of design, units of randomisation, unit of analysis, variables used for matching)
- Sample selection (e.g., methods to select sample, attrition)
- Primary outcome coding (e.g., type of exclusion, duration of exclusion)
- Secondary outcomes coding (e.g., internalising and externalising behaviours, name of the instrument used to measure the outcome data)
- Base‐line measurements (e.g., source of data, quantitative measure of the primary outcome)
- Programme delivery (e.g., programme deliverer, training, type of intervention, frequency of the intervention)
- Post‐intervention and follow‐up measurement (e.g. official records, surveys)
- Data for calculation on effect sizes

The same two researchers extracted data for effect‐size calculations. The process was carried out independently. In general, discrepancies were solved by agreement but when the information reported was contentious, we asked for input from the more senior members of the team (ME & DF). Details on the data extracted from each included report can be found in section 9.1, 9.2 and 9.3 of the present review.

When data for calculation of effect sizes was incomplete we used two different strategies. First, we tried to find more details in other sources (e.g., published protocols or reports). Secondly, the lead researcher or members of the research team were contacted regarding the additional data needed.

Endnote X7 software was used to manage references, citations and documents. Data extracted to characterise studies was inputted in STATA v.13 in order to produce inferential/descriptive statistics. Effect sizes were inputted in Version 3.0 of the Comprehensive Meta‐Analysis software.

#### 3.4.3 Strategy to test inter‐rate reliability

To check code consistency across studies, or inter‐rate reliability, we use Cohen's Kappa coefficient (Cohen, 1960). In the event of two coders making inclusion/exclusion decisions, Cohen's Kappa coefficient computes a standardised index across studies based on cross‐tabulated ratings. The index is given by the difference between the observed percentage of agreement in ratings across studies (*P_o_*), and the probability of expected agreement due to chance (*P_e_*), divided by *1‐P_e_*.

Cohen's Kappa was calculated based on the following formula:

(1) $$k=\frac{Po-Pe}{1-Pe}$$

To a good approximation, we calculated the standard deviation of Cohen's Kappa, following the expression (Cohen, 1960):

(2) $$SD_{k}=\sqrt{\frac{P_{o}(1-P_{o})}{{\left(1-P_{e}\right)}^{2}}}$$

#### 3.4.4 Assessment of risk of bias in included studies

We planned to assess the risk of bias in included studies by using two different instruments. In the case of RCTs, we intended to use the Cochrane Collaboration risk of bias tool (Higgins & Green, 2011). To analyse risk of bias of studies involving quasi‐experimental designs we planned to use the ACROBAT‐NRSI, another Cochrane Risk of Bias Assessment Tool for Non‐Randomised Studies (Stern, Higgins, & Reeves, 2014). Both instruments were supposed to assist the research team in evaluating the external validity of the included reports.

At the end of January 2016, we began the assessment with the originally proposed instruments. We soon realised that the instruments and their categories seemed more suited to medical trials than school‐based experiments. Therefore, possible alternatives were explored. In consultation with the coordinating group editor, we selected the EPOC risk of bias tool suggested in the methods section of the Campbell Collaboration website (see the tool in section 12.3). The instrument proposes the following eight criteria for the assessment of quality bias:

- Sequence generation
- Allocation concealment
- Baseline outcome equivalence
- Baseline characteristics equivalence
- Incomplete outcome data
- Blinding of outcome assessment
- Protection against contamination
- Selective outcome reporting

Each of these domains was judged on a 3‐point scale (i.e., low risk, high risk, unclear risk). EPOC tool provides guidance and examples for each domain that facilitate the decision of assigning low, high or unclear risk. Two members of the team performed the assessment of risk of bias (AS & SV). Assessment of bias was performed independently and the final results represent the agreement of both evaluators.

#### 3.4.5 Criteria for determination of independent findings

Since violations of the assumptions of independence in meta‐analysis would lead to incorrect estimates of the variance for pooled effect sizes (Higgins & Green, 2011b; Romano & Kromrey, 2009),we used some strategies to deal with dependency in the data extracted from primary studies.

First, since we included book chapters, journal articles, government reports, and academic PhD theses, we anticipated the case where the same results would be published in more than one source (e.g., a book chapter and a journal article). The protocol stated that in those cases we would code only one outcome (e.g., the most complete, or the most outdated). In practice, we excluded 11 reports whose results were reported in more than one publication. They are grouped in four cases:

- The study did not report enough statistical data for effect size calculation. For instance, Vincent, Sprague, Pavel, Tobin, & Gau, (2015) did not report enough data for meta‐calculation. Although the main author was contacted, we were not able to access more details. For that reason we decided to include Sprague, Biglan, Rusby, Gau, & Vincent, (2016). This latter case used the same data but reported enough information for meta‐analysis. There was no overlap.
- RCT data was merged with quasi‐experimental data. For instance, Allen & Philliber, (2001) merged the RCT sample with another sample of students. As a result, the study was not an RCT anymore (For further details see Allen & Philliber 2001, p. 641).
- The same results were reported in more than one manuscript. That was the case of Panayiotopoulos & Kerfoot. The results of the study were reported in two papers (2004 and 2007). Results were identical in both publications (same dataset, same analysis and same outcomes). There was no overlap.
- The study did not report the outcome measured (i.e., suspension). A case in point is Arter, (2007). The author reported the results of her thesis in a journal. The journal article did not describe the outcome suspension, probably because no effect was found. We included the thesis since it reported all the outcomes measured.

Based on that, those 11 manuscripts were not included in the meta‐analysis. There was no overlap among them, consequently, no dependence in the outcomes was observed.

Secondly, included studies reported multiple time points, for instance, multiple follow‐up measures. The inclusion of multiple follow‐ups would create statistical dependence because the different measures are based on the same subjects (i.e., correlated with each other). We calculated effect sizes separately for those studies reporting short‐term and long‐term follow‐up measures. We also corrected variances estimation (see 3.4.8).

#### 3.4.6 Measures of treatment effect

We use Standardised Means Differences (SMD or Cohen's *d*) to measure the treatment effects of the school‐based interventions included in the review. The decision to use this specific effect size is based on the fact that most of the included manuscripts report results where measurements are expressed in continuous scales (see section 9.3). The standardised mean effect size for a *non‐clustered* study is given by

(3) $$d=\frac{{\bar{X}}_{e}-{\bar{X}}_{c}}{Sp},$$

where ${\bar{X}}_{e}$ and ${\bar{X}}_{c}$ represent the experimental and control group means, respectively, *S_p_* is the pooled sample standard deviation given by

(4) $$S_{p}=\sqrt{\frac{(n_{e}-1)s_{e}^{2}+(n_{c}-1)s_{c}^{2}}{\left(n_{e}-1)+(n_{c}-1\right)}}$$

where *n_e_* and *n_c_* are the sample size in each group, and $s_{e}^{2}$ and $s_{c}^{2}$ are the experimental and control group standard deviation, respectively.

#### 3.4.7 Issues with the unit of analysis

In the present review, we anticipated the inclusion of primary studies involving individually randomised as well as cluster‐randomised unit (e.g., schools or classrooms). One key issue emerges when meta‐analyses include cluster‐randomised studies: participants nested in the same cluster tend to be more similar to one another (as measured by the intra class correlation – ICC). Furthermore, when units of randomisation are clusters instead of individuals, we need to deal with the fact that the data presents different levels of variation (i.e., within clusters variation, between clusters variation and the total variance). This issue needs to be taken into account when computing effect size estimates. When this correlation is not accounted for, standard errors, confidence intervals and p‐values will tend to be too small. These conditions affect the meta‐analysis in two different ways. Firstly, the primary trial gets a mistakenly large weight. Secondly, the pooled result produces estimated effect sizes with an overly small standard error (Borenstein, Hedges, Higgins, & Rothstein, 2009; Higgins et al., 2011).

For the case of clustered data with dichotomous outcome measures (e.g., odds ratios), we followed the strategy proposed by the Cochrane Handbook for Systematic Reviews of Interventions (Higgins & Green, 2011), which corrects standard errors of effect sizes. The handbook suggests that the effective sample size in a cluster‐randomised trial can be obtained by dividing the original sample size by the design effect, which is calculated via

(5) 1+(M−1)×ICC.

In this equation, M is the *average* cluster size (units per cluster) and ICC is the intra‐cluster correlation coefficient. Once we were able to identify the *design effect*, the squared root of the design effect could be multiplied by the original standard error of the log Odds Ratio. Since ICC is rarely reported in primary studies, we have assumed a value of .05, based on the review of multiple meta‐analyses testing similar populations, produced by Ahn, Myers, & Jin, (2012).

In the case of clustered studies with continuous outcomes (e.g., school level means and standard deviations), we followed the strategy suggested by Hedges (2007) and Spier et al. (2013). Effect sizes were computed using *d_T2_* assuming equal cluster sample size:

(6) $$d_{T2}=\left[\frac{{\bar{\bar{X}}}_{■}^{E}-{\bar{\bar{X}}}_{■}^{C}}{S_{T}}\right]\;\sqrt{1-\frac{2(n-1)\rho }{N-2}}$$

In this equation ${\bar{\bar{X}}}_{■}^{E}$ and ${\bar{\bar{X}}}_{■}^{C}$ represent the overall means of the experimental and control group and *S_T_* is the total sample standard deviation estimated from the pooled sample standard deviation across the experimental and the control group. Rho (is the notation used to represent the intra class correlation. *N* is the total sample size and the sample size of the clusters is represented by *n*. Based on the characteristics of our data and following Spier et al., (2013) we assume equal cluster size in our calculations.^5^ When the clusters have different sizes, we will take a conservative approach, including the smallest cluster size in our calculation.

The variance of the effect size will be calculated by

(7) $$\begin{array}{c} V\{d_{T2}\}=\left(\frac{N^{E}+N^{C}}{N^{E}N^{C}}\right)\;(1+(n-1)\rho )+\\ d_{T2}^{2}\left(\frac{\left(N-2\right)(1-\rho ){\left(1-\rho \right)}^{2}+n(N-2n)\rho^{2}+2(N-2n)\rho (1-\rho )}{2(N-2)[(N-2)-2(n-1)\rho ]}\right) \end{array}$$

In this equation, *N^E^* and *N^C^* represent the experimental and control group sample across clusters. As suggested by Higgins & Green, (2011), in the event that the value of *ρ* is not reported, analysts are advised to assume a reasonable value based in previous studies with similar population (Ahn et al., 2012). As detailed previously, we have assumed a value of (*ρ*)= .05.

#### 3.4.8 Dealing with missing data

In those reports where key statistical information was missing, we attempted to obtain data from principal investigators. When that was not possible, the study was excluded from calculations of effect sizes. All the studies excluded for this reason have been identified and systematically reported in section 4.1.3.

#### 3.4.9 Time points within a study

In the present review, many of our selected studies involved repeated measures of the outcome exclusion. In fact, manuscripts reported measures of exclusion at baseline and post treatment (e.g., Hawkins, Doueck, & Lishner, 1988) or posttreatment and follow‐up (e.g., Farrell, Meyer, & White, 2001). In these cases, we have calculated a synthesis index or effect size of the difference, representing the change between those different measures (i.e., time points). Consequently, the change in exclusion from the baseline is computed by subtracting the means (*X*) as follows:

(8) $$Y_{diff}=X_{2}-X_{1}$$

One issue arises when pursuing this strategy. Because measures at baseline and posttreatment are positively correlated, the calculation of the variance must be corrected. If we avoid the correction, assuming the two measures to be independent (correlation equals zero), we could be overestimating the variance and underestimating the precision of the difference (Borenstein et al., 2009). For a fair approximation of the value of the variance, we would need to know the correlation between the pre‐ and post‐measures (covariation). However, the covariance is not usually reported in primary research (and this was commonly the case in our set of included studies). Consequently, we proceed to assume a value for that correlation. After checking previous meta‐analysis of similar populations, testing school‐based interventions with estimates for the stability of serious problem behaviours (e.g., Farrington & Ttofi, 2009; Wilson et al., 2011), we concluded that the value of the pre‐post correlation should be assumed to be equal to .75. We then calculated the variance of the difference by using equation 9 below (Borenstein et al., 2009), where V_1_ and V_2_ represent the variances of the original point estimates and *r* represents the pre‐post correlation value:

(9) $$VY_{diff}=V_{1}+V_{2}-2r\sqrt{V_{1}}\sqrt{V_{2}}.$$

As suggested by Higgins & Green, (2011), we undertook sensitivity analyses to determine whether the overall result of the analysis is robust in the use of imputed correlation coefficient.

#### 3.4.10 Assessment of heterogeneity

We report weighted mean effect sizes, under a random model using 95% confidence intervals and accompanied with graphical representation (i.e., forest plots). For investigating heterogeneity, we use the estimates suggested by Borenstein et al. (2009), specifically; Tau‐squared, *Q‐statistic* and *I^2^*.

Tau‐squared, or the difference between the total variance or variance observed and the within‐studies variance, will be estimated and reported.

The final calculation of the *Q*‐statistic includes reporting its value, degrees of freedom and *p*‐values. Significant *p*‐values provide evidence of heterogeneity in intervention effects.

Bearing in mind that *Q* can appear distorted when the number of studies meta‐analysed is small (Higgins, Thompson, Deeks, & Altman, 2003), we also report *I2. I2* “is the proportion of observed dispersion that is real rather than spurious” (Borenstein et al., 2009). High percentages will be interpreted as an indication of high heterogeneity, meaning that the study‐to‐study dispersion is due to real differences in true effect size and not attributable to random error.

#### 3.4.11 Data synthesis

Since our review has a wide scope, we use the random effect inverse variance weighted models for meta‐analytical calculations. The random effect model is the most appropriate when effect sizes are not homogeneous or consistently coming from a single population (Borenstein et al., 2009). Under a random effects model the variance includes the original (within‐studies) variance plus the between‐studies variance, *Tau^2^*.

Following Chandler, Churchill, Higgins, Lasserson, & Tovey, (2013), effect sizes will be coded such that a positive effect will reflect the outcomes favouring the treatment group. To illustrate our analysis, we provide summary forest plots displaying the estimated effect sizes along with their 95% confidence intervals.

#### 3.4.12 Subgroup analysis and investigation of heterogeneity

In the present review, we use moderator analysis involving categorical variables estimating models analogous to ANOVA. Analyses are run under a random‐effect model assuming separate variance components for each group. Meta regression has been run in order to explore heterogeneity.

#### 3.4.13 Outliers

The distribution of SMD effect sizes was examined to determine the presence of outliers. Following Lipsey & Wilson, (2001), outliers were defined as those values which are more than two standard deviations from the overall mean of effect sizes. One outlier was detected (i.e., Collier, 2002) and it was windsorised to the next closest value (Lipsey & Wilson, 2001).

#### 3.4.14 Sensitivity analysis

Since the present meta‐analysis involved a wide range of decisions, we conducted sensitivity analysis to test the robustness of these decisions (Higgins & Green, 2011). Specifically, we ran sensitivity analysis for the pre‐post correlations (i.e., covariance) assumed to be .75. We re‐ran the analysis using a correlation equal .50. As expected there was no change in the effect sizes and no relevant difference in standard errors. We also ran sensitivity analysis testing the impact of the outlier and the impact of the windsorisation (see section 4.7).

As stated in our protocol, we ran sensitivity analysis testing differences between published and unpublished reports.

#### 3.4.15 Duration of follow‐up

Included studies reported multiple time points data, for instance, multiple follow‐up measures. Since the inclusion of multiple follow‐ups would create statistical dependence due to the different measures based on the same subjects (i.e., correlated with each other), we calculate effect sizes separately for those studies reporting short‐term (i.e., post‐treatment) and long‐term (i.e., follow‐up) measures.

## 4. Results

### 4.1 DESCRIPTION OF STUDIES

#### 4.1.1 Results of the searches

**Figure 1:**
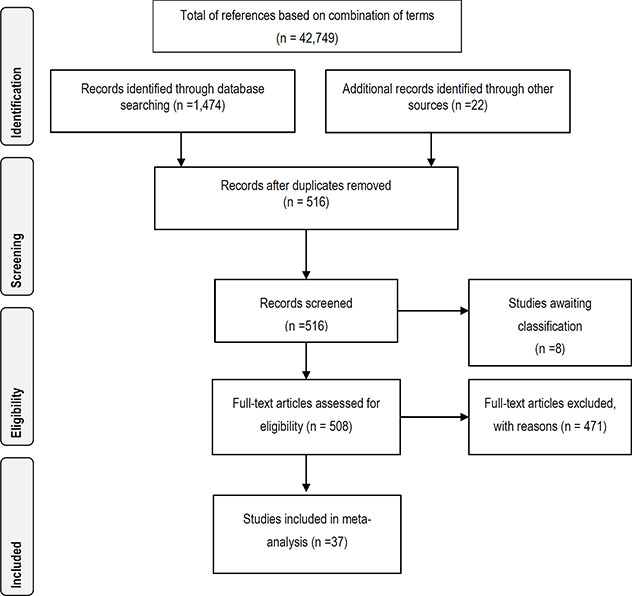
PRISMA flow^6^ chart of searches

We attempted to identify and retrieve the body of published and unpublished studies that met our inclusion criteria. Figure 1 shows a PRISMA flow diagram describing the results of our searches.

At the beginning, different combination of terms produced a total of 42,749 references from different electronic databases, of which we kept 1,474 relevant hits.

The 1,474 hits were selected based on screening the title, abstract and key words. We targeted studies broadly defined as evaluations, testing the impact of interventions on school exclusion.

As originally planned, we complemented electronic searches for papers with two extra strategies: i) reviewing lists of references from retrieved manuscripts, and ii) communication with key authors. Based on these steps, an additional 22 manuscripts were added to our sample. The full list of studies and their references were imported into Endnote X7. After removing duplicates, a total of 516 unique manuscripts were saved for further assessment.

Efforts were made to retrieve the full text copies of all 516 selected manuscripts. Notably, a high percentage of them corresponded to unpublished reports (48.6%), mainly PhD theses from American universities and Technical or Governmental reports. In the end, we were able to retrieve almost all full text manuscripts. Only eight studies out of 516 were categorised as “studies awaiting classification” and they are reported in Table 6.

The next round of evaluation was based on reading the information available in abstracts, methods and results sections using the eligibility checklist (see Section 12.1). The checklist included the basic criteria for inclusion in this systematic review. We concluded the second round of evaluation with 471 manuscripts excluded for reasons laid out in section 4.1.3. Thirty‐seven papers presented enough statistical data for inclusion in our meta‐analysis.

#### 4.1.2 Inter‐rater reliability

Two trained researchers (AC & SV) independently assessed the 516 pre‐selected manuscripts for inclusion or exclusion. We calculated *Cohen's Kappa* for testing inter‐rater reliability (Cohen, 1960; Sim & Wright, 2005). The value of *Kappa* ranges between 0 and 1, where a value of 1 represents perfect agreement between the two raters and a value of 0 indicates no more agreement than that expected by chance. We obtained a *Cohen's Kappa*= .76; *SD*=.81; *SE*=.036, reflecting a high level of agreement between coders. After calculating the agreement between coders, they went through the papers where they found disagreement. Differences were solved by further analysis and discussion. When needed, a more senior member of the team was consulted.

#### 4.1.3 Excluded studies

Following our published protocol, we excluded a total of 471 manuscripts. Table 4 summarises the reasons for the exclusion of each report. References of the excluded papers are availablein section 7.2.

**Table 4:** Synthesis of the reasons for the exclusion of 471 papers

**Reason for exclusion**	***k***	***%***
Outcome measure was absent	52	11.0
Type of intervention	53	11.2
Methodological design	339	72.0
Participants	5	1.1
Time span	5	1.1
Pilot study	1	0.2
Reports based on the same data	11	2.3
Not enough data for meta‐calculations	5	1.1
Total	471	100

*Outcome*. We excluded 52 reports (11%) because they did not present a suitable measure of school exclusion. In some specific cases the primary outcome was not reported (e.g., Gage, Sugai, Lewis, & Brzozowy, 2015; Webster‐Stratton, Rinaldi, & Reid, 2011) or it was reported in a composite measure along with other disciplinary measures which did not involve any type of exclusion from school (e.g., De Blank, 2009; Wright, Offord, John, Duku, & DeWit, 2005). Since it was not possible to isolate our primary outcome we excluded those reports. In other exceptional cases exclusion was measured as a predictor instead of an outcome (e.g., Rosenbaum, 2012).

*Type of intervention*. Following our protocol, we excluded 53 (11.2%) studies because the tested intervention was not delivered in schools, supported by schools or with at least one component implemented in school settings. Consequently, we excluded community programmes when they had no alliance with a school (.e.g., Henderson & Green, 2014; Wiggins et al., 2009). Alternative schools for high‐risk students (e.g., Rhea, 2010)and intervention in special schools (e.g., Kutash, Duchnowski, Green, & Ferron, 2013) were also excluded. Since our protocol stated that targeted intervention must be an “alternative to school exclusion”, we dismissed studies testing for instance the impact of restorative justice in the context of an in‐school exclusion programme (e.g., Brown‐Kersey, 2011), or conflict resolution in the context of an in‐school exclusion programme (e.g., Devlin, 2006). In both cases, the tested intervention was delivered in addition to exclusion more than being an alternative to it.

*Methodological design*. 339 (72%) studies were excluded because they did not satisfy the methodological characteristics defined in the protocol. We excluded studies lacking a control group (e.g., McDaniel‐Campbell, 2011; Nocera, Whitbread, & Nocera, 2014) and those studies where the control group was not equivalent in demographics and risk factor variables (e.g., Kilian, Fish, & Maniago, 2006; May, Stokes, Oliver, & McClure, 2015).

During the searches, we kept 28 manuscripts which corresponded to literature reviews (e.g., Fronius, Persson, Guckenburg, Hurley, & Petrosino, 2016; Gonzalez, 2012), systematic reviews (e.g., Wolgemuth, Cobb, & Dugan, 2007) or meta‐analysis (e.g., Noltemeyer & Ward, 2015) related to school exclusion or behavioural problems in schools. These types of manuscripts were initially retained on the understanding that they could be a source for identifying extra primary research reports. All 28 of these studies were excluded in the second round of assessment once we had checked their citation lists.

We identified nine manuscripts evaluating the impact of obligatory use of school uniform on levels of school exclusion (i.e., Draa, 2005; Gentile & Imberman, 2012; Gouge, 2011; Johnson, 2010; Samuels & Bishop, 2003; Shimizu & Peterson, 2000; Stevenson III & Brooks II, 1999; Vaughan, 2001; Washington‐Labat & Ginn, 2003). Even if they represented a particular type of intervention that could be interesting to meta‐analyse and compare, none of these eight interventions presented the research design targeted by our review. For that reason, they were excluded.

Finally, under the method design criteria we excluded a number of qualitative studies (Huston, 1999; Maguire, Macrae, & Milbourne, 2003; Rose, 2008) and case studies (e.g., Navarro, Aguilar, Aguilar, Alcalde, & Marchena, 2007) since none of them contributed with statistical data for meta‐analysis.

*Participants*. Five reports (1.1%) presented data focused on students with special needs (e.g., Cramer, 1990), or young offenders (e.g., Parkes, 2008). Since these participants were not targeted in our protocol, all these reports were excluded from our analysis.

*Time span*. Five studies (1.1%) were excluded because they were published before 1980 (i.e., Rogers, 1972)or because they reported the evaluation of an intervention implemented before 1980 (i.e., Feldi, 1980; Fisher, 1980; Herzog, 1980; Safer, Heaton, & Parker, 1981).

*Reports based on the same dataset*. Finally, we excluded 11 reports (2.3%) because they presented additional results based on the same data reported elsewhere (e.g., Allen & Philliber, 2001; Arter, 2007; Panayiotopoulos & Kerfoot, 2007; Vincent, Sprague, Pavel, Tobin, & Gau, 2015). In these cases, we kept the most complete report and the additional manuscripts assisted in a better understanding of the included research; they were however defined as excluded.

*Pilot study*. We excluded a single report presenting data from a pilot study (Bonell et al., 2015). In page 12 of the cited report, the author states that the aim of the pilot was “to evaluate feasibility and acceptability of the intervention and trial methods, and not to estimate intervention effects.” For those reasons, even though it was reporting results, the study was excluded. We kept references to the report in the category of ongoing research (see section 4.1.4).

*Not enough data for meta‐calculations*. Five reports (1.1%) were excluded because they did not present enough data for calculation of effect sizes (e.g., Grinage, 2005).

#### 4.1.4 On‐going studies

We identified the protocol of four ongoing studies whose outcomes had not been published at the end of the searches in December 2015. As observed in Table 5, all of them are cluster randomised control trials testing the impact of school‐based interventions and measuring school exclusion as an outcome.

**Table 5:** On‐going studies

**Author**	**Design**	**Sample**	**Outcome of interest**	**Intervention**
Acosta (2015)	Cluster‐RCT	Unclear (US)	Suspension or expulsion	The Restorative Practices Intervention (RPI)
Bonell et al. (2014)	Cluster‐RCT	40 schools (UK)	Temporary and permanent school exclusion	INCLUSIVE (combines changes to the school environment, promotion of social and emotional skills and restorative practices)
Philliber (2015)	Cluster‐RCT	6 schools (US)	In‐ and out‐of‐school suspension	School‐wide positive behavioural interventions and supports (SWPBIS)
Eiraldi (2014)	Cluster‐RCT	12 school (US)	School suspension	Teen Outreach Programme in Kansas City (replication)

#### 4.1.5 Studies awaiting classification

We were unable to classify eight studies. We selected them based on title, abstract and key words but we have not been able to locate the full text copies. A list of studies that could be potentially included in a future updated version of this review is given in Table 6.

**Table 6:** Details of studies awaiting classification

**Author**	**Type of publication**	**Design**	**Sample**	**Outcomes**	**Intervention**
Allen (1981)	Report	Pre‐post design. It is unclear if the study uses random allocation of participants.	12 seventh grade teachers	Disciplinary referrals Corporal punishment School suspensions	Positive approach to discipline (PAD) is a system of classroom management, incorporating counselling, problem‐solving, and time‐out centres.
Forbes (1996)	Thesis	Pre‐post design. It is unclear if the study uses random allocation of participants.	900 students (grades six to eight)	School infractions Out‐of‐school suspension	Social skills training
Foster (2011)	Book	No information	African American boys in elementary classrooms	No information	Social Skills Curriculum
Gaines & Schram (2005)	Conference proceedings	Intervention is given to some students, but not to others. Unclear if it is RCT.	Middle and high school students	School absences Suspension/expulsion Disciplinary actions Attendance Grades	School Probation Officer Programme is aimed at identifying juveniles who may be at‐risk of engaging in delinquent behaviour
Gallegos (1998)	Book	Review of interventions	Unclear	Suspensions Expulsion Dropout school	Not given
Neise (1983)	Thesis	Two treatment groups and one control group. Unclear if they were randomly allocated in conditions	37 middle school students	The Devereaux Adolescent Behaviour Rating Scale (DABRS) The Behaviour Rating Scale (BRS) Seventh hour In‐school suspension Out‐of‐school suspension	Group counselling methods that used interpersonal problem solving strategies versus non‐directive counselling
Norris (2009)	Conference proceedings	Evaluation	Unclear	Suspension Expulsion	Restorative justice
Spillman (1993)	Thesis	Unclear	Ninth grade students	Achievement Motivation Attendance Suspension rate	Interdisciplinary teaming and parent contacts

#### 4.1.6 Included studies

Thirty‐seven studies reporting 38 interventions’ effect sizes were included in this meta‐analysis. As we mention before, all of them were randomised controlled trials. In 23 studies the control group received no‐treatment (62.2%); six studies reported controls receiving intervention or business as usual (16.2%), four experiments offered a placebo to the control group (10.8%) and four studies allocated controls in a waiting list (10.8%).

Following sections provide a general overview of 37 included studies. Data is organised by characteristics of the included studies, characteristics of participants and characteristics of the delivered interventions.

##### Characteristics of the included studies

We included studies presenting interventions (*M_date_* =2003; *SD*=9.5) carried out and reported between 1980 and December 1, 2015 when we finished our searches. Exceptionally the review involves three manuscripts published in 2016. In the first case, Obsuth et al., (2016), the registered protocol of the study had been identified in our electronic searches and we were waiting for the published version, which was released in March 2016. The second case involves two different papers: Okonofua, Pauneskua, & Walton, (2016) and Sprague, Biglan, Rusby, Gau, & Vincent, (2016). Both studies were sent to us from key authors in the field. As they were recent studies, matching our inclusion criteria, we decided to retain them in our analysis. No other study was sent to us after the end of the searches in December 2015. Figure 10.1 (in appendix) presents the distribution of studies per year. Results show that the number of RCTs has increased over the specific years involved in this report.

As shown in Table 7, we included published and unpublished reports in almost equal proportion (51% published versus 49% of unpublished reports). All of them were written in English, and represent studies implemented in the United States and the United Kingdom only. Although we explored global databases, in particular databases from Latin‐American countries we were not able to find studies conducted in other locations.

**Table 7:** Characteristics of studies included in meta‐analysis

**Study characteristics**	*** ***	*** ***
**Publication year (range)**	1980 ‐2016	
** **	*** ***	*** ***
**Type of publication**	***n***	***%***
Journal articles (published)	19	51.3
PhD thesis, technical report (unpublished)	18	48.6
		
**Language**	***n***	***%***
English	37	100
		
**Country of the sample**	***n***	***%***
United States	33	89.1
United Kingdom	3	8.1
Unclear	1	2.7
		
**Author's main discipline**	***n***	***%***
Education	13	35.3
Social work	1	2.7
Psychology	12	32.3
Criminal justice	1	2.7
Psychiatry/medicine	6	16.2
Econometrist‐economics	2	5.4
Not reported	2	5.4
		
**Declared conflict of Interest in published studies (N=19)**	***n***	***%***
Yes	7	36.8
No	12	63.2
		
**Conflict of financial Interest**	***n***	***%***
Unlikely	18	48.6
Possibly	13	35.1
Likely	2	5.4
Not enough information	4	10.8
		
**Unit of randomisation**	***n***	***%***
Individuals	26	70.3
School clusters	8	21.6
Classroom clusters	3	8.1
		
**Statistical analysis**	***n***	***%***
Multilevel modelling	4	10.8
Differences in means (MANOVA, *X^2^*, ANOVA, ANCOVA)	22	59.4
Regression	8	21.6
Frequencies	3	8.1
		
**Exclusion measurement**	***n***	***%***
Self‐report	2	5.4
Teacher‐report	3	8.1
School records (official records)	30	81.1
Unknown	2	5.4
** **	*** ***	*** ***
**Effective^28^ sample size**	***n***	**%**
< 300	24	64.8
Between 300 and 800	7	18.9
> 900	6	16.2

We coded data on the main author's discipline. Our data shows that more than 60% of studies testing interventions intended to reduce exclusion have been carried out by researchers in the fields of Education and Psychology.

Interestingly, only seven studies (36.8%) of experimental evaluations published in peer‐reviewed journals disclosed a personal or organisational Conflict of Interest (CoI). This percentage is coherent with the findings of Eisner, Humphreys, Wilson, & Gardner, (2015) who found limited attention to full CoI disclosure in the evaluation of psychosocial interventions. They argue that even if “transparency about CoI in itself does not necessarily improve the quality of research, and researchers with a CoI should not be presumed to conduct less valid scholarship, transparency is needed for readers, to assess the study findings and their particular context” (Eisner et al., 2015, p. 10).

In addition to the presence/absence of CoI statements, we evaluated studies on their potential conflict of financial interest (CoFI) by using a scale developed by Eisner & Humphreys, (2012). It is a trichotomous scale that helps to identify three levels of conflict. The three categories in the scale are defined as follows: i) Unlikely conflict of interest: none of the study authors are programme developers or licence holders; ii) Possible: a study author is a programme developer or collaborator with a programme developer AND the programme is not (yet) commercially available OR the business model is ‘not‐for‐profit’; or iii) Likely: study author, is a programme developer or collaborator with a programme developer AND programme is commercially available AND business model is ‘for‐profit’. For details on the instrument see section 12.4.

We found 18 studies (48.6%) where the CoFI was defined as ‘unlikely’. Essentially, in this set of studies, none of the programme evaluators were involved (i.e., directly or as a collaborator) in the development of the intervention or were licence holders. However, we found 13 studies (35.1%) where we assessed a ‘possible’ CoFI. In those cases, the evaluator was a programme developer/deliverer or a previous collaborator with a programme developer; the programme was not commercially available, or the business model was defined as ‘not‐for‐profit’. Finally, only two studies (5.4%) in our evaluation transparently declared information that allowed us to classify them as ‘likely’ to present a potential financial conflict of interest. In both cases the authors reported that one of the members of the evaluation team was related to the holder of a licensed programme evaluated. The evaluation of each study is reported in section 9.2.

In terms of methodological design, all included studies were randomised controlled trials. While 70.3% of our studies randomised individuals, almost 30% (11 studies) randomised clusters of students, that is, entire schools (i.e., Bradshaw et al., 2012; Cornell, Allen, & Fan, 2012; Lewis et al., 2013; Obsuth et al., 2016; Snyder et al., 2010; Sprague et al., 2016; Ward & Gersten, 2013; Wyman et al., 2010) or classrooms within schools (i.e., Brett, 1993; Farrell, Meyer, & White, 2010; Ialongo, Poduska, Werthamer, & Kellam, 2001). Where necessary, we corrected data in clusters in order to combine it with individual level data. For further details on corrections, see section 9.3.

The most frequent analysis of the data was differences in means such as chi‐squared, ANOVA, ANCOVA or MANOVA (59.4%) followed by regression (21.6%), and with a minority of the studies running Multi‐level analysis (10.8%).

Additionally, measures of disciplinary exclusion were mainly based on official records (81%) provided by schools or other official institutions. Table 7 offers a general description of studies’ sample size. The average sample size was M_size_=1,168 (SD=3107.3) participants. But this average should be cautiously interpreted since the included studies range widely from 20 to 13,498 participants. This is an important issue for the statistical power of the calculated impacts.

#### Characteristics of participants in included studies

As observed in Table 8, in the present meta‐analysis, sampled students most frequently come from the higher school grades (i.e., middle and high schools), with a *M_age_=*12.9; *SD*= 2.8.

**Table 8:** Characteristics of participants included in meta‐analysis

***Population's characteristics***		
**Study average age**	*mean* 12.9	*standard deviation* 2.8
**Grade**	*frequency*	*%*
Elementary school	11	29.7
Middle school	16	43.2
High school	8	21.6
Mixture of levels	2	5.4
** **		
**Ethnicity**	*mean*	*standard deviation*
% Black or Afro‐American	54.1	37.2
% White	24.6	27.9
% Latino	20.2	25.5
		
**Free school meals**	*mean*	*standard deviation*
	66.2	23.9

Note. Means and standard deviations are calculated at study level.

In addition, data shows that students were nested in schools with a high percentage of Black and Latino students and, subsequently, with a low percentage of White‐Caucasian peers (24.6%). In fact, five of the included studies are based in schools where a 100% of the pupils were Black (i.e., Barnes, Bauza, & Treiber, 2003; Brett, 1993; Collier, 2002; Mack, 2001; Reese, Murphy, & Filipczak, 1981).

As stated by Hobbs & Vignoles, (2010) and Sutherland, Ilie, & Vignoles, (2015), in this report the percentage of students with access to free school meals was understood as an index of the vulnerability of the population. Across our selected studies the pupils receiving meals funded by the school was predominant (*M_fsm_* =66.2; *SD*=23.9%).

#### Intervention characteristics: dosage, delivery and targeted change

The present review includes a wide range of school‐based interventions covering, for instance, strategies such as counselling, mentoring, community services, classroom management, after‐school academic support, and school‐wide strategies, among others. Taking a broad view, 27 % of the interventions were focused on a change at the level of the school or teacher, and a 73% anticipating that a change in pupils’ skills/behaviours could affect suspension/exclusion rates.

As shown in Table 9, school staff (32.4%) or school staff assisted by external facilitators (24.3%) delivered most of the interventions. This means that schools interested in reducing exclusion typically put resources into the intervention process. Most of those delivering the interventions were school psychologists/counsellors (32.2%) and, in two particular cases (5.4%), the intervention was delivered by a police or probation officers, which seems to be an increasing trend in the United States (e.g., Gottfredson et al., 2012; Hirschfield, 2008).

**Table 9:** Characteristics of interventions included in meta‐analysis

Intervention characteristics	Frequency	%
At school level	10	27.0
At students’ level	27	73.0
** **	** **	** **
**Programme delivery**	** **	** **
External facilitators	9	24.3
School facilitators	12	32.4
School facilitators plus external facilitators	12	32.4
Missing	4	10.8
	** **	** **
**Who delivered programme?**	** **	** **
Social worker	2	5.4
Psychologist/counsellor	12	32.2
Teacher	9	24.3
Police/probation officer	2	5.4
Trained community agent	7	18.9
Not clearly stated	5	13.5
	** **	** **
**Role of the evaluator**	** **	** **
Deliver the programme	4	10.8
Design the programme	8	21.6
Delivery and design	3	7.8
Independent	18	48.6
Unknown	4	10.8
	** **	** **
**Is the programme curricular?**	** **	** **
Yes	25	67.6
No	12	32.4
** **	*** ***	*** ***
**Duration in weeks**	** **	** **
Less than 12 weeks	14	37.8
Between 13 and 24 weeks	4	10.8
More than 24 weeks	14	37.8
Unknown	5	13.5
	** **	** **
**Duration of intervention**	***m***	***sd***
Hours per week (*n*=25)	1.78	2.09
Number of weeks	20.4	11.5

*Note. For specific details regarding each included study, see section 9.1 in appendix*.

We coded data on the role of the evaluator. We identified four potential approaches taken by evaluators; i) delivering the programme, meaning that the researcher implemented the intervention as well as acting as evaluator (e.g., Harding, 2011); ii) designing the programme, meaning that the evaluator defined or took part in the defining the theoretical base, aims and activities of the intervention (e.g., Cornell et al., 2012), iii) both, designing and delivering the programme (Mack, 2001) and iv) independent evaluation, referring to those researchers or research teams where any member was involved in any stage of the designed or delivery (e.g., Bradshaw et al., 2012; Dynarski et al., 2003). A high percentage of interventions were designed and/or delivered by the same researcher who evaluated the impact of the intervention. Independent evaluators conducted 48.6% of the RCTs included in this review.

A high percentage of the interventions were curricular (67.6%). For instance, social skills trainings or anger management training were based on a pre‐designed curriculum with detailed aims and activities for each session. In the present review, non‐curricular interventions are those targeting school‐wide change or those focused on counselling and individual therapies.

On average, included interventions lasted M=20; SD=11.5 weeks. 37.8% of the interventions lasted less than 12 weeks and an equal percentage were delivered over more than 24 weeks. In general, school‐wide interventions lasted longer in our sample (i.e., 35 weeks or more).

#### 4.1.7 Description of the interventions

Table 10 provides a description of the included school‐based programmes targeting school exclusion as a primary or secondary outcome. In some specific cases this grouping could be restrictive, because some of the interventions involve multiple components, however we have attempted to create an exhaustive list of categories.

**Table 10:** Types of intervention programmes

**Programme**	**Number of effect sizes**	**Number of studies**	**% of studies**
Enhancement of academic skills	2	2	5.4
After‐school programmes	2	2	5.4
Mentoring/monitoring	5	5	13.5
Skills training for students	9	9	24.3
Skills training for teachers	4	3	8.1
School‐wide strategies	6	6	16.2
Violence reduction	3	3	8.1
Counselling, mental health	3	3	8.1
Other	4	4	10.8
Total	38	37	100

*Enhancement of academic skills*. We found two effect sizes targeting the enhancement of academic skills as a strategy in order to improve academic performance, increase motivation and promote more adaptive behaviour. Edmunds et al., (2012) tested an intervention to boost the academic progress of students in order to facilitate their future access to college while in the case of Cook et al., (2014), the intervention involved academic remediation plus social skills training.

*After‐school programmes*. Two effect sizes come from interventions that offered students after‐school activities. While the intervention tested by Dynarski et al., (2003) was more focused on academic support and recreational activities, Hirsch, Hedges, Stawicki, & Mekinda, (2011) tested an after‐school programme offering students paid apprenticeships.

*Mentoring/monitoring programmes*. Five effect sizes reported the impact of interventions focused on mentoring/monitoring. These programmes involved structured and supportive relationships between a young person who presents academic, emotional or behavioural difficulties and a non‐parental adult, their mentor. Mentoring entails a volunteer member of the community serving as a role model and providing support to a younger person over an extended period of time (e.g., Brett, 1993; Wyman et al., 2010). In other cases, adults served as tutors for the students, supervising their performance, providing advice or counselling, and assisting the students with academic tasks (Johnson, 1983; Peck, 2006; Reese et al., 1981). Such tutors were normally schoolteachers or school counsellors.

*Social skills training for students*. We found nine effect sizes representing the impact of social skills training. These programmes were based on social learning and cognitive behavioural theories (e.g., Burcham, 2002; Collier, 2002; Harding, 2011; Hostetler & Vondracek, 1995; Shetgiri, Kataoka, Lin, & Flores, 2011; Smith, 2004) and their goal is to enhance individuals’ socio‐cognitive, socio‐emotional, and behavioural skills in order to regulate maladaptive conducts. Social skills training programmes typically consist of a curriculum with focused training modules. Some more specific programmes target communication skills (e.g., Obsuth et al., 2016) or approaches to reducing stress (e.g., Barnes, Bauza, & Treiber, 2003). Group‐based sessions and occasionally one‐to‐one sessions (e.g., Russell, 2007) offer the opportunity to implement specific techniques (e.g., instruction, modelling, role‐playing, feedback and reinforcement) in a “real‐world environment”.

*Skills training for teachers*. We found four independent interventions targeting teachers’ skills. The training provides preventive strategies and techniques that help to maintain classroom discipline, create a supportive educational environment, and enhance students’ positive behaviour. These involve training in facilitating mutual respect between teacher and student (e.g., Okonofua, Pauneskua, & Walton, 2016) as well as training to establish clear classroom rules (e.g., Hawkins, Doueck, & Lishner, 1988). Skills for teachers also involve strategies for working in an alliance with parents to promote students’ engagement to the school activities (e.g., Ialongo et al., 2001).

*School‐wide interventions*. Six effect sizes represent comprehensive interventions targeting systemic changes across the whole school. They involve pupils, teachers, parents, and sometimes also the community where the school is based. These programmes aim to create positive environments, with clear rules that promote good behaviour, learning and safety. School‐wide interventions are capable of addressing the needs of schools as institutions as well as the particular needs of individual school children. We found six studies testing school‐wide strategies: Bradshaw et al., (2012); Cornell et al., (2012); Lewis et al., (2013); Snyder et al., (2010); Sprague et al., (2016); Ward & Gersten, (2013).

*Violence reduction*. We included three effect sizes measuring the impact of violence reduction programmes. Although these interventions could be classified as skills training, we have isolated them, because they are specifically targeted at increasing self‐control and reducing violence (e.g., Feindler, Marriott, & Iwata, 1984; Mack, 2001). We also included anger management programmes encouraging peaceful responses to conflict (e.g., Farrell et al., 2010).

*Counselling and mental health interventions*. We included three effect sizes primarily focused on the provision of counselling in schools (e.g., Berlanga, 2004; Tilghman, 1988)and on a more specialised provision from community mental health services (i.e., Panayiotopoulos & Kerfoot, 2004).

*Other interventions*. Four effect sizes were classified in this general category. They encompass a community services programme (Allen, Philliber, & Herrling, 1997), a multicomponent programme (Arter, 2005), a career awareness intervention (Bragdon, 2010), and a programme focused on character‐building education, promoting civic behaviour and national values (Crowder, 2001).

### 4.2 RISK OF BIAS IN INCLUDED STUDIES

**Figure 2:**
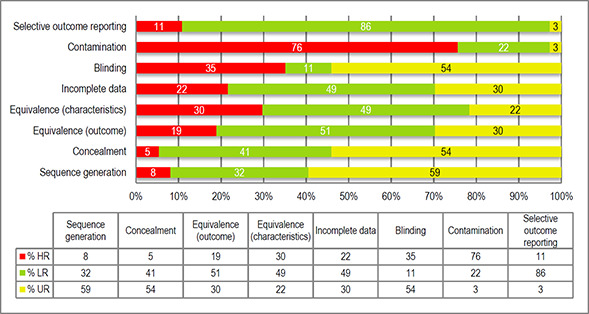
Risk of bias in included studies based on EPOC risk of bias tool *Note. Each of the eight evaluated criteria have been assigned one of three possible alternatives: high risk= HR; low risk=LR and unclear risk= UR, as expressed in the first column underneath the graph*.

The methodological quality of each publication included in the review was evaluated using the EPOC risk of bias tool (see section 12.3). The instrument evaluates the internal validity of reported results. Two coders (SV & AS) independently applied the EPOC tool to each study at different locations. The following results represent the agreed rating of both coders.^7^ Below, we report the results for each of the eight criteria involved in EPOC. Figure 2 shows a summary of the overall result, and Table 9.2 (section 9.2 in appendix), offers a detailed evaluation of each study.

#### Adequate sequence generation

Adequate sequence generation is intended to produce comparable groups in an experimental evaluation. Accurate methods used to generate the allocation are, for instance, the use of random number tables or a computer random number generator (Higgins & Green, 2011). Regardless of the selected methods, it should be clearly described in enough detail to allow an assessment of whether the sequence generation was adequate.

As shown in Figure 2, only three studies (8%) presented a high risk of bias with respect to allocation sequence. A case in point:Allen, Philliber, Herrling, & Kuperminc, (1997) used three different methods of randomisation (i.e., picking names from a hat, coin toss and using an alphabetical list of names) as well as running randomisation at different levels in the same study (i.e., individuals and classrooms). In our opinion this mixture of methods could lead to a high risk of bias in the final results: in particular, the use of a list of names is known not to be appropriate for allocating cases. In the case of Barnes, Bauza, & Treiber, (2003:2), the author mentions that “each school was alternately assigned to either TM or health education control.” Since the study involves only two schools, allocation would be predictable and at a high risk of bias.

Although we identify a low percentage of high‐risk cases, it is important to note that a large proportion of studies (59%) were categorised as “unclear risk”. This is because many reports presented succinct descriptions of the randomisation process without detailing the methods of sequence generation (e.g., Arter, 2005; Brett, 1993; Feindler, Marriott, & Iwata, 1984; Reese, Murphy, & Filipczak, 1981). 32% of manuscripts were defined as presenting a low risk of bias (e.g., Bradshaw et al., 2012; Hirsch et al., 2011; Lewis et al., 2013).

#### Allocation concealment

Correct allocation concealment safeguards a rigorous implementation of the randomisation process by not allowing researchers or participants prior knowledge of the results of assignment (i.e., by using sealed envelopes or other procedure that prevent knowledge about the condition that the participant is going to be allocated). To achieve this aim it has been suggested that allocation should be centralised and executed at the beginning of the study (Schulz & Grimes, 2002).

In the present meta‐analysis, only 5% of the studies were evaluated as having a high risk of bias for allocation concealment. Some examples of high‐risk studies are those where reports suggested that schoolteachers instead of researchers performed the random allocation (e.g., Crowder, 2001) or that the randomisation was performed after the participants’ screening (i.e., Mack, 2001). Even if we cannot be sure that the results of the screening biased the randomisation, this conduct could potentially interfere with the chances of participants being placed in the control or treatment group.

Once again, we found a high percentage of studies (54%) reporting minimal details of allocation concealment. In concrete terms, 20 out of 37 included reports were classified as “unclear risk” of bias. The remaining 41% (i.e. 15 studies) were classified as having a low risk of bias.

#### Baseline equivalence in the outcome measured

A key element of a randomised controlled trial is that it ensures, in theory, that participants (and their associated outcomes and characteristics) are distributed by chance in the control and treatment groups. Pre‐existing baseline differences between groups – particularly of outcomes – could suggest problems in randomisation, hence it is a key focus when assessing risk of bias (Shadish et al., 2002). It is important to mention that we did not focus on whether there were statistically significant differences between groups, as that is a function of sample size; instead we reviewed means and distributions.

We found seven studies (19%) whose description of the baseline equivalence suggested a high‐risk. This was found, for instance, in cases where the control group presented higher levels of exclusion than the treatment group and there was not clear mention of adjustment (e.g., Panayiotopoulos & Kerfoot, 2004). Another example concerns the imbalance of one specific type of exclusion: specifically, expulsion and out‐of‐school exclusion were equivalent in the treatment and the control groups, but display substantial imbalance in the case of in‐school exclusion (e.g., Berlanga, 2004).

Again, it is important to mention that nearly 30% of assessed studies reported limited information, and for that reason they were assigned an unclear risk of bias. The remaining 49% of our included randomised controlled trials were evaluated as low risk of bias.

#### Baseline equivalence in other participants’ characteristics

Regarding this criterion, the instrument considers the balance in demographics or any other behavioural outcomes, which again should be equivalent in the treatment and the control group if randomisation has been successful.^8^ We found 11 studies (30%) displaying non‐equivalent results between treatment and control groups. Studies reporting imbalance in gender (e.g., Berlanga, 2004), ethnicity (e.g., Hostetler & Fisher, 1997), initial levels of problem behaviour, self‐control or size of the school (e.g., Harding, 2011)were all classified as a potentially having a high risk of bias.

A further 22% (eight studies) did not report enough data for judgement (i.e., unclear risk) and around half (49%) of the studies were assessed as presenting a low risk of bias.

#### Addressing incomplete outcome data

The fact that our included studies pursued more than one measure across time makes it likely that attrition or other forms of missingnes affected sample sizes.

We assess a high risk of bias when i) substantial attrition was present in the study and the researchers did not mention a strategy to deal with that issue (e.g., Peck, 2006); ii) when they used list‐wise deletion (e.g., Russell, 2007), and iii) in those cases where the attrition affected the treatment or control group in an unequal proportion of missing cases across arms (e.g. Hostetler & Fisher, 1997). Using these criteria, we judged that eight studies (22%) presented a high risk of bias.

As in previous cases, a high number of studies (30%) did not report enough data to be judged. Seventeen studies (46%) were evaluated as presenting a low risk of bias. Low risk cases involved i) those studies reporting zero attrition; ii) studies where attrition was represented by a low percentage of cases; iii) when missingnes was equivalent in the treatment and the control group, and iv) when the researcher reported attrition, analysed it and used methods to deal with attrition (e.g., multiple imputation, full information maximum likelihood or intention to treat rather than assessing the effect of treatment on the treated).

#### Blinding of outcome assessment

This criterion covers bias arising from the fact that those collecting outcome data are aware of the condition assigned to each participant (e.g. individuals, classrooms, schools). In our evaluation, 35% or 13 of the coded studies were assessed as having a high risk of bias. We assigned a high risk of bias when those in charge of delivering the intervention were also collecting data (e.g., Harding, 2011) or when teachers rated the students’ behaviour while being aware of the allocated condition (e.g., Tilghman, 1988). Although the outcome was in the majority of studies, based on official records, we did not assume this meant blind assessment. In most cases, teachers or school staff who imposed exclusion were not necessarily blind to the allocated condition of participants. In any case, it must be said that in school‐based experiments blinding is likely infeasible. Most of the studies require at least a minimal participation of school staff and allocated condition is mostly evident for participants (Hutchison & Styles, 2010).

As in previous cases, the number of reports lacking the data to evaluate blindness of the outcome assessment (i.e., unclear risk) was high (54%) and only four studies (11%) were assessed as low risk bias.

#### Protection against contamination

Protection against contamination refers to the measures taken to avoid a spill‐over of the treatment into the control group. Specifically, contamination is the risk that the control group might accidentally receive the intervention, but also the risk that the two groups could influence each other's outcomes. If the groups cannot remain isolated during the experimental study, contamination would threaten the validity of the results. In our meta‐analysis 76% of the studies presented a high risk of contamination. The main explanation for high risk of bias was the fact that schools in the trials host both the treatment and the control participants (e.g., Cook et al., 2014; Smith, 2004).

One single study (3%) did not report enough data for evaluation and it was categorised as unclear risk of bias. Eight studies (22%) were classified as low risk of bias. Normally low risk studies were cluster‐randomised experiments where control and treatment participants were in different schools (e.g., Bradshaw et al., 2012; Snyder et al., 2010).

#### Selective outcome reporting

Selective outcome reporting occurs when there is a difference between the proposed outcomes for evaluation and those finally reported. In our evaluation, four studies (11%) presented a high risk of selective outcome reporting (e.g., Panayiotopoulos & Kerfoot, 2004; Russell, 2007). In the case of thesis or trials without published protocols, the assessment was only based on discrepancies between outcomes proposed and reported in those documents^9^. Thirty‐two studies (86%) displayed a low risk of bias and only 3% did not report enough data for judgement.

#### Summary

Overall, our assessment demonstrates that a high number of the included studies lacked enough information to use the EPOC tool to judge risk of bias in all areas. Specifically, 22 studies were not clear on how they allocated units to treatment and control groups. Similarly, blinding to allocation was another area infrequently reported with enough detail to allow assessment (i.e., in the case of 21 studies). These points aside, contamination or spill‐over is probably the main threat to the validity of results among the RCTs included in our review.

Arguably the seven methodological strongest studies were Bradshaw et al., (2012), Cook et al., (2014), Hirsch et al., (2011), Johnson, (1983), Lewis et al., (2013), Obsuth et al., (2016) and Wyman et al., (2010). All of them presented low risk in the randomisation process and most of them are clustered studies. In particular, these studies achieved low risk of bias in six or more of the eight EPOC criteria.

### 4.3 SYNTHESIS OF RESULTS

#### 4.3.1 Primary outcome: overall impact of school‐based intervention

The present analysis incorporates 38 effect sizes across 37 studies producing enough statistical information for meta‐analysis. These studies represent a total sample of 31,273 students (*M_age_* =12.5; *SD*=2.85) partaking in completed trials as treatment, control or placebo groups.

**Figure 3:**
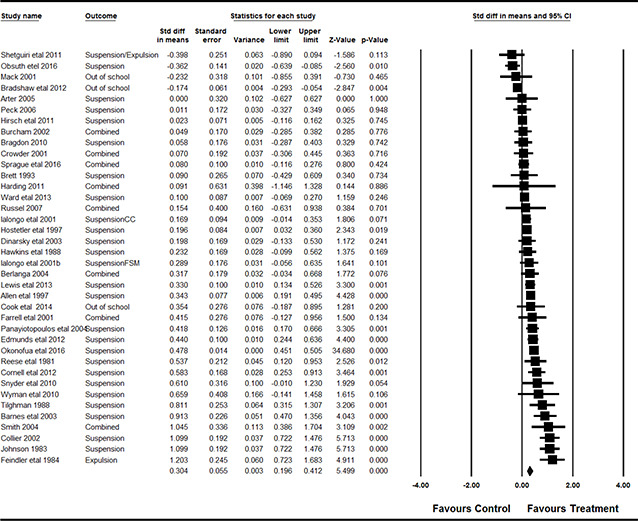
Forest plot of the effect sizes for the impact of school‐based programmes on school exclusion

On average, school exclusion was significantly reduced in the experimental group compared with the control group, post‐treatment (i.e.,sixmonths on average). Under a random effects model, the standardised mean reduction was SMD=.30; (*95% CI* .20 to .41; *p*<.001). Figure 3 shows that results are positive and statistically significant, meaning that those participating in school‐based interventions were less likely to be excluded than those in the control group. Results exhibit significant heterogeneity (*Q*=301.3; *df*= 37; *p*<.001; *I^2^* = 87.7; *τ^2^* =.078) which was expected in this meta‐analysis bearing in mind that we include different school‐based programmes, administering different “doses of treatment” with participants from different locations and in different school grades.

As suggested by Lipsey and Wilson (2001, p. 153) the U3 statistic for a SMD= .30 is 62%. This would indicate that 62% of the treatment group is above the median of the control group. Stated differently, if we assume a 50/50 success rate for both groups, the treatment group sees 62% success, versus 38% in the control group.

##### Long‐term effects

**Figure 4:**
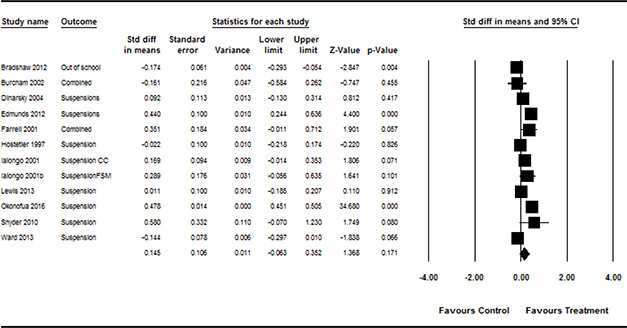
Forest plot of the effect sizes for the impact of school‐based programmes on school exclusion: long‐term effects

When we isolated studies measuring impact at follow‐up (i.e., 12 or more months after finishing the intervention), benefits of the interventions were less clear. In fact, Figure 4 demonstrate that the effect was reduced by half (SMD=.15; 95%CI ‐.06 to .35) and it was shown to be non‐statistically significant.

**Figure 5:**
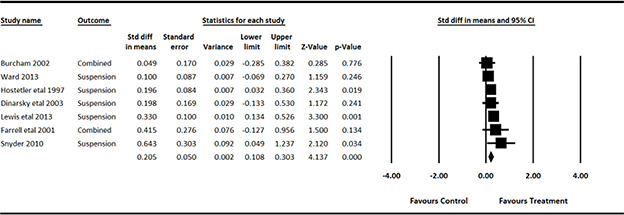
Forest plot of the effect sizes for the impact of school‐based programmes on school exclusion: post‐treatment (seven studies only)

**Figure 6:**
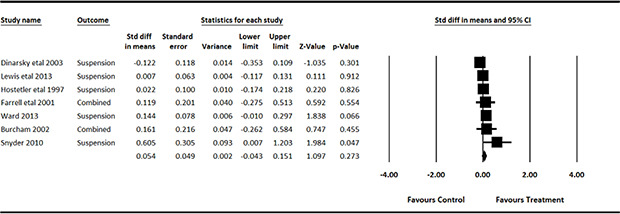
Forest plot of the effect sizes for the impact of school‐based programmes on school exclusion: follow‐up (seven studies only)

To increase precision in our results, we ran a meta‐analysis with a subset of studies reporting both post‐treatment and follow‐up measures. Only seven studies reported short and long‐term effect measures. As observed, the overall effect size at post‐treatment (Figure 5) and follow‐up (Figure 6) are lower than the ones initially reported but they follow the same direction.

At post‐treatment, under a random effect model, the standardised mean reduction was SMD=.21 (95%CI .11 to .30). However, when we ran the meta‐analysis including only the subset of seven studies, the average time involved under “post treatment” was 12 months. It implies that although the overall effect is slightly lower than the general measure reported in Figure 3, the impact lasts longer (i.e., 12 instead of 6 months on average). In the case of the effect at follow‐up, the subset of studies produced and overall impact that was almost null (SMD=.054; 95%CI ‐.04 to .15) and non‐significant. As shown in Table 11, heterogeneity was highly reduced when the analysis with the subset of studies was carried out.

**Table 11:** Summary of overall effect at post‐treatment and follow‐up (seven studies only)

**Impact**	**SMD**	**95% CI**	**n**	**k**	**Measure of Heterogeneity**
Post‐treatment	.21	(.11; .30)	7	7	*Q*=6.54;*df*=6;*p*>.05;*I* ^2^=8.2;*τ^2^* =.001
Follow‐up	.054	(‐.04; .15)	7	7	*Q*=7.80;*df*=6;*p*>.05;*I^2^* =23;*τ^2^* =.004

#### 4.3.2 Moderator analysis by type of exclusion

As proposed, we ran an analysis for the different outcome measures: in‐school exclusion, out‐of‐school exclusion, expulsion, and general exclusion. Results of the four independent meta‐analysesare summarised below in Table 12.

**Table 12:** Results of four independent meta‐analyses by type of disciplinary exclusion

**Type of Exclusion**	**SMD**	**95% CI**	**n**	**k**	**Measure of Heterogeneity**
In‐school	.35	(.11; .58)	6	6	*Q*=11.62;*df*=5;*p*<.05;*I^2^* =57;*τ^2^* =.045
Out‐of‐school	.02	(‐.16; .19)	9	9	*Q*=22.72;*df*=8;*p*<.05;*I^2^* =65;*τ^2^* =.041
Expulsion	.53	(.07; .98)	4	4	*Q*=13.66; df= 3p<.05; I^2^=78; τ^2^=.14
General measure	.32	(.21 .43)	27	28	Q=171.4; df=27 p<.001; I^2^=84; τ^2^=.056

*Note. n= number of studies; k=number of effect sizes*.

**Figure 7:**
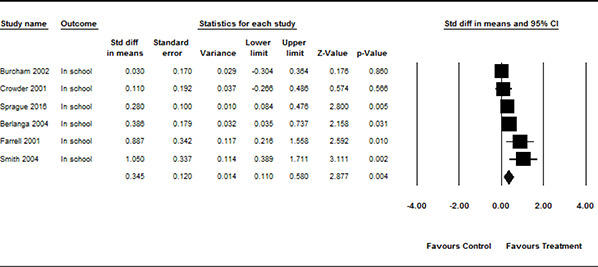
Forest plot of the effect sizes for the impact of school‐based programmes on in‐school exclusion

*In‐school exclusion*. Only six trials were concerned with the effect of school‐based intervention on reducing in‐school exclusion. Under a random model, the effect of the intervention remained positive and statistically significant. The final effect described in Figure 7 was SMD=.35 (*95% CI* .11 to .58; *p*<.005). Once again heterogeneity was tested, finding significant but smaller variability across studies (*Q*=11.62; *df*=5; *p*<.05; *I^2^* =57; *τ^2^* =.045).

**Figure 8:**
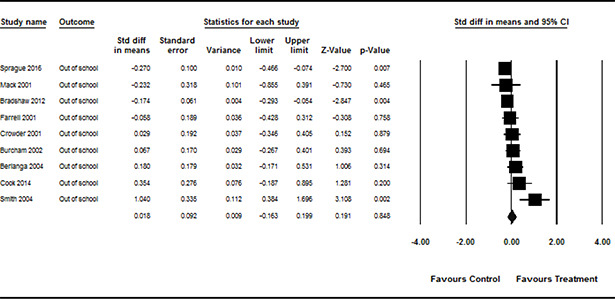
Forest plot of the effect sizes for the impact of school‐based programmes on out‐of‐school

*Out‐of‐school exclusion*. Correspondingly, nine studies reported data for the impact of interventions on out‐of‐school exclusion. Figure 8 shows an effect close to zero, non‐statistically significant of SMD=.02 (*95% CI* ‐.16 to .19; *p*>.05).

**Figure 9:**
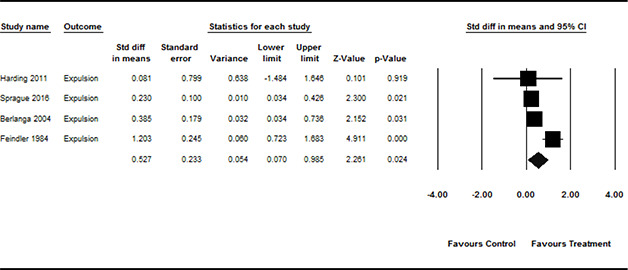
Forest plot of the effect sizes for the impact of school‐based programmes on school expulsion

*Expulsion*. The impact of school‐based interventions on expulsion was significantly higher than any other impact described so far. Figure 9 shows that expulsion was reduced by SMD=.53 (95% CI .07 to .98; *p*<.05) with significant heterogeneity (*Q*=13.66; *df*=3; *p*<.05; *I^2^* =78; *τ^2^* =.14) but based on only four reports presenting data for analysis. Therefore, these results must be evaluated cautiously.

*General Suspension*. Finally, a number of studies presented data on suspension as a broad and general measure. These studies did not describe operational definitions about the type of disciplinary suspensions involved in the outcome. We have defined this category as General Suspension. In the aim of transparency, we report these results separately, although this measure could involve any of the previous outcomes reported above and, therefore, be a subset of the overall effect size reported at the beginning of this section.

**Figure 10:**
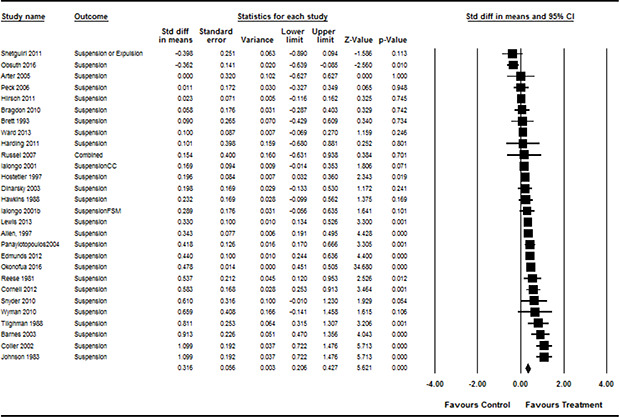
Forest plot of the effect sizes for the impact of school‐based programmes on general school suspension

Figure 10 shows 27 studies reporting 28 independent effect sizes concerned with the impact of targeted interventions on general suspension. The effect of school‐based interventions was positive SMD= .32 (95% CI .21 to .43; *p*<.001), it was statistically significant and similar to the overall effect size reported in Figure 3 above. In addition, heterogeneity remained substantial (*Q*=171.45; *df*=27 *p*<.001; *I^2^* =84; *τ^2^* =.056).

#### 4.3.3 Secondary outcome: overall impact of school‐based intervention on internalising behaviours

As stated in our protocol, for any identified study reporting data on school exclusion, we also coded a secondary outcome referring to internalising and externalising behaviours. Data on internalising behaviour was not very prolific in our set of included studies. Only five trials presented statistical results but sometimes the data was insufficient for effect size calculations. For that reason, we are not running meta‐analysis on internalising behaviours, but we summarise the results in Table 13.

**Table 13:** Impact of school based interventions on internalising behaviours

**Study**	**Sample**	**Measure**	**Statistical measures**
Bradshaw 2012	N=12,334	Emotion regulation	Students in SWPBIS school fared better in comparison with control schools. (*y*=.05; t=2.38; p<.05)
Harding 2011	N=43	Emotional Symptoms (SDQ)	Negative effect, not statistically significant. SMD=‐.30 (95%CI ‐.3 to .91); p>.05
Russell 2007	N=61	Internalising problems	On page 20, Table 4, the author presents means, standard deviations and sample size for treatment and control group. The author asserts that intervention reduced antisocial behaviour. Based on our calculations, that reduction is not significant (SMD=.32; 95%CI ‐.14 to .79; p>.05)
Tilgham 1988	N=100	Anxiety	The definition of the measure suggests that anxiety is part of a composite measure. Impact of treatment on anxiety is unclear (p.49).
Wyman	N=226	Assertive vs. withdrawn	Measure is described as a measure of anxiety (e.g.,“Nervous, frightened”). The programme has a positive effect on the internalizing behaviour ES=.37 (.03 to .71).

#### 4.3.4 Secondary outcomes: overall impact of school‐based intervention on externalising behaviours

We found a diverse range of measures referring to externalising behaviours, such as substance misuse, violence, aggression, and problematic behaviour in school. To pool together comparable measures, we ran a meta‐analysis only on behaviours that could be categorised as antisocial such as aggression, physical fights, delinquency, bullying and conduct disorder^10^.

**Figure 11:**
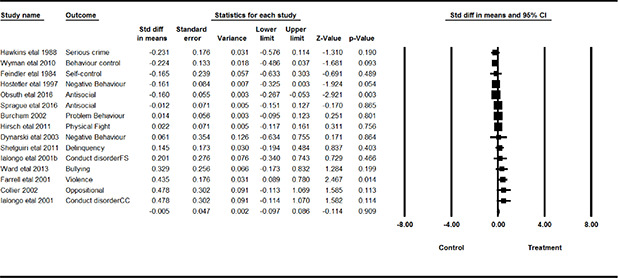
Forest plot of the effect sizes for the impact of school‐based programmes on antisocial behaviour

Fourteen studies reported complete data for a composite measure of antisocial behaviour (See Figure 11). The fourteen studies provided 15 independent effect sizes. Unusually, Wyman et al. (2010) reported a measure of behaviour control (e.g., children accepting imposed limits). We reversed the results as a proxy of antisocial behaviour and included this information in our calculations. The same procedure was followed with Feindler et al. (1984) who reported a measure of increase in self‐control. Once again, we reversed the effects size as a proxy of antisocial behaviour.

As reported in Figure 11, the overall impact of school‐based interventions on antisocial behaviour, under a random effects model, was not statistically different from zero SMD= ‐.005 (95% CI ‐.097 to .09; *p* >.05) indicating an overall null effect of these programmes at reducing antisocial behaviour.

It must be highlighted that some of the included studies in this overall measure reported negative effect sizes, meaning that in some specific cases the intervention had iatrogenic impact (Hawkins et al., 1988; Hostetler & Fisher, 1997; Obsuth et al., 2016). For instance, Hawkins et al., (1988) reported the impact of an intervention focused on interactive teaching and co‐operative learning targeting low achievers in mainstream schools. The treatment group showed a reduction in the number of exclusions but an increase in the mean value for serious crime. The study reported by Hostetler & Fisher, (1997)described a similar case. For its part, Obsuth et al., (2016) reported the results of a clustered randomized controlled trial testing the impact of “Engage in Education‐London”. The intervention targeted high risk students and it was aimed at improving communication and broader social skills. Results suggest that the iatrogenic effects were observable not only for exclusion but also for the case of antisocial behavior.

It is important to mention that Feindler et al., 1984; as well as Wyman et al., (2010) were reverted and used as proxies of antisocial behaviour. For this reason, they should not be understood as interventions which report iatrogenic results.

### 4.4 SUB‐GROUP ANALYSIS

A number of potential effect modifiers that could help to explain the expected heterogeneity in our results were initially identified in the published protocol. Based on previous research we pre‐defined moderators that covered four aspects, namely: i) participants’ demographic characteristics; ii) behavioural problems; iii) the theoretical basis of the interventions, and iv) the quality of the intervention. In this section we present sub‐group analysis results. The calculations have been run under a random effects model assuming a separate variance component. Forest plots with further details are reported in Section 11 (appendix).

#### 4.4.1 Effects moderated by participants’ demographic characteristics

Of the 38 effects reported in this meta‐analysis, 11 were tested in schools whose population was predominantly male (i.e., more than 60% of students were male), and 19 effects were tested in schools presenting a mixed population (i.e., neither gender exceeded 60 per cent).

Post‐intervention effects were different for both groups. For studies targeting predominantly male schools, the standardised mean difference was SMD=.41; (95% CI .10 to .72; *p*<.05). In studies targeting mixed‐gender schools, the impact was lower (SMD=.17; 95% CI .02 to .32: *p*<.05). Differences between groups were not statistically significant (*Q*=1.84; *df*=1; *p*>.05).

In terms of age, the best proxy variable was school grade. This information was reported in 34 studies and 35 independent effects. To test the hypothesis that effect sizes vary by age, we ran sub‐group analysis for 12 studies involving elementary school students (SMD=.27; 95%CI .09 to .45; *p*<.05), 16 targeting middle schools (SMD=.23; 95% CI .04 to .41; *p*<.05), and eight targeting high schools (SMD=.45; 95%CI .18 to .72; *p*=.001). The effect was statistically significant in each sub‐group and larger in high school populations. However, the between effect difference was not statistically significant (*Q*=1.81; *df*=2; *p*=.41) meaning that there is no evidence that the effect of school‐based interventions differs by age (i.e., grade at school) in the present meta‐analysis.

Ethnicity was reported as a continuous variable (i.e., percentage of each ethnic group). For that reason, the role of ethnicity in explaining the effect of school‐based programmes will be explored in a meta‐regression analysis.

#### 4.4.2 Effects moderated by participants’ behavioural problems

In our review, only a limited number of studies presented data on behaviour (i.e., internalising or externalising behaviours). In addition, the overall impact of school‐based interventions on antisocial behaviour was not statistically different from zero.

Pupils involved in included studies shared a similar high‐risk condition. They were registered in schools with a high percentage of ethnic minorities and more than 60% of the students in those schools received free school meals, which is an indicator of disadvantaged socio‐economic backgrounds. In that sense, participants do not display high variability.

#### 4.4.3 Effect of different school‐based programmes on school exclusion

One of the aims of the present meta‐analysis was to compare the effect of different interventions on the reduction of school exclusion. Table 14 presents the standardised mean differences, confidence intervals and p‐values as well as measures of heterogeneity for each of the ninetypes of programmes included in the review. The typology is the same as described in section 4.1.7.

**Table 14:** Effect size by type of school‐based intervention

**Type of Intervention**	**SMD**	**95% CI**	**p‐value**	***k***	**Measure of Heterogeneity**
Enhancement of academic skills	.43	(.25; .61)	*p*<.001	2	*Q*=.09;*df*=1;*p*>.05;*I^2^* =0;*τ^2^* =0
After‐school programme	.05	(‐.08; .17)	*p*>.05	2	*Q=.91; df=1; p>.05; I^2^>=0; τ^2^* =0
Mentoring/monitoring	.47	(.02; .93)	*p*<.05	5	*Q*=20.1;*df*=4;*p*<.001;*I^2^* =80;*τ^2^* =.21
Skills training for students	.31	(‐.05; .67)	*p*>.05	9	*Q*=60.4;*df*=8;*p*<.001; *I^2^* =86;*τ^2^* =.23
Skills training for teachers	.31	(.11; .52)	*p*<.05	4	*Q*=13.6;*df*=3;*p*<.05; *I^2^* =78;*τ^2^* =.03
School‐wide strategies	.20	(‐.03; .43)	*p*>.05	6	*Q*=34.8;*df*=5;*p*<.001; *I^2^* =86;*τ^2^* =.06
Violence reduction	.48	(‐.33; 1.3)	*p*>.05	3	*Q*=13.3;*df*=2;*p*<.001; *I^2^* =85;*τ^2^* =.44
Counselling, mental health	.46	(.23; .68)	*p*<.001	3	*Q*=2.65;*df*=2;*p*>.05;*I^2^* =25;*τ^2^* =.01
Other	.21	(.03; .39)	*p*<.05	4	*Q*=4.11;*df*=3;*p*>.05;*I^2^* =27;*τ^2^* =.01

First of all, as observed in Table 14,most of the interventions are represented by a restricted number of effect sizes, and for this reason, these results should be interpreted cautiously.

Secondly, the standardised mean differences of only five types of programmes present positive (small to moderate effect sizes) and statistically significant results in favour of the reduction of school exclusion. Those programmes are: i) Enhancement of academic skills, ii) Mentoring/monitoring, iii) Skills training for teachers, iv) Counselling/mental health services, and v) Other programmes. Since “other programmes” involve a mixture of different interventions we believe they cannot be interpreted in the same way as the remaining four types. Similarly, when it comes to the number of studies included in each sub‐group, it seems that the most stable results are Mentoring/monitoring and Skills training for teachers since they are based on a larger number of studies.

Thirdly, to test the hypothesis that differences were significant among the compared sub‐types, we ran further analysis. The comparison demonstrates that differences are statistically significant (*Q*=18.4; *df*= 8; *p*<. 05), meaning that variation in effect sizes can be explained by the type of intervention implemented.

#### 4.4.4 Theoretical bases

Reported information on the theoretical bases of the interventions was not very comprehensive. Selected studies described components of interventions more frequently than reporting the theory or set of theories framing the *praxis*. Based on the provided details, 20 interventions were clearly based on a cognitive behavioural frame, while another five were concerned with ecological ideas, targeting a change of the school system as a whole. The remaining effects refer to different theories such as emotional intelligence (i.e., Smith, 2004), empathy (i.e., Okonofua et al., 2016), civic values (i.e., Crowder, 2001) or developmental theories (i.e., Allen et al., 1997). A number of studies did not provide enough information to make a judgement. We tried to run sub‐group analysis on the theoretical bases of the interventions but it demonstrated low power, given that the number of effects was, in some cases, as low as one per sub‐group. In that scenario, we decided that a measure of the level of targeted change could inform more consistent data on the theory framing the interventions. We then divided the studies into those targeting a systemic change versus those targeting a change in pupils.

The standardised mean difference for the 10 evaluations targeting a change at school level provideda significant reduction with a value of SMD=.25 (*95%CI* .04 to .45), whereas the 28 evaluations targeting a change at the pupil's level reported a significant reduction with a value of SMD=.33; (*95%CI* .19 to .48). Both independent effects are statistically significant; however, the between‐group comparison reported non‐significant differences (*Q*=.48; *df*=1; *p*>.05) meaning that there is no evidence that the effect differs by level of targeted change.

#### 4.4.5 Moderator analysis: quality of implementation

Previous research demonstrates that well‐implemented programmes – those including training, monitoring and supervision – display larger and more consistent effect sizes (e.g., Durlak et al., 2011; Gottfredson & Wilson, 2003; Lösel & Beelmann, 2006). We tested this hypothesis based on two variables, namely, “training before implementation” and “monitoring during intervention”.

Twenty‐five studies (reporting 26 independent effect sizes) clearly stated the presence of training hours before the intervention was delivered (i.e., training hours for those delivering the intervention). In the remaining 12 studies, authors did not mention any kind of training. We ran sub‐group analysis to test the hypothesis that those that reported training could produce a significantly different effect. In the end, those reporting prior training yielded a result equal to SMD=.29 (95% CI .16 to .43; *p*<.001) whereas those that did not report training produced an effect equal to SMD=.34 (95% CI .15 to .53; *p*<.001). Both effects were positive and significant, however the test of the difference between the two sub‐groups of studies yielded a result of *Q*=.16 with *df*=1 and *p*>.05, meaning that there is not enough evidence that effects differ by presence/absence of prior training.

Fifteen studies that reported *monitoring* the implementation of the programme during the trial yielded a result equal to SMD=.20 (*95%CI* .05 to .35; *p*<.05). In parallel, the 23 studies that did not report monitoring produced an SMD=.37 (*95%CI* .25 to .50; *p*<.001). Both results were positive and statistically significant. The test for differences between sub‐groups showed non‐significant differences (*Q*=2.89; *df*=1; *p*>.05).

#### 4.4.6 Post‐hoc moderators

Based on descriptive analysis of data, and also based on previous research findings (Beelmann & Lösel, 2006; Farrington, Ttofi, & Lösel, 2016), we selected three post‐hoc moderators, namely: i) reasons for conducting the research; ii) evaluator role, and iii) risk of quality bias. Their results are described below:

**Reasons for conducting the research**. We coded data related to the reasons for conducting research. Two categories are explored: i) demonstration study, referring to those studies testing the impact of an intervention under highly controlled optimal conditions and ii) routine evaluation, focusing on testing established programmes under circumstances that approach real‐life conditions (Singal, Higgins, & Waljee, 2014).

Sub‐group analysis produced different effects for the 16 studies conducted for demonstration (SMD=.43; 95% CI .26 t0.59; *p*<.001), and the 18 studies carried out for routine evaluation (SMD= .13; (95% CI .00 to .25; *p*<.05). Demonstration studies reported the highest effect. The between‐studies comparison results were significant (*Q*=8.15; *df*=1; *p*<.05), meaning that the effect varies depending on the reasons for conducting the research.

**Evaluator role.** We coded data identifying the role of the evaluator. Independent evaluators were those not taking part in the design or implementation of the evaluated programme. Dependent evaluators were those also contributing to the design and/or the implementation of the programme.

The 18 trials carried out by independent evaluators produced an SMD= .13 (95% CI .00 to .25; *p*<.05). Unsurprisingly, based on what is known about developer‐led trials, the 16 RCTs conducted by dependent evaluators (i.e., those who also developed and/or designed the intervention) yielded results where SMD=.47 (95% CI .32 to .62; *p*<.001). The between‐groups comparison demonstrated that effect sizes are lower for studies conducted by independent evaluators. These differences were statistically significant (*Q*=12.36; *df*=1; *p*<.001).

**Risk of quality bias.** As reported in section 4.2, we ran EPOC tool for assessing risk of quality bias. Results show that studies vary in the amount of information available for judging their level of risk as well as their risk of bias. We speculated that the quality of studies could explain some of the heterogeneity present in our results. In order to use the results reported by EPOC, we transformed the “low risk”, “unclear risk” and “high risk” into continuous 0, 2 and 3 values respectively. The sum of the results of the eight EPOC criteria resulted in an index that, in our case, was used as a representation of the level of risk of each single study. We tested the role of RoB in meta‐regression.

### 4.5 META‐REGRESSION

In order to explore heterogeneity, we ran meta‐regression using the moderators defined *a priori* (participants’ characteristics and intervention characteristics). Model I included participants’ characteristics only, to get a sense of the net value of these variables in the results. Students’ gender, grade at school or ethnicity (continuous variable), as reported in Table 15, did not explain heterogeneity in the study results. In model II, we introduced the intervention characteristics (i.e., interventions targeting a change at the individual level versus those targeting a change at the school level). Once again, none of the variables present significant results. This suggests that based on the present data, variability across effect sizes cannot be explained by the *a priori* defined modifiers.

**Table 15:** Meta‐regression results: a priori moderators

**Predictors**	***b***	Model I ***SE***	***95%CI***	***b***	Model II ***SE***	***95%CI***
**Intercept** **Participants’ characteristics**	.37	.33	‐.28; 1.06	.37	.34	‐.30;1.04
Gender Male predominant Mixed	.09 ‐.05	.38 .37	‐.64;.82 ‐.77;.67	.11 ‐.06	.39 .38	‐.66; .87 ‐.81; .69
Grade at school (High) Elementary Middle Mixture	‐.11 ‐.22 .17	.21 .20 .40	‐.52;.29 ‐.60;.16 ‐.62;.96	‐.16 ‐.24 .04	.23 .20 .48	‐.61; .29 ‐.64; .16 ‐.90; .98
% of White	‐.00	.004	‐.008; .006	‐.00	.003	‐.00; .00
**Intervention characteristics**						
Individual versus school level change				.11	.21	‐.30; .52

^*^ *p<.05*

We proceed with a meta‐regression model involving post‐hoc variables that could potentially explain the heterogeneity present in our results. Based on the descriptive results in section 4.1.6, and bearing in mind some previous findings, we hypothesise that the characteristics of the research, the ends of the research (demonstration versus routine), the role of the evaluator, and the risk of quality bias could play a role in explaining the results (see Table 16).

**Table 16:** Meta‐regression results: post‐hoc moderators

**Post‐hoc predictors**	**Model**		
** **	***b***	***SE***	***95%CI***
**Intercept** **Characteristics of the research**	*.32*	*.17*	*‐.01;*
The ends of the research (routine vs. demonstration)	.06	.14	‐.20; .33
Role of the evaluator (independent vs. dependent)	‐.36	.14	‐.63; ‐.09^*^
Risk of quality bias	.008	.01	‐.01; .03

* p<.05

The results were significant (*p*<.05) only for the variable role of the evaluator. The coefficient is negative with the category of reference “dependent”, meaning that the effect is lower when an independent team runs research.

**Figure 12:**
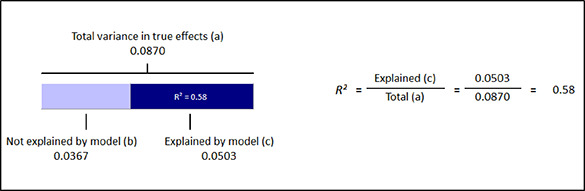
R‐squared graphic. Proportion of the variance explained by the role of the evaluator

Figure 12 presents the value of R‐squared or percentage of explained variance between‐studies based on study‐level characteristics. To compute the total variance (of all studies about the grand mean), we run the regression with no covariates (a). To compute the variance not explained by the model (of all studies about the regression line), we run the regression with covariates (b). Finally, the difference between these values gives us the variance explained by the model which is R^2^=.58, meaning that the model explains 58% of the between‐studies variance.

### 4.6 PUBLICATION BIAS ANALYSIS

Publication bias in systematic reviews occurs when the included set of manuscripts fail to systematically represent the whole population of completed studies that should have been included. The whole population of studies can involve a range of results that must be present in a meta‐analysis to make it valid. However, consistent evidence indicates that studies presenting large effects are more likely to be published than those presenting null or modest effects (Rothstein, Sutton, & Borenstein, 2006). This means that publication bias can lead to an overestimation of the impact of a treatment when running meta‐analysis.

In the present study, much effort was spent in finding most complete collection of published and unpublished studies that test the impact of school‐based intervention on exclusion rates. In fact, almost 50% (see Table 7) of our included studies have never been published in books or peer reviewed journals (i.e., PhD thesis, technical or governmental reports).

**Figure 13:**
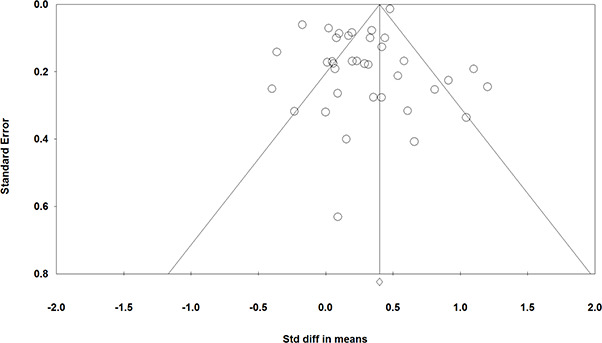
Funnel plot of standard error by standard differences in means

As originally proposed, we use statistical procedures to quantify potential bias that could affect our analysis. First of all, we produce a funnel plot of standard errors by standardised differences in means, presented in Figure 13. In theory, symmetrical distributions of dots under the funnel represent a normal distribution of studies. As anticipated, our studies mostly fall under the funnel, and they are distributed around the main effect. However, since the evaluation of funnel plots can be subjective, we conducted additional statistical measures of publication bias, specifically, Duval and Tweedie's trim‐and‐fill analysis.

Duval and Tweedie's trim‐and‐fill analysis compares the differences in effect sizes that could potentially be attributed to bias. The technique imputes effect sizes until the error distribution gets close to normality. In this way, the test offers the best estimate of the unbiased effect (Borenstein et al., 2009). Results of Duval and Tweedie's trim‐and‐fill analysis suggest that there were no differences in effect sizes attributable to bias. Under the random effect model, the values were SMD=.30; *95%CI* .20 and .41. Based on the parameter of Duval and Tweedie's trim‐and‐fill, it seems that no studies are missing.

### 4.7 SENSITIVITY ANALYSIS

In the present meta‐analysis, the effect of the treatment was calculated as the difference between post‐treatment and baseline. We corrected the value of the variance by assuming a value of correlation equals to .75. In order to test the robustness of this assumption, we ran sensitivity analysis with a correlation equal to .50. Table 17, panel A shows that overall results remain stable when the correlation is smaller.

**Table 17:** Sensitivity analysis

Panel A: with outlier							
**Covariate**	**SMD**	**95% CI**	**SE**	**V**	**n**	**k**	**Measure of Heterogeneity**
0.50	0.31	(.20; .43)	0.058	0.003	36	37	<*Q*=259.0;*df*=36;*p*<.001;*I^2^* =86;*τ^2^* =.081
0.75	0.32	(.21; .44)	0.058	0.003	37	38	<*Q*=338.4;*df*=37;*p*<.001;*I^2^* =89;*τ^2^* =.090
Panel B: without outlier							
0.50	0.27	(.16; .38)	0.056	0.003	36	37	<*Q*=233.3;*df*=36;*p*<.001;*I^2^* =84.5;*τ^2^* =.072
0.75	0.28	(.17; .39)	0.055	0.003	36	37	<*Q*=288.0;*df*=36;*p*<.001; *I^2^* =87.5;*τ^2^* =.076
Panel C: with outlier Winzorised							
0.50	0.30	(.19; .41)	0.057	0.003	37	38	<*Q*=246.23;*df*=37;*p*<.001;*I^2^* =84.9;*τ^2^* =.076
0.75	0.30	(.19; .41)	0.055	0.003	37	38	<*Q*=301.3;*df*=37;*p*<.001;*I^2^* =87.7;*τ^2^* =.078

Another decision was related to the presence of outliers. We found one study presenting an effect size more than three standard deviations from the mean effect size, which was defined as an outlier (Collier, 2002). We tested the impact of the outlier and also the impact of winsorization. The size of the effects, their direction and significance were not altered. See Table 17 below, panels B and C.

Finally, as stated in the protocol, we ran a sensitivity analysis to test differences between published and unpublished reports. The 20 independent effect sizes reported in 19 peer‐reviewed journalsyield a SMD=.31 (95%CI .17 to .45) whereas the 18 effect sizes extracted from unpublished reports reported a SMD=.29 (95%CI .11 to .47). The between effect difference was not statistically significant (*Q*=.028; *df*=1; *p*>.05).

## 5. Discussion

### 5.1 SUMMARY OF MAIN RESULT

In the following paragraphs we present a summary of our results based on the research questions leading this review.

- **Do school‐based programmes reduce the use of exclusionary sanctions in schools?**

The analyses reported in previous chapters suggest that school‐based interventions are capable of producing a small and significant (SMD=.30; *95% CI* .20 to .41; *p*<.001) drop in exclusion rates. It means that those participating in interventions are less likely to be excluded than those allocated to control/placebo groups. These results are based on measures of impact collected on average, six months after treatment. When the impact was tested in the long‐term (i.e., 12 or more months after treatment), the effect of interventions was not sustained. In fact, the impact of school‐based programmes showed a substantial reduction (50%), and was no longer statistically significant.

We ran a further analysis including only the seven studies which reported post‐treatment and follow‐up measures. The overall impact of school‐based interventions at post‐treatment was SMD=.21 (95%CI .11 to .30). Even if the impact was slightly reduced, when we included those seven studies the average time involved under “post treatment” was 12 months on average, meaning that this measure of impact would be more precise and the impact would last longer. Coherent with the original results, the effect produced by combining the seven studies only was null and non‐significant in the long term (i.e., follow‐up measures).

In addition, our results suggest that school‐based interventions present different levels of impact depending on the type of exclusion. After running moderator analysis by type of exclusion, the evidence across studies demonstrated that school‐based interventions are more effective at reducing in‐school than out‐of‐school exclusion. The impact of programmes in the latter case was close to zero with p>.05, meaning that the result could have arisen by chance. Moreover, a larger impact was observed in the sanction expulsion, with a moderate and significant effect size (SMD=.53; 95%CI .07 to .98; p<. 05). However, these results must be cautiously interpreted due to the low number of manuscripts involved in the calculations.

We found 28 studies lacking an operational definition of the dependent variable. The studies did not specify the type of exclusion or exclusion tested, and in that scenario, we decided not to assume this general measure was out‐of‐school exclusion. The impact of school‐based interventions in those “general suspensions” was similar to the overall impact, which probably suggests that these studies are simply a subset of the overall measure.

As originally planned, in this review we ran independent meta‐analysis testing the impact of school‐based interventions ona secondary outcome, that is, internalising and externalising behaviours. It was hypothesised that a reduction in exclusion would be linked with variations in students’ behaviours.

In the case of externalising behaviours, we were not able to calculate a pooled effect size. Results suggest that only five out of the 37 included studies reported a measure for internalising behaviours. However, the narrative description suggests that school‐based intervention had a small effect on the reduction of the above symptoms.

We ran a meta‐analysis based on 15 effect sizes reporting the impact of school based intervention on externalising behaviours. Results show negative and non‐significant impact (SMD=‐ .005; 95%CI ‐.09 to .086; p>.05). It therefore follows that interventions aimed at reducing exclusion do not necessarily reduce antisocial behaviour. This could be in line with evidence suggesting that changes in school policies, rather than changes in behaviour, produce a reduction in disciplinary exclusion (Noltemeyer & Fenning, 2013; Skiba et al., 2015). Two questions arise from these results. First, how do we interpret them, and second, is it still worth implementing programmes that show no impact on antisocial behaviour?

Regarding the first question, one could hypothesise that if race, or more precisely being a racial minority in a given country, is a stronger predictor of school exclusion than other demographic and behavioural characteristics (as shown in Section 1.2), antisocial behaviour would not necessarily explain the rates of exclusion. What is more, it would not be the main cause of this punishment being used. A second possibility is that the behaviour outcome was not measured properly.

The second question considers whether the school‐based interventions tested in this review are still worth implementing if they do not reduce antisocial behaviour. The evidence produced by the present review shows that those programmes presenting positive results do not report negative side effects, in the sense that control groups do not increase the rate of punishment. If that is the case, we suggest that these efforts should be continued. However, based on our data, these ideas cannot yet be regarded as conclusive and more research is needed about the causes that explain the phenomenon of exclusion.

- **Are some school‐based approaches more effective than others in reducing exclusionary sanctions?**

The nine different types of interventions presented in the set of included studies were compared to test the hypothesis that some are more effective than others. There was a considerable variability across the programmes in overall effectiveness. Enhancement of academic skills, mentoring/monitoring, skills training for teachers and counselling/mental health services reported the largest and most significant effect sizes. Based on the number of studies included in each sub‐type, we believe that skills training for teachers and mentoring/monitoring represent the stronger and more reliable findings. The subgroup analysis reported that differences across types of programmes were significant, implying that effects vary depending on the programme implemented.

In line with previous findings, Tolan et al. (2008) examined 39 experimental and quasi‐experimental studies and specifically analysed the effectiveness of mentoring in reducing delinquency, aggression and drug use, and improving academic achievements. The largest positive effects were found for aggression (*SMD* = .40; 95%; *CI* = 0.06 to 0.74) and delinquency (*SMD* = .23; 95% CI .11 to .36). The meta‐analysis conducted by Eby, Allen, Evans, Ng, and Dubois (2008) also found that mentoring programmes had a small and significant positive effect on academic performance.

Overall, mentoring programmes seem to be an effective strategy for reducing violence and conduct problems during adolescence. However, on average the effect sizes reported in relevant studies are small. In this respect, it is important to bear in mind the findings by Tolan et al. (2008). This study suggests that mentoring was more effective when (a) participants had greater pre‐existing behavioural problems or had been exposed to significant levels of environmental risk, (b) they were male, (c) the educational or occupational backgrounds of the mentors fitted the goals of the program, (d) mentors and youths were successfully paired, with similar interests, and (e) programmes were structured to support mentors.

As far as teachers’ skills for managing students’ behaviour is concerned, previous evidence suggests that such programmes canimprove teachers’ general instructional and behavioural management skills in planning, implementing and maintaining effective classroom practices. In fact, a meta‐analysis conducted by Gottfredson, Wilson, and Najaka (2002) found that classroom or instructional management programmes (*k*=25) demonstrated a small and significant effect in reducing antisocial/aggressive behaviour (*SMD*= .13, *p*<.05). The most recent meta‐analysis carried out by Oliver et al. (2011), concluded that classroom management practices (N=12 studies) had a significant, positive effect on reducing problem behaviour. Students taking part in the intervention display less disruptive, inappropriate and aggressive behaviour in the classroom compared to those in control classrooms. The overall effect of the intervention was *g*= .22; p<.05 (Oliver et al., 2011). Evidence also suggests that programmes with the most positive effects tend to be of a longer duration and tend to combine classroom and instructional management strategies with some other major ingredient (e.g. parent training or social skills instruction) (Gottfredson et al., 2002).

The positive relationship between teachers and students has been found to be a factor in promoting more prosocial and less aggressive behaviours later in life. A recent study (Obsuth et al., 2016; p. 16), using a non‐bipartite propensity score matching technique, found that “teachers who reported having a more positive relationship with a student at age ten observed significantly fewer aggressive and defiant behaviours and more prosocial behaviours in the same student concurrently and one year later, at age 11. This was also associated with more prosocial behaviours two years later, at age 12 and also with less aggressive behaviour at age 13. Similarly, students who perceived a more positive relationship with their teacher at age 11 reported fewer aggressive behaviours and more prosocial behaviours concurrently and fewer aggressive behaviours two and four years later, at ages 13 and 15.” All these results make us believe that investing in teachers’ skills and positive relationships between students and teachers is worthwhile, with schools becoming target locations for preventing crime and promoting positive psychosocial development.

Finally, it is worth mentioning that we grouped interventions by those targeting a change at student level versus those expecting a change at school level. Even if the programmes targeting a change at student level display a larger effect size, the between‐group analyses do not allow us to conclude that the differences between these intervention strategies are statistically significant.

- **Do participants’ characteristics (e.g., age, gender, ethnicity) affect the impact of school‐based programmes on exclusionary sanctions in schools?**

Differences in post‐intervention effects were non‐significant when we compared schools whose population was predominantly masculine versus mixed schools. Thus, based on our findings, we could not confirm the hypothesis that the effect differs by the distribution of gender in schools. The same occurred with ethnicity and age. As both variables were continuous, we included them in our meta‐regression. Based on the available data, we could not confirm the hypothesis that the impact of the interventions varies when the intervention was implemented with younger or older students. Similarly, variation in the percentage of white students did not play a role in explaining the heterogeneity of the effect.

- **Do characteristics of the interventions, implementation and methodology affect the impact of school‐based programmes on exclusionary sanctions in schools?**

Twenty‐five studies (reporting 26 independent effect sizes) stated the presence of training hours *before* the intervention was delivered. In the remaining 12 studies, the authors did not mention any kind of training. When comparing the effect of both sets of studies, the between‐group analysis was non‐significant, meaning that there is no evidence that effect differs by presence/absence of training before implementation.

We ran an analysis to investigate the differences between those interventions that deployed monitoring during implementation and those which lacked monitoring. Interventions without monitoring produced significantly larger effects than those with it. In other words, programmes lacking monitoring produced larger effect sizes. This result is in line with previous findings indicating that a lack of monitoring, for instance in routine trials, tends to show smaller effects than demonstration evaluations (Farrington, Ttofi, & Lösel, 2016).

On the other hand, we observed that interventions run by independent evaluators found significantly smaller effect sizes when compared to those studies carried out by researchers involved in the design and/or delivery of the programme. Again, this is not surprising. Previous research has called attention to this phenomenon as well as the advantages of running impendent trials for producing more precise and realistic results (e.g., Eisner, 2009; Manuel Eisner & Humphreys, 2012; Lösel & Beelmann, 2006b; Petrosino & Soydan, 2005; Wilson, Lipsey, & Soydan, 2003).

### 5.2 OVERALL COMPLETENESS AND APPLICABILITY OF EVIDENCE

In the present review we screened a total of 42,749 citations across 27 electronic databases. Pre‐defined searches targeted published and unpublished reports from any country, in any language, as long as the abstract was presented in English. Only one study in a language other than English was identified by our searches. We purposively conducted electronic searches in databases involving manuscripts produced in Latin America and other Spanish and Portuguese‐speaking countries (e.g., SciELO‐Scientific Electronic Library Online). However, we did not find any evaluations whose characteristics make them includable in our review.

When compared to other contemporary reports (e.g., Dymnicki, Weissberg, & Henry, 2011; Mytton et al., 2006), our searches seemto be comprehensive enough to reduce the risk of publications bias. All in all, the extent of our searches and the high percentage of retrieved full reports make us confident that we have identified a substantially complete collection of the available relevant research.

However, this systematic review has allowed us to identify some gaps in the literature. First of all, studies testing the impact of school‐based interventions fail to disentangle the impact on different forms of disciplinary exclusion. In future, the availability of data calculating the impact of interventions across different types of exclusionary punishment could offer more detailed evidence in respect to what works in the field of school discipline.

Secondly, we did not find includable studies testing the impact of restorative justice strategies. One single ongoing study (i.e., Acosta, 2015) was detected by our searches but its results are to be released in 2018. As recently stated by Fronius et al., (2016: 17 and 19), the research evidence to support restorative justice in schools is still in a nascent state. In fact, the scarce evaluations produced so far rest in pre‐post designs lacking control comparison groups, which would likely have a serious impact on the internal validity of the empirical results. The potential for restorative principles to be applied in school settings needs to be explored in more detail.

Thirdly, we believe that the production of independent, high quality evaluations could contribute to more transparent and precise evidence regarding the impact of school‐based programmes. Previous research shows that demonstration programmes or those where designers take part in the evaluation tend to produce larger effect sizes (Beelmann & Lösel, 2006).

Fourth, much of the evidence presented in this review has been produced in the United States where school safety and exclusion is a salient concern for researchers and policy makers alike. As shown in Table 1 of the present report, in a sample of high‐ and middle‐income countries, exclusion is a widespread school punishment. In European countries, exclusion is a more regulated sanction, while in some Latin‐American societies exclusion is under the discretional decision of each particular school (e.g., Chile). We think that research on the use of exclusion in those contexts needs more development.

Fifth, we observe a lack of research testing the long‐term impact of school‐based interventions. The evidence shows that the impact immediately after intervention tends to be larger than that measured several months later (Farrington, Ttofi, & Lösel, 2016). Since in real life policy makers need to invest in effective programmes whose results endure long‐term, we think that an effort should be made to test the impact at least 12 months after intervention ends.

Finally, although we did not include cost‐benefit analysis in our aims, data was coded regarding the presence/absence of interventions’ costs. Two of our included studies reported the economic cost of programme implementation. We believe that cost‐benefit analyses are key for alerting decision makers about the advantages of early intervention. The prevention of exclusion and expulsion from school can reduce future social exclusion, violence and other negative correlates described in the research (Noltemeyer, Ward, & Mcloughlin, 2015).

### 5.3 QUALITY OF THE EVIDENCE

The present review involves only randomised controlled trials. They are considered the best methodological design for isolating confounding factors and producing an accurate measure of intervention effects. However, as stated earlier in the results section, our included reports are lacking a considerable amount of information for judging the quality of the procedures carried out.

More than 50% of the reports fail to provide enough data to judge the precision of randomisation, that is, sequence generation and allocation concealment. Likewise, 30% of the studies did not report data on the equivalence of the groups after randomisation. As recently stated by Roberts & Ker, (2016), missing data on those details can drive analysts to identify “false positive” RCTs. We cannot claim that was the case among our studies, but clearly the absence of detail imposes serious limitations on the assessment of quality in regard to potential threats to internal validity.

A similar lack of data affected the evaluation of blind outcome measures. Even if most of the exclusion measures in our report are based on official records, we cannot ignore the fact that teachers or school staff are in charge of imposing sanctions and could potentially be aware of a student's participation in the experiment. More than 50% of the studies present few details for judging the level of risk involved in this criterion.

Randomised controlled trials of educational programmes are receptive to contamination (Hutchison & Styles, 2010; Simmons et al., 2015). As participants in the control arm may be surrounded by those receiving the treatment, because they share the same school, the likelihood of contamination is a matter of concern. Some of the effects of contamination have been identified by previous research (Giraudeau & Ravaud, 2009; Torgerson, 2001).

Contamination may reduce the effect of the intervention, leading to a type II error – that is, incorrectly retaining a false null hypothesis (a “false negative”) or, described in a different way, a “rejection of an effective intervention as ineffective because the observed effect size was neither statistically nor clinically significant” (Torgerson, 2001). In the present meta‐analysis, 76% of the studies suggest a high risk of contamination bias. It could be the case that our estimation of the impact is underestimated.

In future research, it seems that the use of cluster randomised control trials would help ameliorate these biases. Even if clustered RCTs involve complex designs and demand huge efforts, the quality of the much‐needed evidence can be enhanced by using this methodological design (Hutchison & Styles, 2010). However, the randomisation of clusters implies challenges for researchers. “The main issue is that observations from the same cluster are more similar than observations from two different clusters. This situation requires the use of both an inflated sample size and adapted statistical analysis to take into account this concern” (Giraudeau & Ravaud, 2009, p. 1).

### 5.4 LIMITATIONS AND POTENTIAL BIASES IN THE REVIEW PROCESS

There are several limitations that could affect the results of the present review. It is important to acknowledge that even though we focused on randomised controlled trials, which are supposed to be the best evidence for measuring evaluation impact, included studies present limited information for judging quality bias.

Some 35% of the included studies reported results based on samples with less than 100 participants. The small size of the samples involved in some of the primary research could impose clear limitations on the ability to estimate the effects of interventions.

As reported in the moderator analysis, the independence of the evaluator explained the heterogeneity of effect sizes. Even if independent teams reported on a good number of our studies, close to 50% of them did not use independent evaluators. This fact could add some bias in to our results.

However, it is also important to elucidate some of the advantages of the present study. First of all, this systematic review and meta‐analysis is the first attempt to collect and statistically summarise interventions pursuing a reduction in school exclusion. As such, we believe that our report offers an overview of the amount, characteristics, limitations, and quality of the available evidence, as well as a measure of the size of the effect achieved by different types of intervention. The reader can find in this report an updated review of the evidence produced in the United States and the United Kingdom.

Secondly, we have endeavoured to use an exhaustive coding process to provide careful descriptions of the studies as well as a meticulous analysis of the statistical data available. Due to the inclusion of cluster data, corrections were introduced to make the information comparable at an individual level. Such corrections were carried out using the most recent strategies suggested by Hedges, (2007) and Pigott, (n.d.). We therefore believe that our calculations are at a low risk of underestimating the size of standard errors.

### 5.5 AGREEMENTS AND DISAGREEMENTS WITH OTHER STUDIES OR REVIEWS

The present review is the first meta‐analytical effort to identify the impact of school‐based intervention at reducing school exclusion. In that sense, we have no similar evidence with which to contrast our findings.

However, the results provided by our review seem coherent with other studies testing the impact of school‐based intervention on behavioural outcomes. For instance, Wilson et al., (2011) conducted a meta‐analysis on school‐based programmes looking for a reduction in drop‐outs. Our results more or less target similar programmes and the detected effect size follows a similar trend. In the same vein, Mytton et al., (2006) conducted another review testing the impact of school‐based interventions in preventing violence. They found similar problems with quality assessment and reported an overall effect size slightly better than the one produced by the present review. However, in that last case, the long‐term effects are statistically stronger and last longer. The reduction in aggression reported by Mytton et al., (2006) is not clear in our results.

In a more general examination, a recent review of reviews conducted by Farrington, Ttofi, & Lösel, (2016) found a mean effect^11^ of school‐based programmes equal to SMD=.184 (95%CI .16 to .20). Across the studies, effect sizes ranged between SMD=.091 to SMD=.631. Our findings are, therefore, more or less coherent with the impact of interventions in school settings evaluated by previous meta‐analysis.

## 6. Authors’ conclusions

The empirical evidence produced by this report suggests that non‐punitive school‐based programmes can reduce the use of exclusion. Even if the effects are not sustained for the long term, data shows that in the short term (i.e., six months on average) schools can opt for different and more effective approaches to managing discipline, rather than zero‐tolerance policies. This review, aimed at testing the effectiveness of school‐based programmes, offers a broad overview not only of the effectiveness of different interventions, but also uncovering findings that can guide public policy and future research.

### 6.1 IMPLICATIONS FOR PRACTICE AND POLICY

Research consistently reveals that school exclusion is disproportionately used as a punishment for ethnic minorities, males and those coming from low socio‐economic backgrounds. Exclusion as a disciplinary school measure seems to reduce school attendance, increase drop‐out rates and restrict future possibilities for inclusion in the labour market. Aside from that, research has also been consistent in finding a correlation between permanent exclusion and antisocial behaviour.

Though the causal link between exclusion and the above outcomes is still not clearly established, research based on observational data suggests that the consequences of exclusion affect not only students and their families but extend to the whole society. Indeed, as stated by Rumberger and Losen (2016), exclusion may involve a high economic cost to the taxpayer. In their most recent report, the authors provide a conservative estimate of the economic impact of exclusion. They assert that, in the United States, tenth grade exclusions alone account for more than 67,000 school drop‐outs, at a cost of $35 billion.^12^

Although our results must be cautiously considered, the evidence produced by this review suggests that school administrators and policymakers do have alternatives to exclusion when dealing with disciplinary problems. In our findings, prevention strategies have a small but encouraging impact on exclusion rates, at least in the short term. When comparing different types of programmes, it appears that prevention programmes targeting teachers’ skills as well as those introducing mentoring/monitoring schemes can have a positive impact in reducing exclusion. These results are in line with previous research and also with previous narrative reviews which emphasize the importance of teachers’ skills and mentoring programmes in promoting prosocial behaviours and values (Freiberg & Lapointe, 2006; Gottfredson et al., 2002; Oliver et al., 2011; Tolan et al., 2008).

It is important to clarify that this is most applicable to the United States, where the majority of the assessed evidence was collected. As expressed in the previous paragraphs, some flaws affect the contemporary primary evaluations present in this review. However, these results should encourage researchers to produce better quality evidence rather than abandoning their efforts to find strategies to replace exclusionary punishments.

### 6.2 IMPLICATIONS FOR RESEARCH

Clearly, more primary research evaluating the impact of prevention programmes targeting school discipline is needed. Ideally this research should be conducted under high methodological standards, accounting for mediating mechanisms that lead to a reduction in exclusion rates.

In particular, the results of this first review on the topic identify some implications for future research, laid out below:

- **Addressing the racial gap**. Most of the literature reviewed in the present study indicates that racial or ethnic identity plays a central role in predicting school exclusion, even after controlling for demographic and behavioural variables. More research needs to be developed for testing the mechanisms that produce different treatment for some racial minorities, such as African‐American and Latino students in the United States or Black Caribbean and Gypsy/Romany students in the United Kingdom. It is necessary to understand the individual and social processes that lead to the overrepresentation of minorities in school exclusion. As suggested by some scholars, the divergence between students and teachers’ cultural expectations could potentially contribute to misinterpretation of each other's behaviour, fears and conflicts (Gregory et al., 2010). Understanding the mechanisms that make race a predictor of exclusion could have implications for future policy and practice. For instance, as stated in previous research, greater diversity among staff could be helpful in promoting understanding of cultural differences and reducing bias (Irvin, 2002).
- **Causal effects of exclusion**. The present research has addressed the lack of certainty about the genuine causal effects of exclusion. A review of previous research suggests that exclusion could be a risk factor for a set of negative outcomes already described in the above paragraphs. At the same time, an overview of previous criminological theories suggests that punishment could present different effects on different individuals. The truth is that the causal effect of punishment on students’ behaviour is still a long way from being fully understood. If school exclusion is simply a marker of an underlying antisocial syndrome, it could be beneficial to invest in prevention programmes targeting, for example, deviant behaviours (e.g., violence, drug use, crime, abuse, and neglect) and personality features (e.g., aggressiveness, lack of empathy, lack of remorse) associated with antisocial syndrome (Farrington, 2003). However, if school exclusion is proved to be the *cause* of detrimental outcomes later in life, it will be worth investing in more programmes focusing specifically on the reduction of school exclusion.
- **Mediating mechanisms that explain reductions/increases in exclusion rates.** Future evaluations of school‐based interventions aimed at reducing exclusion need to explore the presumed causal mechanisms that lead to that reduction. More theoretically informed trials that examine causal mechanisms are important to design better interventions. In fact, this information would be crucial in planning future prevention programmes since causal mechanisms can shed light on what works for whom and under what conditions.
- **Recognise the key role of statistical power.** Future research could benefit from prospective power analysis. That means that, during the early stages of the evaluation's design, there must be consideration given to the sample size required to detect an effect. This early effort can protect studies from “underpowered or wastefully overpowered” samples which can affect outcomes by Type I and Type II errors (Ellis, 2010). Future meta‐analysis should also probably give more attention to this issue and, as suggested by Farrington et al., (2016), it seems advantageous to set a minimum sample size for inclusion in reviews.
- **Tackle the challenges of running randomised controlled trials in school settings.** Research conducted within schools often struggles to isolate experimental and control groups, with the subsequent risk of contamination. As stated earlier in this report, the use of cluster randomised controlled trials can present an opportunity for tackling this when conducting research in educational settings.
- **Attempt blind assessment in school‐based randomised controlled trials.** The characteristics of schools make it hard to blind all those involved in trials. Teachers, students and school counsellors are likely to be involved in the experiment, making it difficult to control the social desirability effect of those participating in the study (Hutchison & Styles, 2010). This challenge needs to be addressed by future studies by at least blinding those who collect data. It also seems necessary, at the level of meta‐analytical studies, that the tools used to measure quality bias in school contexts be adapted. In our experience, some of the available “risk of bias tools” seemed more suited for medical trials than for school‐based experiments.
- **Risk of quality bias.** Meta‐analysis integrates the quantitative evidence from different but related studies to summarise a whole body of knowledge on a research question. As in the present review, meta‐analysis can answer the question about the effectiveness of a given intervention. By the fact that meta‐analysis combines results from different primary research reports, quality bias involved in primary research can jeopardise the validity of the “meta” results. In brief, quality bias refers to systematic error, “meaning that multiple replications of the same study will produce different effect estimates because of sampling variation even if they would give the right answer on average” (Higgins & Green, 2011, p.188).
  As stated by Higgins & Green (2011, p. 189) “more rigorous studies are more likely to yield results that are closer to the truth”. Differences in the quality of performed studies can result in false positive conclusions when less rigorous studies are biased toward overestimating an intervention's effect. They can also arrive to false negative conclusions in those cases where less rigorous studies are inclined towards underestimating an effect.
  To evaluate risk of quality bias, the present study involves an evaluation of each of the included reports. Three categories were used to judge a study report; i) low risk; ii) unclear risk and iii) high risk of bias. When it came to randomisation, we found a small percentage of high‐risk cases. However, it is important to note that a large proportion of studies (59%) were categorised as “unclear risk”. This is because many reports presented succinct descriptions of the randomisation process without detailing the methods of sequence generation for instance. When we tested the quality of allocation procedures, once again, we found a high percentage of studies (54%) reporting minimal details of allocation concealment. Similar findings were observed for baseline equivalence (22% of assessed studies reported limited information), attrition (30% unclear risk) and blindness of the outcome assessment (54%). These findings do not necessarily mean that studies present quality bias, they just inform us that the authors omitted data which is relevant to interpret the results. Future research would benefit from following CONSORT standards. Registration of trials, even if they are PhD theses, would represent a huge benefit not only for the scientific community, but also for those interested in evidence‐based decision making.
- **Cross‐cultural research**. Based on our findings, the evidence so far is largely coming from the United States. More research needs to be done in other countries where school exclusion is an issue. We know that evidence suggesting effective approaches in some countries/cultures will not necessarily have the same effectiveness when translated to different populations. Those making decisions about how to reduce exclusion in their own country need to have access to detailed information addressing their particular needs.
- **Innovative strategies.** More research needs to be conducted on innovative strategies, for example those involving empathy‐based philosophies. This was the basis for the intervention tested by Okonofua et al. (2016, p.5521) included in this review. The intervention is focused on encouraging teachers to adopt an empathetic attitude towards discipline; it is low cost and demonstrates long‐term effects (rates from 9.6% to 4.8%). As stated by the authors, “teachers’ mind‐sets about discipline directly affect the quality of teacher‐student relationships and student exclusions and, moreover, can be changed through scalable intervention”.
- **Restorative justice programmes.** In addition to the above, the debate about strategies for reducing exclusion in schools have recently been enriched by some research suggesting that restorative justice programmes could have a promising impact (Anyon, Gregory, Stone, Farrar, & Downing, 2016; Fronius et al., 2016). Scholars have highlighted the advantages of restorative justice over exclusion by showing that these programmes focus on building peaceful and empathetic relationships, ask for the involvement of all parties in achieving conflict resolution, look at the harm done to those affected by misbehaviour, and promote reintegration rather than exclusion (Drewery, 2004; Hostetler, 2014; Varnham, 2005). However, to date, there is no clear evidence about its effectiveness (Fronius et al., 2016). Empirical research needs to be conducted testing the impact of restorative justice in school settings. Two challenges need to be addressed here. First, restorative programmes need to be implemented following the guidelines and characteristics of the restorative processes. This is not a simple task for schools used to dealing with conflict in a punitive way. Secondly, randomised controlled trials or well‐controlled studies need to test the impact of restorative justice practices in dealing with conflict inside schools, such as bullying or other forms of violence (Sherman & Strang, 2007). A potential impact in the reduction of conflict could lead to a reduction in the use of exclusion or other exclusionary strategies.

## 8. Information about this review

### 8.1 REVIEW AUTHORS

**Lead review author:**

The lead author is the person who develops and co‐ordinates the review team, discusses and assigns roles for individual members of the review team, liaises with the editorial base and takes responsibility for the on‐going updates of the review.

**Table:** 

**Name: Sara Valdebenito**
Title: Doctor
Affiliation: Institute of Criminology, University of Cambridge
Address: Institute of Criminology, Sidgwick Avenue.
City, State, Province or County: Cambridge
Postal Code: CB3 9DA
Country: United Kingdom
Phone: +44 1223 767373
Email: sv331@cam.ac.uk; sara.valdebenito@gmail.com
**Co‐authors**:
**Name: Manuel Eisner**
Title: Professor
Affiliation: Institute of Criminology, University of Cambridge
Address: Sidgwick Avenue, Cambridge CB3 9DA
City, State, Province or County: Cambridge
Postal Code: CB3 9DA
Country: United Kingdom
Phone: +44 1223 335374
Email: mpe23@cam.ac.uk
**Name: David P. Farrington**
Title: Doctor
Affiliation: Institute of Criminology, University of Cambridge
Address: Sidgwick Avenue, Cambridge CB3 9DA
City, State, Province or County: Cambridge
Postal Code: CB3 9DA
Country: United Kingdom
Phone:+44 1223 767186
Email: dpf1@cam.ac.uk
**Name: Maria M. Ttofi**
Title: Doctor
Affiliation: Institute of Criminology, University of Cambridge
Address: Sidgwick Avenue, Cambridge CB3 9DA
City, State, Province or County: Cambridge
Postal Code: CB3 9DA
Country: United Kingdom
Phone: +44 1223 767186
Email: mt394@cam.ac.uk
**Name: Alex Sutherland**
Title: Doctor
Affiliation: RAND
Address: Westbrook Centre, Milton Road
City, State, Province or County: Cambridge
Postal Code: CB4 1YG
Country: United Kingdom
Phone: +44 1223 273 884
Email: asutherl@rand.org

### 8.2 ROLES AND RESPONSIBILITIES

Please give brief description of content and methodological expertise within the review team. The recommended optimal review team composition includes at least one person on the review team who has content expertise, at least one person who has methodological expertise and at least one person who has statistical expertise. It is also recommended to have one person with information retrieval expertise.

Who is responsible for the below areas? Please list their names:

- Information retrieval and coding: Mr Aiden Cope and Dr Sara Valdebenito M.
- Risk of Bias assessment: Dr Alex Sutherland and Dr Sara Valdebenito M.
- Advise in statistical methods and contents: Professor Manuel Eisner, Professor David P. Farrington, Dr Alex Sutherland and Dr Maria M. Ttofi
- Statistical analysis and report writing: Dr Sara Valdebenito M.

### 8.3 ACKNOWLEDGEMENT

We are very grateful of Aiden Cope for his assistance with information retrieval and double coding.

### 8.4 SOURCES OF SUPPORT

Professor Manuel Eisner and Dr Sara Valdebenito have been awarded a grant by the Nuffield Foundation for conducting the proposed systematic review. Terms and conditions agreed with the sponsor involve the submission of results during 2016.

### 8.5 DECLARATIONS OF INTEREST

None of the researchers involved in the team present financial interest in this review. None of them have been involved in the development of interventions or systematic reviews on the scope of the present one. Three authors (Dr Sara Valdebenito M, Professor Manuel Eisner and Dr Alex Sutherland) were involved in the London Education and Inclusion Project cluster‐randomised controlled trial (ISRCTN 23244695). The study was designed as an independent evaluation and the authors have no financial or other links to the evaluated programme.

### 8.6 PLANS FOR UPDATING THE REVIEW

Dr Sara Valdebenito will be responsible for updating the present review every three years.

### 8.7 AUTHOR DECLARATION

**Authors’ responsibilities**

By completing this form, you accept responsibility for maintaining the review in light of new evidence, comments and criticisms, and other developments, and updating the review at least once every five years, or, if requested, transferring responsibility for maintaining the review to others as agreed with the Coordinating Group. If an update is not submitted according to agreed plans, or if we are unable to contact you for an extended period, the relevant Coordinating Group has the right to propose the update to alternative authors.

**Publication in the Campbell Library**

The Campbell Collaboration places no restrictions on publication of the findings of a Campbell systematic review in a more abbreviated form as a journal article either before or after the publication of the monograph version in *Campbell Systematic Reviews*. Some journals, however, have restrictions that preclude publication of findings that have been, or will be, reported elsewhere, and authors considering publication in such a journal should be aware of possible conflict with publication of the monograph version in *Campbell Systematic Reviews*. Publication in a journal after publication or in press status in *Campbell Systematic Reviews* should acknowledge the Campbell version and include a citation to it. Note that systematic reviews published in *Campbell Systematic Reviews* and co‐registered with the Cochrane Collaboration may have additional requirements or restrictions for co‐publication. Review authors accept responsibility for meeting any co‐publication requirements.

**I understand the commitment required to update a Campbell review, and agree to publish in the Campbell Library. Signed on behalf of the authors**:

**Form completed by:**

**Dr Sara Valdebenito**

**Date:**

**25 September 2017**

## 9. Tables

### 9.1 CHARACTERISTICS OF INCLUDED STUDIES

The following tables offer a succinct characterisation of each included study. We selected 11 characteristics from our coding which will allow the reader an overview of the study.

1. Methodological design, clarifying the type of randomised controlled trial (e.g., cluster randomised, matched pairs randomisation).
2. Characteristics of the participants, involving the size of the sample during randomisation, school grade of participants, percentage of males and predominant ethnicity.
3. Location of the study, detailing the city and country where the study was implemented.
4. Brief description of the intervention.
5. Programme deliverers, those in charge of implementing the programme.
6. Evaluator role. When the evaluator is only in charge of conducting the trial, we define that as “an independent evaluator.” Ifin addition the researcher delivers or designs the programme, we report that additional role.
7. Outcomes measured in the study, not only the outcomes we are interested in.
8. Length of the intervention.
9. Assessment. This data refers to the measures carried out during the evaluation; for instance, baseline and post treatment, or baseline, post treatment and 12 month follow up.
10. Attrition. This data provides an estimate of the percentage of reported attrition.
11. Conflict of Interest Statement (COI). Offers information on the absence/presence of a formal and explicit statement declaring conflict of personal or institutional interest.
12. Potential Conflict of Financial Interest (CoFI)

1) Allen, Philliber, Herrling, & Kuperminc (1997)

**Table:** 

**Methods**	Randomised controlled trial
**Participants**	695 ninth through twelfth grade school students Treatment: 14% Male, 68% Black Control: 17% Male, 67% Black
**Location**	United States (nationwide)
**Interventions**	Teen Outreach Program. It is a volunteer service programme, designed to prevent teen pregnancy and academic failure by enhancing normative processes of social development. The intervention involves supervised volunteer community service, classroom‐based discussion of service experience and classroom‐based discussions related to social development tasks of adolescence.
**Programme deliverers**	Teachers
**Evaluator role**	Unclear
**Outcomes measured**	School suspension Teen pregnancy Course failure
**Length of intervention**	One academic year (around 35 weeks)
**Assessment**	Baseline and post treatment
**Attrition**	Treatment group=5.3% Control group= 8.4%
**COI statement**	Not presented
**CoFI**	Unclear

2) Arter (2005)

**Table:** 

**Methods**	Randomised controlled trial
**Participants**	52 sixth grade school students 100% Male, 72.5% African‐American
**Location**	Maryland, US
**Interventions**	Positive Alternative Learning Support (PALS). The programme aims to make the student more competent in the school environment by providing integrated behavioural support, academic support, group counselling and mentoring.
**Programme deliverers**	School administrator, school psychologist, counsellors, teachers, special education teachers, one volunteer parent and the researcher
**Evaluator Role**	Delivered the programme
**Outcomes measured**	Attendance Academic achievement Office referrals Suspension from school
**Length of the intervention**	18 weeks
**Assessment**	Baseline and post treatment (immediately after intervention)
**Attrition**	24%
**COI statement**	Not presented
**CoFI**	Possible. The author delivered the intervention although the programme does not appear to be commercially available (p. 39)

3) Barnes, Bauza, & Treiber (2003)

**Table:** 

**Methods**	Randomised controlled trial
**Participants**	45 high school students 71% Male, 100% African American
**Location**	Richmond County, United States
**Interventions**	Stress reduction via Transcendental Meditation.
**Programme deliverers**	Certified instructor
**Evaluator role**	Independent
**Outcomes**	Tardy periods Absentee periods Grades Rule infractions Days suspended
**Length of intervention**	12 weeks
**Assessment**	Baseline and during treatment
**Attrition**	9%
**COI statement**	Declared
**CoFI**	Unlikely, independent evaluator.

4) Berlanga (2004)

**Table:** 

**Methods**	Randomised controlled trial
**Participants**	80 eighth grade school students Gender is not clearly reported (mixed). 80% Hispanic
**Location**	South Texas, United States
**Interventions**	Grades, Attendance and Behaviour (GAB). In this programme, a cognitive‐behavioural classroom guidance curriculum is combined with supportive and individualized solution‐focused counselling sessions (six sessions).
**Programme deliverer**	School counsellor
**Evaluator role**	Independent
**Outcomes**	Self‐esteem Self‐conception Academic achievements Attendance Office referrals Suspensions
**Length**	12 weeks
**Assessment**	Baseline and post treatment
**Attrition**	21%
**COI statement**	Not presented
**CoFI**	Unlikely, independent evaluator (p. 135‐36)

5) Bradshaw, Waasdorp, & Leaf (2012)

**Table:** 

**Methods**	Clustered randomised controlled trial
**Participants**	12,334 students nested in 37 elementary schools 52% Male, 46.1% White (largest ethnic group)
**Location**	Maryland, United States
**Interventions**	School‐Wide Positive Behavioural Interventions and Supports (SWPBIS): a programme that targets a systemic change process in a whole school or in a district. SWPBIS aims to reduce students’ behaviour problems by changing staff behaviours and developing systems and supports to meet children's behavioural needs.
**Programme deliverers**	School staff, teachers and administrators
**Evaluator role**	Independent
**Outcomes**	Aggressive and disruptive behaviour Concentration problems Pro‐social behaviour Emotion regulation Office disciplinary referrals Out‐of‐school suspension
**Length**	Four years of implementation
**Assessment**	Five times over the course of four years.
**Attrition**	“Participation rate was consistently high, […] no evidence that missing data was problematic” p. e1140
**COI statement**	Declared
**CoFI**	Unlikely, independent evaluator (p. e1136)

6) Bragdon (2010)

**Table:** 

**Methods**	Randomised controlled trial
**Participants**	68 eighth‐grade students 61% Male; 53% African‐American
**Location**	United States
**Interventions**	Teach team project. The intervention is a multi‐component drop‐out prevention involving career exploration, a career awareness course and daily check‐in/check‐out monitoring by a school counsellor or a mentor teacher.
**Programme deliverers**	Counsellor and mentor teachers
**Evaluator role**	Independent
**Outcomes**	Academic performance in Mathematics and Language Attendance Disciplinary referrals Suspensions
**Length**	Nine weeks
**Assessment**	Baseline, post treatment and follow‐up
**Attrition**	Results suggest no attrition
**COI statement**	Not presented
**CoFI**	Possible. Manuscript suggests that the researcher adapted an intervention based on previous evidence (p. 7‐15).

7) Brett (1993)

**Table:** 

**Methods**	Randomised controlled trial (randomisation of classrooms N=6)
**Participants**	126 seventh grade students. Mixed gender (percentages not given), 100% African‐American
**Location**	District of Columbia, United States
**Interventions**	Efficacy, DC is a mentoring programme aimed at motivating and assisting students to excel academically and socially.
**Programme deliverers**	Eight adult volunteer mentors
**Evaluator role**	Independent
**Outcomes**	Absenteeism School suspension Self‐esteem Academic achievements Attitudes towards learning
**Duration**	12 weeks
**Assessment**	Baseline and post‐treatment (immediately after the intervention)
**Attrition**	23%
**COI statement**	Not presented
**CoFI**	Unlikely. The study author is not the programme developer, collaborator with the programme developer or licence holder (p.110)

8) Burcham (2002)

**Table:** 

**Methods**	Randomised controlled trial
**Participants**	71 seventh and eighth grade pupils in two schools 56% Male, ethnicity not reported
**Location**	Kentucky, United States
**Interventions**	Social Problem Solving Skills Training. Framed in a cognitive behavioural model, the intervention “taught children problem solving skills using modelling, guided practice or applied practice”. It is a manualised intervention of 27 lessons.
**Programme deliverer**	Two school psychologists, one of them the evaluator
**Evaluator role**	Delivered the programme
**Outcomes**	Social and academic competency Self‐concept In‐school and out‐of‐school suspension Disciplinary referrals School tardies
**Length of intervention**	30 weeks
**Assessment**	Baseline, post treatment (immediately after intervention) and follow‐up (18 months later)
**Attrition**	One students missed at post test 43% at follow up
**COI statement**	Not presented
**CoFI**	Possible. Researcher seems to be involved in programme development (p. 42) and also delivered part of the intervention (p.53‐4)

9) Collier (2002)

**Table:** 

**Methods**	Randomised controlled trial
**Participants**	60 elementary school students 100% Male, 100% African‐American
**Location**	Washington, US
**Interventions**	Pro‐social skills training. Skills involve anger management, the development of interpersonal skills and problem solving techniques.
**Programme deliverer**	External psychologist
**Evaluator role**	Delivered the programme
**Outcomes**	Oppositional behaviour Cognitive problems Hyperactivity ADHD School suspension
**Length**	Eight weeks
**Assessment**	Baseline and post treatment
**Attrition**	15% (51 remain)
**COI statement**	Not presented
**CoFI**	Possible. Author delivered and tested the intervention (p.69). Programme seems not to be commercially available.

10) Cook et al. (2014)

**Table:** 

**Methods**	Randomised controlled trial
**Participants**	106 ninth to tenth grade school students 100% Male, 95% Black
**Location**	Chicago, US
**Interventions**	Two interventions to be tested. Becoming a Man (BAM) is a non‐academic intervention, which exposes youth to pro‐social adults and provides social‐cognitive skill training. The Match Model is an academic intervention, providing intensive individualised instruction by tutors.
**Programme deliverer**	College educated individuals
**Evaluator role**	Unclear. “Intervention was delivered by staff hired by our own research team” (p.11)
**Outcomes**	Maths achievements Reading achievements Discipline incidents Days absent Out‐of‐school suspension Participation in extra‐curricular activities
**Length**	BAM: 27 weeks during one academic year Match‐Model: daily, one academic year
**Assessment**	Baseline and post treatment
**Attrition**	Author recognise attrition. Use of Multiple Imputation and ITT analysis
**COI statement**	Not presented
**CoFI**	Unclear

11) Cornell, Allen, & Fan (2012)

**Table:** 

**Methods**	Randomised controlled trial
**Participants**	201 school students (K‐12) 73% boys, 73% African‐American
**Location**	Virginia, US
**Intervention**	Virginia Student Threat Assessment Guidelines is a problem‐solving approach to violence prevention aimed at resolving conflict and working out a solution that allows the student to continue in school.
**Programme deliverer**	School administrator, a law enforcement officer or school resource officer, and one or more mental health professionals
**Evaluator role**	Designed the programme
**Outcomes**	Long‐term school suspension Access to counselling Alternative school placement Parent conference Victim's parents notified
**Length**	Not detailed
**Assessment**	Baseline and post treatment
**Attrition**	Results suggest no attrition
**COI statement**	Declared. Main author designed the Virginia student threat assessment guidelines.
**CoFI**	Possible. See p.100, footnote.

12) Crowder (2001)

**Table:** 

**Methods**	Randomised controlled trial
**Participants**	109 seventh grade school students 45% Male, 99% Black
**Location**	United States
**Interventions**	Gang resistance, education and training (GREAT). Intervention involves leadership skills development and a character education curriculum. The curriculum involved drug resistance, gang resistance, and non‐violent confrontational skills among others.
**Programme deliverer**	Police Officer, assisted by two teachers
**Evaluator role**	Independent evaluator
**Outcomes**	Out‐of‐school suspension In‐school suspension Office referrals Tardies Unexcused absences Students achieving honour roll
**Length**	Nine weeks
**Assessment**	Baseline and post treatment
**Attrition**	Results suggest no attrition
**COI statement**	Not presented
**CoFI**	Unlikely. Researcher was a PhD student who asked permission to run an evaluation implemented by the Metropolitan Police Service (see appendix B).

13) Dynarski et al. (2003)

**Table:** 

**Methods**	Randomised controlled trial
**Participants**	968 elementary school students 46.4% Male, 67% Black
**Location**	United States
**Interventions**	21^st^ Century Community Learning is an after‐school programme delivered in a public school building providing academic support and recreational/cultural activities.
**Programme deliverer**	Teachers
**Evaluator role**	Independent
**Outcomes**	Achievements Levels of effort School suspension Absenteeism Tardies Parental supervision After school activities
**Length**	One academic year
**Assessment**	Baseline, post treatment and one‐year follow‐up
**Attrition**	11%
**COI statement**	Not declared
**CoFI**	Unlikely. See Dynarski et al., 2004, p. XV

14) Edmunds et al. (2012)

**Table:** 

**Methods**	Randomised controlled trial
**Participants**	1607 ninth grade school students 41.4% Male, 60.2% White
**Location**	North Carolina, US
**Interventions**	Early College High School targets students underrepresented in college (i.e., low income, the first in their family to go to college or members of a minority group). It is a personalised programme designed to boost the academic progress of students, minimising barriers between high school and college.
**Program deliverers**	Teachers, school staff.
**Evaluator role**	Independent
**Outcomes**	Course taking patterns and success Suspension Absences Planning to attend four‐year college Algebra I College prep. maths courses English
**Length**	Not clearly stated
**Assessment**	Baseline and post treatment. Unclear how many months or years after intervention.
**Attrition**	4.8%
**COI statement**	Not presented
**CoFI**	Unlikely.

15) Farrell, Meyer, & White (2001)

**Table:** 

**Methods**	Clustered randomised controlled trial
**Participants**	626 sixth grade school students, nested in 27 classrooms 50.2% Male, 96% Black
**Location**	Richmond, Virginia, US
**Interventions**	Responding in Peaceful and Positive Ways (RIPP) is a universal violence prevention program. “The goal of RIPP is to increase adolescents’ capacity and motivation to respond to developmental challenges in ways that facilitate social skill acquisition and acceptance of personal responsibility” (p.452).
**Programme deliverers**	Prevention specialists
**Evaluator role**	Designed the programme
**Outcomes**	Disciplinary violations for violence In‐school suspensions Out‐of‐school suspensions Violent behaviour frequency Drug use frequency RIPP knowledge test Problem situation inventory Attitudes toward nonviolent behaviour Attitudes toward violent behaviour
**Length**	25 weeks
**Assessment**	Baseline, post‐treatment, six‐month follow up and 12‐months follow‐up.
**Attrition**	Yes. Attrition affected both groups. ITT analysis.
**COI statement**	Not declared
**CoFI**	Possible. See p. 452

16) Feindler, Marriott, & Iwata (1984)

**Table:** 

**Methods**	Randomised controlled trial
**Participants**	36 junior high school students Gender and ethnicity not reported
**Location**	Not reported
**Interventions**	Anger Control Training is based on a cognitive behavioural model. Training sessions focus on the components of the provocation cycle, self‐monitoring skills, self‐control, problem solving, time‐out responses, relaxation techniques, and appropriate verbal and non‐verbal assertive responses.
**Programme deliverers**	Trained therapist and programme aide
**Evaluator role**	Unclear
**Outcomes**	Problem solving Self‐control School expulsion
**Length**	Seven weeks
**Assessment**	Baseline, during treatment and five‐week follow‐up
**Attrition**	Results suggest no attrition
**COI statement**	Not declared
**CoFI**	Unclear

17) Harding (2011)

**Table:** 

**Methods**	Randomised controlled trial
**Participants**	48 eighth grade school students 60.4% Male, 97.9% White
**Location**	East England, United Kingdom.
**Interventions**	Over to You is a programme based in a cognitive behavioural approach. It encourages behavioural change as well as offering training in social skills enhancement (e.g., self‐awareness, decision‐making, empathy, conflict resolution).
**Programme deliverer**	Educational psychologist, trained member of the school staff
**Evaluator role**	Delivered the programme
**Outcomes**	Emotional literacy Behaviour (SDQ) School exclusion
**Length**	Six weeks
**Assessment**	Six‐months follow‐up
**Attrition**	8%
**COI statement**	Not declared
**CoFI**	Possible. The researcher implemented the intervention (p. 97‐107)

18) Hawkins, Doueck, & Lishner (1988)

**Table:** 

**Methods**	Randomised controlled trial
**Participants**	160 seventh grade school students (low achievers) 47% Male, ethnicity not reported.
**Location**	Seattle, US.
**Interventions**	Proactive Classroom management can be defined as a package of instructional methods (interactive teaching and co‐operative learning) in mainstream schools. Improved instruction is aimed at benefitting low achievers by improving behaviour, attitudes and academic results.
**Programme deliverer**	Teachers
**Evaluators role**	Independent
**Outcomes**	Students social bonding to school Students achievements Expectations and aspirations for education Antisocial behaviour Suspension and expulsion from school
**Length**	One academic year
**Assessment**	Baseline and post treatment
**Attrition**	11%
**COI Statement**	Not declared
**CoFI**	Unlikely

19) Hirsch, Hedges, Stawicki, & Mekinda (2011)

**Table:** 

**Methods**	Randomised controlled trial
**Participants**	535 High school students 41% Male, 76% African‐American
**Location**	Chicago, United States.
**Interventions**	After School Matters (ASM) is a programme offering paid apprenticeship‐type experiences. Instructors provide information, guidance and feedback, and introduce students to the standards, language and culture of that line of work. The experience helps students begin to appreciate and adapt to the culture of the workplace and improve the “soft skills” increasingly demanded by employers. Intervention takes place in the host high school.
**Programme deliverer**	Instructor and school members
**Evaluator role**	Independent
**Outcomes**	Positive youth development Marketable job skills Academic outcomes Problem behaviour School suspension
**Length**	20 weeks
**Assessment**	Baseline and post treatment
**Attrition**	18%
**COI statement**	Not declared
**CoFI**	Unlikely. See acknowledgments section.

20) Hostetler & Fisher (1997)

**Table:** 

**Methods**	Randomised controlled trial
**Participants**	317 fourth grade school students and their families. 57.4% Male, 33.8% Caucasian
**Location**	Lancaster, US.
**Interventions**	Project CARE, a substance abuse prevention program, aimed at improving students’ skills (problem solving, peer resistance) and parents’ skills.
**Programme deliverer**	Psychologist and prevention specialist
**Evaluator role**	Independent
**Outcomes**	Negative behaviours Intent to use substances Alternative activities Communication with parents Substance use School absences, school suspensions, school grades
**Length**	One school year plus a summer, one session per week
**Assessment**	Baseline, post treatment, 12‐months follow‐up, 21‐months follow‐up
**Attrition**	40% (12 months follow‐up) 75% (21‐months follow‐up)
**COI statement**	Not declared
**CoFI**	Unlikely

21) Ialongo, Poduska, Werthamer, & Kellam (2001)

**Table:** 

**Methods**	Clustered randomised controlled trial
**Participants**	678 elementary school students, nested in 27 classrooms (nine schools) 53% Male, 86.8% African‐American
**Location**	Baltimore, US.
**Interventions**	1) Classroom centred intervention (CC) is aimed at improving classroom management. It involves three main components: curriculum enhancement, enhanced behaviour management practices and back‐up strategies for children not performing adequately. 2) Family School Partnership (FSP) is anintervention targeting improvement in parent‐teacher communication and parents’ child behaviour management strategies.
**Programme deliverers**	CC is delivered by teachers FSP is delivered by teachers plus school psychologist or school social worker
**Evaluator role**	Designed the programme
**Outcomes**	Conduct problems in school School suspension Academic achievements Mental health needs
**Assessment**	Baseline and five‐year follow‐up. There was a post treatment measure but school suspension was not measured.
**Attrition**	24%
**COI statement**	Not declared
**CoFI**	Possible. Researcher seems to be the programme designer (p.602)

22) Johnson (1983)

**Table:** 

**Methods**	Randomised controlled trial
**Participants**	60 seventh and eighth grade school students Gender and ethnicity not reported
**Location**	Washington, US
**Interventions**	Project A.T.T.E.N.D. (Alternatives To Trouble Encouraging New Directions). An education programme targeting self‐responsibility for maintaining school discipline. An alternative to punitive disciplinary methods that combines supervision of attendance, behaviour and classwork as well as counselling.
**Program deliverer**	School counsellor
**Evaluator role**	Design and delivery
**Outcomes**	Attendance (absences and tardies) Suspension Number of misbehaviour referrals Grades
**Length**	Nine weeks
**Assessment**	Baseline and post treatment
**Attrition**	No attrition reported
**COI statement**	Not declared
**CoFI**	Possible. Researcher is programme developer and deliverer (p.7)

23) Lewis et al. (2013)

**Table:** 

**Methods**	A matched‐pair, clustered randomised controlled trial
**Participants**	624 elementary school students, nested in 14 schools 47% Male, 48% African‐American
**Location**	Chicago, United States
**Interventions**	Positive Action programme includes a K‐12 classroom curriculum involving six components: self‐concept, social and emotional positive actions for managing oneself responsibly, positive actions directed toward physical and mental health, honesty, getting along with others, and continually improving oneself. The programme also includes teacher, counsellor, family, and community training as well as activities directed toward school‐wide climate development.
**Programme deliverer**	School facilitators
**Evaluator role**	Design the programme
**Outcomes**	Support of aggression Bullying Disruptive behaviours Violence Disciplinary referrals Suspension
**Length**	One year
**Assessment**	Baseline (2004), post treatment (2005) plus multiple follow‐ups.
**Attrition**	5% post test
**COI statement**	Declared
**CoFI**	Likely. “The research described herein was done using the program, the training, and technical support of Positive Action, Inc., in which Dr. Flay's spouse holds a substantial financial interest. Issues regarding conflict of interest were reported to the relevant institutions and appropriately managed following the institutional guidelines.” (p.629)

24) Mack (2001)

**Table:** 

**Methods**	Randomised controlled trial
**Participants**	20 fourth to six grade school students 50% Male, 100% African‐American
**Location**	Alabama, US
**Interventions**	ICAN Kids! Control Anger Now and Skills for Living curriculum. Abehavioural group counselling intervention programme involving techniques such as behaviour contracts, relaxation, role‐play and modelling.
**Programme deliverer**	Counsellor
**Evaluator role**	Design and delivery
**Outcomes**	Disciplinary referrals School Suspension
**Length**	Six weeks
**Assessment**	Baseline, during treatment, post treatment and three weeks follow‐up.
**Attrition**	No attrition reported
**COI statement**	Not presented
**CoFI**	Possible. See p. 110

25) Obsuth et al. (2016)

**Table:** 

**Methods**	Clustered randomised controlled trial (minimisation)
**Participants**	738 secondary school students, nested in 36 schools 71% Male, 40.3% Black‐Caribbean/Black‐African
**Location**	London, United Kingdom
**Interventions**	Engage in Education – London (EiE‐L). The intervention targeted their social communication and broader social skills with the aim of reducing school exclusions and problem behaviours.
**Programme deliverer**	Core worker trained to deliver the intervention
**Evaluator role**	Independent
**Outcomes**	School exclusion Interpersonal communication Pro‐social skills Student‐teacher relationship Antisocial behaviour Bullying perpetration Delinquency Arrests
**Length**	12 weeks
**Assessment**	Baseline and post treatment
**Attrition**	12% (based on official records)
**COI statement**	Declared
**CoFI**	Unlikely. Independent evaluation.

26) Okonofua, Pauneskua, & Walton (2016)

**Table:** 

**Methods**	Clustered randomised controlled trial
**Participants**	1682 middle school students, 31 teachers 48% Male, 54% Latino
**Location**	California, United States
**Interventions**	Empathic Discipline is a brief intervention (i.e., online training, low cost) aimed at encouraging teachers to adopt an empathic mind‐set about school discipline. The intervention encourages teachers to understand the values and perspectives of students which can cause misbehaviour. Teachers are empowered to handle difficult interactions, in a context of mutual understanding and trust. Empathic discipline is seen as an alternative to punitive, zero‐tolerance practices.
**Programme deliverer**	Teachers
**Evaluator role**	Unclear
**Outcomes**	School suspension Respect to teachers
**Length**	One year
**Assessment**	Baseline and post treatment
**Attrition**	13%
**COI statement**	Declared
**CoFI**	Unclear

27) Panayiotopoulos & Kerfoot (2004)

**Table:** 

**Methods**	Randomised controlled trial
**Participants**	124 secondary school students 90% Male. Most of the students were White British. No percentage reported.
**Location**	Manchester, United Kingdom
**Interventions**	Home and School Support Project (HASSP)is delivered by a multidisciplinary team offering rapid assessment and a treatment plan for the child, family and school staff. Treatment involves family therapy, individual CBT therapy and school support.
**Programme deliverer**	Social worker, psychologist, community psychiatric nurse, play therapist
**Evaluator role**	Independent
**Outcomes**	School exclusion Disruptive antisocial behaviour and emotional symptoms
**Length**	Unclear
**Assessment**	Baseline, three months post‐treatment
**Attrition**	6%
**COI statement**	Not declared
**CoFI**	Unlikely. Intervention was delivered by community services (p.110)

28) Peck (2006)

**Table:** 

**Methods**	Randomised controlled trial
**Participants**	1050 fifth through eighth grade school students 59% Male, 62.5% Hispanic
**Location**	United States
**Interventions**	Student Targeted with Opportunities for Prevention (STOP program). It is a crime prevention programme offering a tutorial component, family and individual counselling, gang education as well as a drug and alcohol counselling intervention. The aim of the programme is that students will enter high school having what it takes to be a successful student and community member.
**Programme deliverers**	Probation Officer, community agencies
**Evaluator role**	Independent
**Outcomes**	School grades School attendance School suspension School expulsion Alcohol and drug use Contacts with the Juvenile Justice System
**Length**	One year
**Assessment**	Follow‐up
**Attrition**	39%
**COI statement**	Not presented
**CoFI**	Unlikely. Programme was implemented by the probation service in collaboration with public agencies (p. 41‐3)

29) Reese, Murphy, & Filipczak (1981)

**Table:** 

**Methods**	Randomised controlled trial
**Participants**	98 seventh through ninth grade school students Gender is not reported, 100% Black
**Location**	United States
**Interventions**	Preparation through Responsive Education Programs (PREP). The intervention is designed to improve academic performance and social skills. Intervention involves reinforcement, and teaching adaptive behaviour as well as skills for self‐control.
**Program deliverer**	Teachers
**Evaluator role**	Unclear
**Outcomes**	School grades School attendance School suspension School behaviour
**Length**	27 weeks
**Assessment**	Baseline, during treatment
**Attrition**	Unknown
**COI statement**	Not presented
**CoFI**	Unlikely

30) Russell (2007)

**Table:** 

**Methods**	Randomised controlled trial
**Participants**	61 sixth grade school students 63.5% Male, 83% Caucasian
**Location**	Oregon, US
**Interventions**	Abbreviated version of Coping Power (CP)attempts to improve a child's social competence, self‐regulation, self‐control and social bonds with peers, teachers and caregivers. CP incorporates individual counselling sessions, weekly group meetings and monthly parent meetings.
**Programme deliverer**	Psychologists, master level clinicians
**Evaluator role**	Independent evaluator
**Outcomes**	Adaptive functioning School referrals Detentions Suspensions
**Length**	24 weeks
**Assessment**	Post treatment
**Attrition**	14%
**COI statement**	Not presented
**CoFI**	Unlikely. Researcher is not programme developer, there is no evidence that the programme is commercialised.

31) Shetgiri, Kataoka, Lin, & Flores (2011)

**Table:** 

**Methods**	Randomised controlled trial
**Participants**	108 ninth grade school students Experimental group: 51% Male, 81% Latino Control group: 33% Male, 75% Latino
**Location**	California, United States
**Interventions**	School‐based programme to reduce violence and substance use. Originally designed for White and African‐American youths, the intervention is group based and aimed at increasing resilience through skills enhancement sessions (e.g., anger management, conflict resolution) and counselling sessions.
**Programme deliverer**	School clinical social worker
**Evaluator role**	Independent
**Outcomes**	Smoking Alcohol and drug use Fighting Grades Suspension Expulsion
**Length**	28 weeks
**Assessment**	Baseline and eight month follow‐up
**Attrition**	20%
**COI statement**	Not presented
**CoFI**	Unlikely

32) Smith (2004)

**Table:** 

**Methods**	Randomised controlled trial
**Participants**	40 eleventh to twelfth grade school students (Hispanic‐serving schools) 60% Male, 65% Hispanic‐Latino
**Location**	Texas, United States
**Interventions**	The Personal Responsibility Group consists of instructional activity sessions on Emotional Intelligence skills (e.g., assertiveness, comfort and rapport, empathy, time‐management, anxiety reduction, and motivation). The aim of the intervention is to assist students in identifying EI strength and areas of growth and positive change.
**Programme deliverer**	Unknown
**Evaluator role**	Delivered the programme
**Outcomes**	Personal responsibility Constructive thinking Expectative Grades School behaviour In‐school and out‐of‐school suspension
**Length**	10 weeks
**Assessment**	Baseline and post treatment
**Attrition**	No attrition
**COI statement**	Not presented
**CoFI**	Possible. Researcher delivered the intervention (p. 285)

33) Snyder et al. (2010)

**Table:** 

**Methods**	Cluster randomised controlled trial
**Participants**	544 students nested in 20 elementary schools Gender is not reported, 30% Part Hawai'i an
**Location**	Three Hawaiian Islands, US.
**Interventions**	Positive Action programme includes a K‐12 classroom curriculum involving six components: self‐concept, social and emotional positive actions for managing oneself responsibly, positive actions directed toward physical and mental health, honesty, getting along with others, and continually improving oneself. The programme also includes teacher, counsellor, family, and community training as well as activities directed toward school‐wide climate development.
**Programme Deliverer**	Teachers, school administrators, school staff, counsellors
**Evaluator role**	Designed the programme
**Outcomes**	Absenteeism Suspension Retention in grades School achievements
**Length**	35 weeks
**Assessment**	Baseline, post treatment, one year follow‐up
**Attrition**	Unclear
**COI statement**	Declared
**CoFI**	Likely. “The research described herein was done using the program and the training and technical support of Positive Action, Inc. Dr. Flay's spouse holds a significant financial interest in Positive Action, Inc.” (p.50)

34) Sprague, Biglan, Rusby, Gau, & Vincent (2016)

**Table:** 

**Methods**	Cluster randomised controlled trial
**Participants**	13,498 nested in 35 middle schools No data on gender; ≈70% White
**Location**	Oregon, US.
**Interventions**	School‐Wide Positive Behavioural Interventions and Supports (SWPBIS) refers to a programme that targets a systemic change process in a whole school or in a district. SWPBIS aims to reduce students’ behaviour problems by changing staff behaviours and developing systems and supports to meet children's behavioural needs.
**Programme Deliverer**	School staff, teachers and administrators
**Evaluator role**	Independent
**Outcomes**	In‐school suspension, out‐of‐school suspension and expulsion School level achievements Antisocial behaviour and aggression Safety Substance use Relationship with teacher Attachment to school rules
**Length**	One year
**Assessment**	Baseline, post treatment and 12‐month follow‐up
**Attrition**	Unclear
**COI statement**	Not declared
**CoFI**	Unlikely

35) Tilghman, (1988)

**Table:** 

**Methods**	Randomised controlled trial
**Participants**	100 sixth to eighth grade school students Gender and ethnicity are not reported
**Location**	United States
**Interventions**	Counsellor Peers are “students who have been properly trained through a certified peer counselling programme coordinator to work with and listen their peers. Rather than being an “advice‐giver” or “problem‐solver”, peer counsellors are sensitive listeners trained in communication skills to help their peers through the process of decision making and self‐exploration.” (p.10)
**Programme deliverer**	Trained students
**Evaluator role**	Evaluator trained the counsellor peers
**Outcomes**	Attitude towards school, themselves and peers Suspension
**Length**	Nine weeks
**Assessment**	Unclear
**Attrition**	9%
**COI statement**	Not presented
**CoFI**	Possible. Researcher was involved in the selection and training of counsellors. It is regarded as a limitation in the study (p. 9). No evidence that the programme is commercialised.

36) Ward & Gersten, (2013)

**Table:** 

**Methods**	Cluster randomised controlled trial
**Participants**	32 elementary schools; 7500 students Gender is not reported. Ethnic minorities represent ≈80%
**Location**	United States
**Interventions**	Safe and Civil Schools (SCS) is a school‐wide intervention promoting positive behaviour and support. “Program deliverers receive training on how to implement improvements related to safety, behaviour and discipline”. (p.320)
**Programme deliverer**	School administrator, three general education teachers, one special education teacher and one or two other staff.
**Evaluator role**	Independent
**Outcomes**	Suspension Achievements test scores Resiliency, protective factors, and risk behaviours Opinion on the level of implementation of the school‐wide intervention
**Length**	One year
**Assessment**	Post treatment and one‐year follow‐up
**Attrition**	Unclear
**COI statement**	Not presented
**CoFI**	Unlikely. Programme was developed by Sprick et al.1992 for profit but evaluator seems to be independent.

37) Wyman et al. (2010)

**Table:** 

**Methods**	Cluster randomised controlled trial (randomised within classrooms)
**Participants**	226 kindergarten to third grade school students 54% Male, more than 60% Black
**Location**	Rochester City, US
**Interventions**	Rochester Resilience Program: “In 14 lessons with school‐based mentors, children were taught a hierarchical set of skills: monitoring of emotions; self‐control/reducing escalation of emotions; and maintaining control and regaining equilibrium. Mentors provided classroom reinforcement of skill use”. (p. 707)
**Program deliverer**	Resilience Mentors
**Evaluator role**	Design the programme
**Outcomes**	Classroom behaviour Social‐emotional functioning Office disciplinary referrals Out‐of‐school suspension
**Length**	14 weeks
**Assessment**	Baseline and post treatment
**Attrition**	13%
**COI statement**	Not presented
**CoFI**	Possible. Main author developed the programme (p.709). No information about commercialisation of the programme.

### 9.2 RISK OF BIAS IN INCLUDED STUDIES BASED ON EPOC RISK OF BIAS TOOL (PER STUDY)

**Table:** 

**Study**	**Sequence generation**	**Concealment**	**Equivalence (outcome)**	**Equivalence (Characteristics)**	**Incomplete data**	**Blinding**	**Contamination**	**Selective outcome reporting**
Allen et al. 1997	HR	UR	HR	HR	LR	HR	HR	LR
Arter, 2005	UR	UR	UR	HR	UR	UR	HR	LR
Barnes et al. 2003	HR	UR	HR	LR	HR	UR	LR	LR
Berlanga, 2004	UR	UR	HR	HR	UR	HR	HR	LR
Bradshaw et al. 2012	LR	LR	LR	LR	LR	UR	LR	LR
Bragdon, 2010	UR	UR	HR	HR	UR	UR	HR	LR
Brett, 1993	UR	LR	UR	HR	HR	UR	HR	LR
Burcham, 2002	LR	LR	LR	UR	HR	UR	HR	LR
Cook et al. 2014	LR	LR	LR	LR	LR	UR	HR	LR
Collier, 2002	UR	UR	LR	LR	LR	HR	HR	LR
Cornell et al. 2012	UR	UR	LR	LR	LR	UR	LR	HR
Crowder, 2001	UR	HR	UR	UR	LR	UR	HR	LR
Dynarski et al. 2003	UR	LR	LR	LR	UR	UR	HR	LR
Edmunds et al. 2012	HR	UR	UR	LR	UR	UR	HR	LR
Farrell et al. 2001	LR	UR	HR	LR	LR	LR	HR	LR
Feindler et al. 1984	UR	UR	LR	HR	LR	LR	HR	LR
Harding, 2011	UR	UR	UR	HR	HR	HR	HR	LR
Hawkins et al. 1988	UR	UR	LR	LR	UR	HR	HR	LR
Hirsch et al. 2011	LR	LR	LR	LR	LR	UR	HR	LR
Hostetler & Fisher 1997	UR	UR	UR	HR	HR	HR	HR	LR
Ialongo et al. 2001	UR	UR	LR	LR	UR	HR	HR	LR
Johnson, 1983	LR	LR	LR	LR	LR	HR	HR	LR
Lewis et al. 2013	LR	LR	UR	LR	LR	HR	LR	LR

### 9.6 EXTRACTED DATA FOR EFFECT SIZE CALCULATIONS: PRIMARYOUTCOME

**Table:** 

**Author**	**Type of Study**	**Sample**	**Mean** ^35^ **Age** **(SD)**	**School‐based Programme**	**Universal/ Indicated** ^36^	**% FSM**	**Cluster** ^37^	**Extracted data for** **effect size calculations**	**Effect size calculation**	**Measure of exclusion**
1) Allen et al. (1997)	Journal article	695	15.8 (1.13)	Teen outreach	Unclear	Unknown	No	**Suspension** **Baseline** T1: 58 cases (17%); N=342 C1: 81 cases (23.8%); N=353 **Post treatment (immediately after treatment)** T2: 42 cases (13%); N=324 C2: 93 cases (28.7%); N=323	Effect size was calculated as the difference between baseline and post treatment. We corrected final calculations by adding the value of pre/post correlation, assumed to be equal .75. See methods section for further details	Presence/absence
2) Arter, (2005)	Journal article	52	Secondary school	Positive Alternative Learning Support (PALS)	Indicated	40% FSM	No	**Suspensions No baseline reported Post Treatment (presumably after treatment)** T2: M=.675; SD=.194; N=23 C2: M= 675; SD=.227; N=17	SMD was calculated using equations 3 and 4 in the methods section.	Nº of days
3) Barnes et al. (2003)	Journal article	45	16 (1.3)	Stress reduction	Universal	Unknown	No	**Suspension** **Baseline** T1: M=0.8 days; SD=1.8; N=23 C1: M=0.0 days; SD=.0; N=18 **During intervention** T2(during): M= 0.5 days; SD=1.2; N=23 C2(during): M=1.2 days; SD=3.0 N=18	The principal investigator provided N size for T2. SMD was calculated as the difference between time 1 and time 2, accounting for the covariation between pre‐ and post measures (equations 8 and 9 in the methods section).	Nº of days
4) Berlanga (2004)	PhD Thesis	80	Eighth grade	Grades, Attendance and Behaviour (GAB)	Indicated	Unknown	No	**In School Suspension** **Baseline** T1: M=1.15; SD=1.29; N=32 C1: M=.61; SD=1.14; N=31 **Post Intervention (immediately after treatment)** T2: M=1.12; SD=1.21; N=32 C2: M=1.03; SD=1.88; N=31 **Suspension** **Baseline** T1: M=.31; SD=.53; N=32 C1: M=.35; SD=.83; N=31 **Post Intervention(immediately after treatment)** T2: M=.34; SD=.90; N=32 C2: M=.58; SD=1.11; N=31 **Removal/Expulsion** **Baseline** T1: M=.06; SD=.24; N=32 C1: M=.06; SD=.24; N=31 **Post Intervention (immediately after treatment)** T2: M=.03; SD=.17; N=32 C2: M=.16; SD=.45; N=31	Effect size was calculated as the difference between baseline and post treatment. We corrected final calculations by adding the value of pre/post correlation, assumed to be equal. 75. See methods section for further details	Nº of events
5) Bradshaw et al. (2012)	Journal article	12,334	Elementary school	School‐Wide Positive Behavioural Interventions and Support (SWPBIS)	Universal	49% FSM	Yes	**Out‐of‐school suspension Follow‐up (four years)** **Student level** Control: 21 schools (N=5124) Treatment: 16 schools (N=6614)OR= .73; 95% CI .59 and .91	The study reports results using multi‐level analysis. In this case, we have not applied any correction of standard errors. We assume that MLM accounted for clusters and subsequently corrected the bias (see p. e1140). See methods section for further details	Nº of events
6) Bragdon (2010)	PhD Thesis	68		Teach Team Project	Indicated	49% FSM	No	**Suspension** **Baseline** T1:M=.06; SD=.34; N= 34 C1:M=.96; SD=2.42; N=34 **Post treatment** (during) T2:M=.03; SD=.17; N= 34 C2:M=1.10; SD=2.61; N=34 **Follow‐up (three months later)** T3:M=.09; SD=.51; N=34 C3:M=1.07; SD=3.19; N=34	Effect size was calculated as the difference between baseline and post treatment. We corrected final calculations by adding the value of pre/post correlation, assumed to be equal. 75. See methods section for further details.	Nº of days
7) Brett (1993)	PhD Thesis	126	12‐14 years	Efficacy, DC	Universal	Unknown	Yes	**School Suspension** **Clusters: 3 control (n=66) 3 Experimental (n=60)** **Post‐treatment (1 Month)** T2: M=.53; SD= 1.02; N=40 C2: M=.63; SD=1.21; N=57;	Based on Hedges (2007) and Spier et al. (2013), effect sizes were computed using *d_T2_*, assuming equal cluster sample size, ρ=.05. See methods section for further details	Nº of events
8) Burcham (2002)	PhD Thesis	71	Middle school	Social problem solving skills training	Indicated	38% FSM	No	**In‐School Suspension‐Baseline** T1:M=8.62; SD=6.44; N=37 C1:M=7.88; SD=4.47; N=32 p=.58 **‐Immediately after treatment** T2: f=.18; p= 0.67; N=69 **‐18 months after treatment** T3: f=.04; p=0.84; N=38 **Out‐of‐school Suspension Baseline** T1: M=3.22; SD=3.71; N=37 C1: M=2.56; SD=3.40; N=32 p=.45 **Immediately after treatment** T2: f=1.09; p=.30; N=69 **18 months after treatment** T3: f= 1.83; p=.18; N=38	Effect size was calculated as the difference between baseline and post treatment. We corrected final calculations by adding the value of pre/post correlation, assumed to be equal. 75. See methods section for further details	Nº of events
9) Collier (2002)	PhD Thesis	60	5‐14 years	Pro‐social skills training	Indicated	Unknown	No	**School Suspension (+)** **Baseline** T1: M=1.93; SD=.4498; N=26 C1: M=1.86; SD=.5074; N=25 **Post treatment (presumably after treatment)** T2:M=1.15; SD=.6748; N=26 C2:M=2.16; SD=.3742; N=25	Study was identified as an outlier value. It was winsorised as suggested by Wilson & Lipsey 2001. Effect size was calculated as the difference between baseline and post treatment. We corrected final calculation by adding the value of pre/post correlation, assumed to be equal .75.	Nº of days
10) Cook et al. (2014)	Technical report	106		BAM (skills‐training) and MATCH (tutoring)	Indicated	26% FSM	No	**Baseline data is reported but incomplete** **Out‐of‐school suspension** (ITT) *b*=‐.642; SE=.501; (unclear the number of months/weeks of post treatment measured)	Data was entered into CMA by using the option Log OR and its SE. No further corrections. Even if the evaluation was testing two different interventions, data was reported in a composite measure. The original author took that option because they recognised contamination between groups (spill‐over).	Nº of events
11) Cornell et al. (2012)	Journal article	201		Threat assessment	Indicated	Unknown	Yes	**Long‐term suspension** **Post treatment (presumably after treatment)** T2: 25 (25%); N=100 C2: 49 (49%); N=101	No corrections	Nº of events
12) Crowder (2001)	PhD Thesis	109		Gang Resistance, Education and Training (GREAT)	Unclear	Unknown	No	**Out of School Suspension No baseline measure reported Post intervention** (**presumably after treatment)** T2: M=.1329; SD=.4629; N=53 C2: M=.1429; SD=.1610; N=56 **In School Suspension No baseline measure reported Post intervention (presumably immediately after treatment)** T2: M=.3584; SD=.7464; N=53 C2: M=.4464; SD=.8464; N=56	SMD was calculated using equations 3 and 4 in the methods section. No further corrections.	Nº of events
13) Dynarski et al. (2003:2004)	Technical report	968	Elementary school	21^st^ Century Community Learning	Unclear	Unknown	No	**Post treatment 12 months (2003)** T2: 7.1% (38) N=537 C2: 5.2% (16) N=317 **Follow up 24 months** T3: 60 (6.2%) N=537 C3: 43 (4.4%) N=317	Data was entered into CMA by using a 2x2 table. No further corrections.	Nº students
14) Edmunds et al. (2012)	Journal article	1607	15.3	Early College High School Academic skills enhancing	Unclear	50.6%	No?	**% Suspended at least once** T2: 6.4%; (57) N=885 C2: 13.3%; (86) N=644	The principal investigator provided measures for effect size calculation (via mail communication).	Nº of events
15) Farrell et al. (2001)	Journal article	626	11.7 (0.6)	Responding in Peaceful and Positive Ways (RIPP)	Universal	Unknown	Yes	**In‐school suspension** Post intervention **(immediately after treatment)** OR=5.0 (95%CI 1.5; 17.1) **6 months** OR=1.4 (95% CI .7; 2.8) **12 months** OR=1.4 (95%CI .6; 3.0) **Out‐of‐School Suspension** Post‐intervention (**immediately after treatment)** OR=0.9 (95%CI .5; 1.8) **6 months** OR=1.1 (95% CI .6; 2.0 **12 months** OR=0.9 (95% CI .6; 1.4)	Although the study is based on clustered data, we have not applied any correction of standard errors. The author mentions the use of GEE to calculate robust estimates of standard errors (see Farrell et al., 2001, p. 455). See methods section for further details.	Nº of events
16) Feindler et al. (1984)	Journal article	36	13.8 (.68)	Anger control training	Indicated	Unknown	No	**School Expulsion** **Baseline** T1: M=1.45; SD=.71; N=18 C1: M=1.40; SD=.44; N=18 **Five‐weeks follow‐up** T2: M=.77; SD=.29; N=18 C2: M=.1.2; SD=.46; N=18	Effect size was calculated as the difference between baseline and post treatment. We corrected final calculation by adding the value of pre/post correlation assumed to be equal .75. See methods section for further details.	Nº of events
17) Harding (2011)	PhD Thesis	48	Eighth grade	Over to you	Indicated	Unknown	No	**Six‐months follow‐up** **Fixed term exclusion** T2: 5 N=20 C2: 5 N=23 **Permanent exclusion** T2: 1 N=20 C2: 1 N=23	Data was entered into CMA by using a 2x2 table. No further corrections.	Nº of events
18) Hawkins et al. (1988)	Journal article	160	Seventh grade	Proactive Classroom Management	Indicated	Unknown	No	**Times Suspended** **Post‐treatment (presumably after treatment)** T2: M=.48; SD=1.3; N=67 C2: M=.89; SD=2.1; N=75	SMD was calculated using equations 3 and 4 in the methods section. No further corrections.	Nº of events
19) Hirsch et al, (2011)	Technical report	535	15.9	After School Matters	Indicated	86%	No	**School Suspension** **Baseline** T1: M=1.27; SD=.63; N=259 C1: M=1.29; SD=.64; N=178 **Post treatment (immediately after treatment)** T2: M=1.36; SD=.72; N=259 C2: M=1.40; SD=.75; N=178	Effect size was calculated as the difference between baseline and post treatment. We corrected final calculations by adding the value of pre/post correlation, assumed to be equal .75. See methods section for further details.	Nº of events
20) Hostetler & Fisher (1997)	Journal article	317	Third grade	Project CARE (Skill for parents and children)	Indicated	Unknown	No	**Suspension** **Baseline** T1: M=0.13; SD=0.56; N=151 C1: M=0.07; SD=0.35; N=140 **Post treatment** (a few months after treatment, no clear specification) T2: M=0.20; SD=0.53; N=155 C2: M=0.25; SD=0.89 N=141 **One year follow‐up** T3: M=0.26; SD=0.80; N=90 C3: M=0.15; SD=0.66, N=86 **Two year follow‐up** T4: M=0.27; SD=0.74; N=30 C4: M=0.09; SD=0.29; N=34	Effect size was calculated as the difference between baseline and post treatment. We corrected final calculations by adding the value of pre/post correlation, assumed to be equal .75. See methods section for further details.	Nº of events
21) Ialongo et al. (2001)	Journal article	678	6.20 (.34)	Two interventions i) Classroom‐centred (CC) ii) Family‐school partnership (FSP)	Universal	62.3% FSM	Yes	**Suspension** **Five year follow‐up** **Classroom‐centred vs. control** OR=.73; (95%CI=.56; .95) ** treatment group less likely to be suspended **Family‐school partnership** OR=.59 (95%CI .35; .97) Boys: OR=1.13 (95%CI .61; 2.09) Girls: OR=.38 (95%CI .17; .86) ** treatment group less likely to be suspended	Since data was dichotomous and nested in clusters, we corrected standard errors of effect sizes. The design effect was corrected by using the formula suggested by Higgins & Green (2011) expressed by the equation [1+(M‐1) x1]. See methods section for further details.	Presence/absence
22) Johnson (1983)	PhD Thesis	60	Seventh and eighth grade	ATTEND (Counselling and monitoring)	Indicated	Unknown	No	**Suspension Baseline** T1: M=.76; SD=.85; N=30 C1: M=.83; SD=.87; N=30 **Post intervention** (after treatment) T2: M=.36; SD=.55; N=30 C2: M=1.5; SD=1.25 N=30	Effect size was calculated as the difference between baseline and post treatment. We corrected final calculations by adding the value of pre/post correlation, assumed to be equal .75.	Nº of events
23) Lewis et al. (2013)	Journal article	624	Elementary school	Positive Action	Universal	Grade 3 84% FSM	Yes	**Suspension** **Baseline (2001)** T1: M=40.95; SD=48.13; N=3648 C1: M=65.25; SD=56.15; N=3800 **Post treatment (2004)** T2: M=55.17; SD=64.84; N=3407 C2: M=77.63; SD=66.8; N=3687 **Follow‐up (2005)** T3: M=68.08; SD=80.02; N=3367 C3: M=88.96; SD=76.56; N=3539	The principal investigator provided data for calculations. Based on Hedges (2007) and Spier et al. (2013), effect sizes were computed using *d_T2_*, assuming equal cluster sample size, ρ=.05.. See methods section for further details.	Nº of events
24) Mack (2001)	PhD Thesis	20	Fourth to sixth grade	ICAN Kids! Behavioural group counselling	Indicated	95% (for school, no stated for sample)	No	**Out‐of‐school Suspension** **Baseline** T1: M=1.5000; SD=.9718; N=10 C1: M=1.9000; SD=.8756; N=10 **3 weeks (during)** Tduring: M=.8000; SD=.6325; N=10 Cduring: M=.9000; SD=.7379; N=10 **Post‐treatment** T2: M=.3000; SD=.4830; N=10 C2: M=.4000; SD=.5164; N=10 **+ 3 weeks** T3: M=.0000; SD=.0000; N=10 C3: M=1.0000; SD=.6667; N=10	Effect size was calculated as the difference between baseline and post treatment. We corrected final calculations by adding the value of pre/post correlation assumed to be equal .75. See methods section for further details.	Nº of events
25) Obsuth et al. (2016)	Journal article	738	13.9	Engage in Education (Skills training)	Indicated	32%	Yes	**Exclusion** Official Records Baseline T1: OR=2.784; SE=.300; p=.001 Post‐treatment (1 month) T2: OR=1.444; SE=.389; p=.344	The study reports results using multi‐level analysis. In this case, we have not applied any correction of standard errors. We assume that MLM accounted for clusters and subsequently corrected bias (see p. 11). The study offered measures of impact based on self‐reporting, teachers’ reports and official records. We extracted from OR based on official records (most of our studies report official records of suspension).	Presence/absence
26) Okonofua et al. (2016)	Journal article	1682	Middle school	Empathic Discipline	Universal	Unknown	Yes	**Suspension** **Post treatment (unclear number of months/weeks)** T2: OR=.42; z= ‐3.33; p=.001; N=1449 31 clusters	Since data was dichotomous and nested in clusters, we corrected standard errors of effect sizes. The design effect was corrected by using the formula suggested by Higgins & Green (2011) expressed by the equation [1+(M‐1) x1]. See methods section for further details. Published data did not provide confidence intervals or SE. We tried to contact authors but it was not possible. We calculate an approximate SE=.013.	Presence/absence
27) Panayiotopoulos & Kerfoot (2004)	Journal article	124	10	Home and School Support Project (HASSP)	Indicated	Unknown	No	**Exclusion** T1: M=9.50; SD=14.81; N=62 C1: M=5.11; SD=7.56; N= 62 **Post treatment (After three months)** T2: M=4.95; SD=13.11; N=61 C2: M=5.51; SD=11.94; N=62	Effect size was calculated as the difference between baseline and post treatment. We corrected final calculations by adding the value of pre/post correlation, assumed to be equal .75.	Nº days
28) Peck (2006)	PhD Thesis	1050	Fifth to eighth grade	Student Targeted with Opportunities for Prevention (STOP)	Unclear	Unknown	No	**Suspension** **Post‐treatment (unclear number of weeks/months after treatment)** T2: 22; N=315 C2: 22; N=321	Data was entered into CMA by using a 2x2 table. No further corrections.	Nº of events
29) Reese et al. (1981)	Journal article	98	Seventh to ninth grade	Preparation through Responsive Education Programs (PREP)	Indicated	Unknown	Matched peers	**Suspension** **During school year** T2 vs C2: X^2^ (1)= 6.58, p<.02	Data was entered into CMA by using *X^2^* originally reported. No further corrections.	Nº of days
30) Russell (2007)	PhD Thesis	61	11.5 (.46)	Coping Power (Skills training for reducing aggression)	Indicated?	Unknown	No	**Suspension** **Post treatment** T2(School A): M=.15; SD=.38; N=13; C2(School B): M=.31; SD=.60; N=16; T2(School B: M=.29; SD=.61; N=14 C2(School B):M=.00; SD=.00;N=10	SMD was calculated using equations 3 and 4 in the methods section. No further corrections.	Nº of events
31) Shetguiri et al. (2011)	Journal article	108	14	Violence and drug use reduction	Indicated	100%	No	**Suspended or Expelled** **Baseline** T1: 8 (21%) N=40 C1: 10 (22%) N=46 **Eight months follow‐up** T2: 6 (14%) N=40 C2: 4 (8%) N=46	Effect size was calculated as the difference between baseline and post treatment. We corrected final calculations by adding the value of pre/post correlation, assumed to be equal .75.	Presence/absence
32) Smith (2004)	PhD Thesis	40		The Personal Responsibility Group (Emotional Intelligence skills)	Indicated	Unknown	No	**In‐school Suspension** **Post‐treatment** T2: f=11.085; p greater than or equal to .002; **Out‐of‐school Suspension** **Post treatment** T2: f= 10.088; p greater than or equal to .003	SMD was calculated based on f‐test. No further corrections.	Nº of events
33) Snyder et al. (2010)	Journal article	544	Elementary school children	Positive Action	Universal	55%	Yes	**Suspension (% of students suspended) 2002** T1: M=1.12; SD=1.10; N=5000 C1: M=.98; SD=1.11; N=5000 **2006** T2: M=.67; SD=.64; N=5000 C2: M=1.72; SD=1.55; N=5000 **2007** T3: M=.84; SD=.61; N=5000 C3: M=2.53; SD=2.80; N=5000	The principal investigator provided sample sizes for calculations. Based on Hedges (2007) and Spier et al. (2013), effect sizes were computed using *d_T2_*, assuming equal cluster sample size. Since the report presented the value of ρ, we used this value in calculations.	Nº of students
34) Sprague et al. (2016)	Unpublished paper	13,498	Middle school children	School‐Wide Positive Behavioural Interventions and Support (SWPBIS)	Universal	Unknown	Yes	**Expulsion** **Baseline** T1: M=.002; SD=.004; N=6492 C1: M=.003;SD=.004; N=7006 **Post treatment** T2: M=.002; SD=.004; N=6492 C2: M=.003; SD=.005; N=7006 **Follow‐up (1 year later)** T3: M=.003; SD=.006; N=6492 C3: M=.003; SD=.004; N=7006 **In School Suspension** **Baseline** T1: M=.071; SD=.094; N=6492 C1: M=.135;SD=.189; N=7006 **Post treatment** T2: M=.064; SD=.087; N=6492 C2: M=.097; SD=.133; N=7006 **Follow‐up (1 year later)** T3: M=.058; SD=.060; N=6492 C3: M=.095; SD=.145; N=7006 **Out‐of‐School Suspension** **Baseline** T1: M=.082; SD=.063; N=6492 C1: M=.078; SD=.065; N=7006 **Post treatment** T2: M=.076; SD=.077; N=6492 C2: M=.061; SD=.042; N=7006 **Follow‐up (1 year later)** T3: M=.073; SD=.064; N=6492 C3: M=.075; SD=.051; N=7006	Principal investigator provided data for calculations. Based on Hedges (2007) and Spier et al. (2013), effect sizes were computed using *d_T2_*, assuming equal cluster sample size, ρ=.05. See methods section for further details.	Nº of events
35) Tilghman (1988)	PhD Thesis	100	12.5	Counsellor Peers	Indicated?	Unknown	No	**Suspension** T2: 11 (N=46) C2: 26 (N=45) Significance test	Data was entered into CMA by using a 2x2 table. No further corrections.	Nº of students
36) Ward & Gersten (2013)	Journal article	33 schools ≈ 25,000 students record	Elementary school children	Safe and Civil Schools	Universal	90%	Yes	**Post treatment (end of intervention)** OR=.83; SE=.05 **Follow‐up (1 year later)** **(cumulative impact)** OR=.77; SE=.04	Since data was nested in clusters, we corrected standard errors of effect sizes. The design effect was corrected by using the formula suggested by Higgins & Green (2011) expressed by the equation [1+(M‐1)x1].	Nº of days
37) Wyman et al. (2010)	Journal article	226	K ‐ 3rd	Rochester Resilience Programme	Indicated	90%	Yes	**Suspension events** **Post‐treatment (immediately after intervention)** TC2: Exp b=−0.57; SE=0.23; z=−2.48; p=0.013 Controlling for suspension T1 59 classrooms, 4 students per classroom	Since data was nested in clusters, we corrected standard errors of effect sizes. The design effect was corrected by using the formula suggested by Higgins & Green (2011), expressed by the equation [1+(M‐1) x1]. See methods section for further details	Nº of events

***Abbreviations*** *: T1(treatment group baseline measure); C1 (control group baseline measure); T2 (treatment group post treatment measure); C2 (control group post treatment measure); T3(treatment group follow up measure); C3(control group follow up measure); FSM (free school meals); M (mean); SD (standard deviation); N (sample size); OR (Odds ratio); 95% CI (95% confidence interval); SE (standard error); f (ANCOVA coefficient); p (p‐value); b (beta coefficient); X^2^ (chi‐squared)*.

35 When the mean age was not available in the original study, their grade in school has been reported. Their school grade gives the reader a general idea of the age of the students.

36 Universal intervention strategies are those oriented to reach the entire population of students, without regard to individual risk factors. Indicated programmes were defined as those targeting students displaying behavioural problems, punished at school or presenting a specific risk to their educational development.

37 Due to the nature of the settings (schools) some studies reported clustered data. We corrected SE errors when it was needed. See methods section for further details.

## Figures

### 10.1 PERCENTAGE OF REPORTS WITHIN YEAR PERIOD

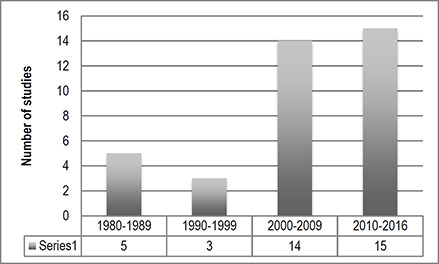

## 11. Data and analyses

### 11.1 SUB‐GROUP ANALYSES

#### Forest plot sub‐group analysis: Predominant gender in school

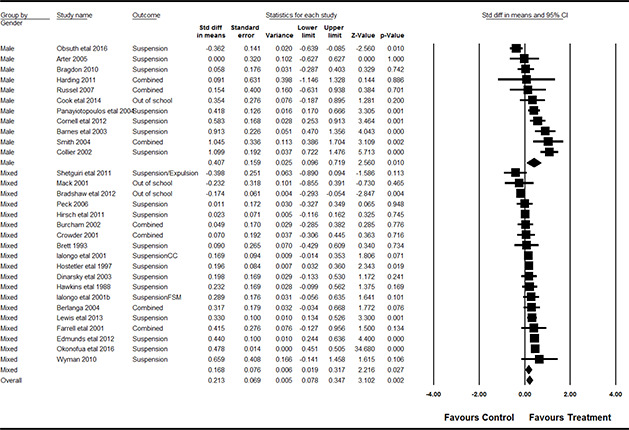

#### Forest plot sub‐group analysis: Grade at school

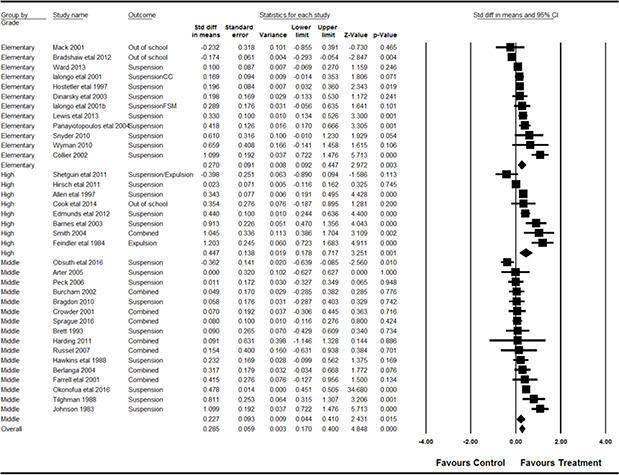

#### Forest plot sub‐group analysis: Type of intervention

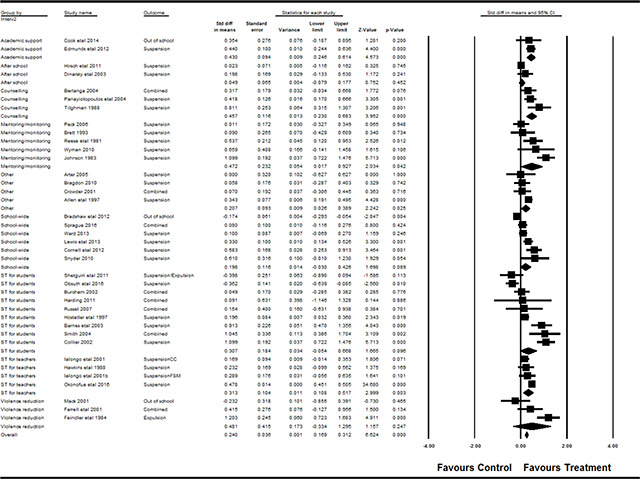

#### Forest plot sub‐group analysis: Theoretical bases of interventions

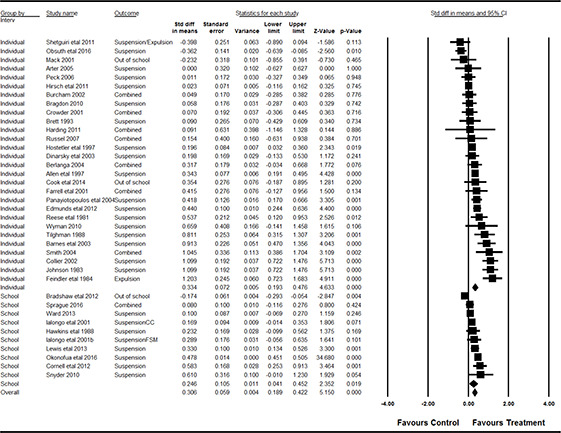

#### Forest plot sub‐group analysis: Training before intervention

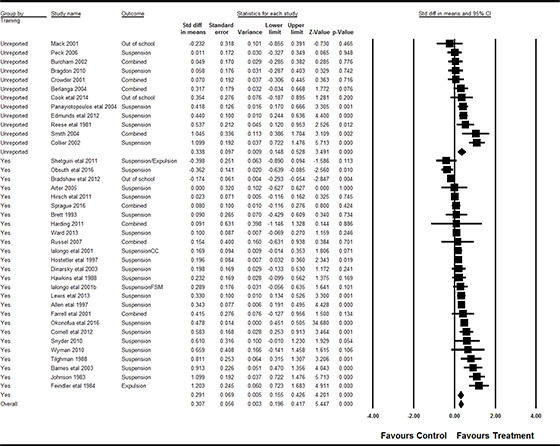

#### Forest plot sub‐group analysis: Monitoring the implementation of the intervention

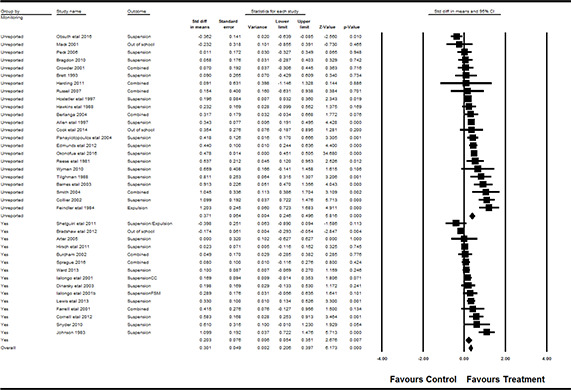

#### Forest plot sub‐group analysis: Reasons for conducting the research

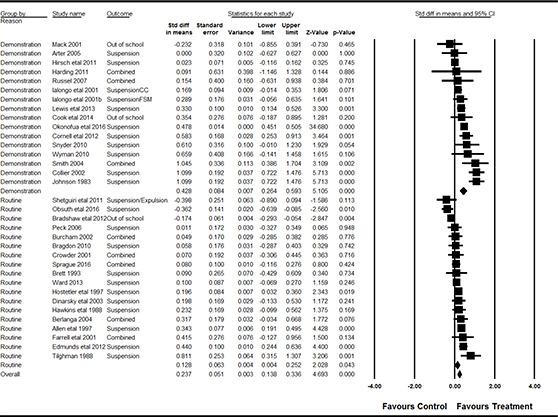

#### Forest plot sub‐group analysis: Evaluator role

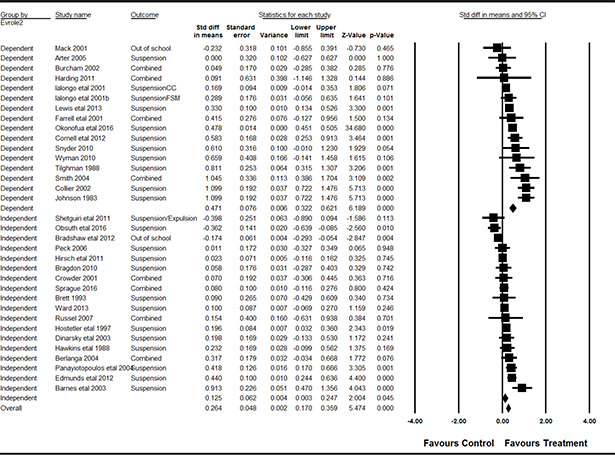

#### Forest plot sub‐group analysis: Type of exclusion

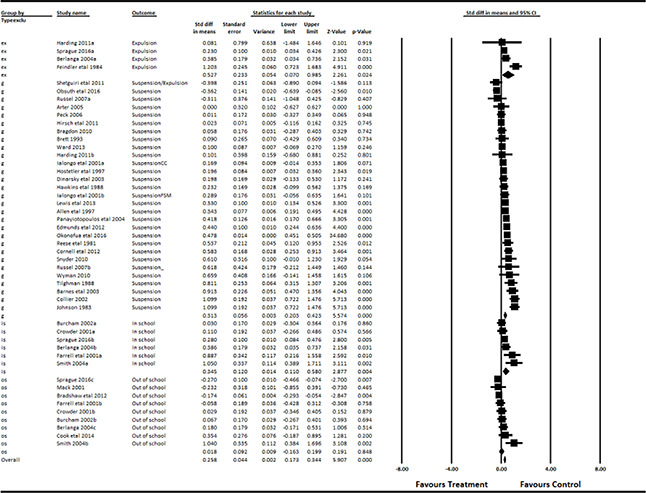

## 12. Data collection instruments

### 12.1 ELIGIBILITY CHECK LIST

**Table:** 

**Criteria**	Evaluation	
1. Does this paper measure school exclusion as an outcome?	⑥ Yes	⑥ No
2. Does the intervention is school based? (or at least one component in the school)	⑥ Yes	⑥ No
3. Are the target individuals school students?	⑥ Yes	⑥ No
4. The report is based in an experimental, quasi‐experimental design?	⑥ Yes	⑥ No
5. Is this report informing statistical results able to be transformed in effect sizes?	⑥ Yes	⑥ No
6. Is this report included?	⑥ Yes	⑥ No
Reasons for exclusion:		

### 12.2 DATA COLLECTION INSTRUMENT

**DATA‐CODING INSTRUMENT**

*School‐based interventions for reducing disciplinary school exclusion. A systematic review*

[Variable names in brackets]

Contents

Section A. Codification

Section B. Bibliographical information

Section C. Ethics

Section D. Research design

Section E. Sample

Section F. Primary outcome coding

Section G. Secondary outcomes coding

Section H. Base‐line measurements

Section I. Programme delivered

Section J. Follow‐up measurement

Section K. Effect sizes

#### Section A. Codification

Instruction: use one data‐coding instrument for each manuscript. When more than one manuscript reports the same research project, select one of them as the principal (e.g., the older) and give it an ID number. The following manuscripts should use the same ID but it must be registered in the Crossref field.

**[STUDYID]** Study ID number:

**[CROSSREF1]** Cross reference document identifier:

**[CROSSREF2]** Cross reference document identifier:

**[CROSSREF3]** Cross reference document identifier:

**[DATESCR]** Date of screening:

**[CODER]** Coder Initials:

#### Section B. Bibliographical information

Before completing this section, please be sure that the manuscript is correctly uploaded in the reference manager programme.

**[AUTHOR]** Name of the main author(s):

**[AFFIL]** Main author affiliation:

**[DATEPUB]** Year of publication:

**[DATEFIEDW]** Year of fieldwork (usually reported in a range):

**[COISTATEMENT]** Has the paper included a conflict of interest statement?

⑥ 1. Yes
⑥ 0. No

**[LANGPUB]** Language of the publication:

⑥ 1. English
⑥ 2. German
⑥ 3. Italian
⑥ 4. Spanish
⑥ 5. Portuguese
⑥ 999. Other:________

**[COUNTPUB]** Country of publication:

⑥ 1. UK
⑥ 2. USA
⑥ 3. Canada
⑥ 4. Australia
⑥ 999. Other:_________
⑥ 99. Unknown

**[TYPUB]** Type of publication:

⑥ 1. Journal
⑥ 2. Book/book chapter
⑥ 3. Masters thesis
⑥ 4. PhD/doctoral thesis
⑥ 5. Technical/governmental report
⑥ 6. Conference proceedings
⑥ 999. Other:________

**[AUTDIS]** Main author discipline:

⑥ 1. Education
⑥ 2. Social Work
⑥ 3. Psychology
⑥ 4. Criminal Justice
⑥ 5. Sociology
⑥ 6. Psychiatry/Medicine
⑥ 999. Other:________
⑥ 99. Unknown

**[LOCAT]** How was the study/report located?

⑥ 1. Electronic database
⑥ 2. Web search
⑥ 3. Reference in a book/paper. Please specify:
⑥ 4. Hand search in specialised journal
⑥ 5. Peer/expert suggestion
⑥ 999. Other. Specify:____________

**Section C. Ethics**

**[CONSENT]** Did the study declare the use of “consent agreement forms”?

⑥ 1. Yes
⑥ 0. No
⑥ 999. Other:________
⑥ 99. Unknown

**[SIGNCONS]** Who signed the consent?

⑥ 1. Students
⑥ 2. Parents
⑥ 3. Teachers
⑥ 4. Schools
⑥ 5. Parents and student
⑥ 999. Other. Specify:______________
⑥ 99. Unknown

#### Section D. Design

The present systematic review includes randomised control trials as well as quasi‐experimental reports (before/after measure plus a control or comparison group). If the control/comparison group is randomly allocated, non‐randomly allocated or matched and no intervention expected to produce impact is provided to it, you will be able to code that group as CONTROL. Subsequently, the TREATMENT group could be understood as the group that receives the intervention, no matter if that condition has been randomly allocated or not.

Please select always the data that is related with the sample effectively analysed.

**[DESTYPE]** What kind of design is this paper based on?

⑥ 1. Randomised controlled trial (true experiment)
⑥ 2. Before‐and‐after with control/comparison group/s
⑥ 3. Instrumental variable
⑥ 4. Propensity score matching
⑥ 5. Interrupted time series
⑥ 6. Pre/post measures with unmatched control/comparison group
⑥ 7. Inverse probability weighting
⑥ 999. Other. Specify:____________

**[RANDUNIT]** Units of randomization

⑥ 1. Individuals
⑥ 2. Clusters/groups (classroom, schools)
⑥ 999. Other. Specify:____________
⑥ 99. Unknown

**[ANALUNIT]** Unit of analysis

⑥ 1. Students
⑥ 2. Clusters/groups (classroom, schools)
⑥ 999. Other. Specify:____________
⑥ 99. Unknown

**[COMPVAR]** Variables measured to create comparability? (e.g., variables used to match the control and treatment groups)

__________________________________________________________________________________________

**[MAINSTAT]** What is the main statistical analysis used to produce the final results?

⑥ 1. Multilevel modelling
⑥ 2. Differences of means
⑥ 3. MANOVA
⑥ 4. Chi‐squared
⑥ 5. Propensity Score Matching
⑥ 999. Other. Specify:____________

#### Section E. Sample

**[SAMPSELECT]** How was the sample selected?

⑥ 1. Randomly
⑥ 2. Assessment
⑥ 3. Self‐selection
⑥ 999. Other. Specify:___________

**[INSAMP]** Initial sample size (i.e., individuals/schools):

**[NUMBFOLL]** Nº of follow‐up:

**[FOLLSAMP1]** Follow‐up 1 sample size:

**[FOLLSAMP2]** Follow‐up 2 sample size:

**[FOLLSAMP3]** Follow‐up 3 sample size:

**[NSCHOOL]** Initial number of schools:

**[NSFOLL1]** Follow‐up 1 sample size:

**[NSFOLL2]** Follow‐up 2 sample size:

**[NSFOLL3]** Follow‐up 3 sample size:

**[NCLASS]** Initial number of classes:

**[NCFOLL1]** Follow‐up 1 sample size:

**[NCFOLL2]** Follow‐up 2 sample size:

**[NCFOLL3]** Follow‐up 3 sample size:

Please code here the information on attrition described in the manuscript:

**Table:** 

** **	Total number of students at Baseline	Total number of students at Follow‐up
Treatment	**[NTREBAS]**	**[NTREFOLL]**
Control	**[NCONTBA]**	**[NCONTFOL]**

**[MEANAGE]** Mean age and standard deviation of overall sample at beginning of intervention:

**[GENDER]** Gender

⑥ % of males
⑥ % of females
⑥ 99. Unknown

**[LOCAT]** Location of programme

⑥ Urban area
⑥ Suburban area
⑥ Rural area
⑥ Mixture of areas
⑥ 99. Not enough information to determine  

**[GRADEX]** Grade level of students

⑥ % of students in Elementary school or equivalent
⑥ % of students in Secondary school or equivalent
⑥ % of students in High school or equivalent
⑥ 4. Other:
⑥ 99. Unknown

**[ETHNI]** Predominant ethnicity^13^

⑥ 1. % of Caucasian:
⑥ 2. % of Black:
⑥ 3. % of Hispanic:
⑥ 4. % of Asian:
⑥ 5. % of other mixed background:
⑥ 99. Unknown

**[COUNTRY]** Please state the name of the country where schools and sample of students were located when tested.

______________________ (99 if unknown)

**[LUNEX]** Socio‐economic status

% of students receiving free/reduced school lunch:

99. Unknown

**[SENEX]** Special Educational Needs

% of students declaring SEN:

99. Unknown

#### Section F. Primary Outcome (School Exclusion)

**[EXCLUSION] Is the manuscript reporting outcomes for school exclusion?**

⑥ 1. Yes
⑥ 0. No

**[TYPEXC]** Type of exclusion measured

⑥ 1. In‐school exclusion
⑥ 2. Out‐of‐school exclusion
⑥ 99. Unknown

**[CHEKTIP]** Duration of school exclusion measured

⑥ 1. Days of Fixed‐term exclusion
(Expressed in number or days, frequencies, percentages)
⑥ 2. Days of Permanent exclusion
(Expressed in number or days, frequencies, percentages)
⑥ 99. Unknown

**[ICCEXCLU]** If the statistical analysis include cluster in MLM, please register the ICC for Exclusion:

#### Section G. Secondary outcomes

**[BEHAVMES]** Did the study include measures on behaviour domains?

⑥ 1. Yes
⑥ 0. No
⑥ 99. Unknown

What types of the following behaviours are measured?

- **[PROSO]** Pro‐social behaviour (e.g., helping, empathy). Specify:____________
  ⑥ 1. Yes
  ⑥ 0. No

**[MEPROSO]** Measure(s) used to test the behaviour (name):

**[ALPHAPROSO]**

⑥ Reliability test. Specify alpha value:_____________
⑥ Non reported

**Table:** 

Groups	Effect size before	Effect size after
Control or comparison	**[PROBC]**	**[PROAC]**
Treatment	**[PROBT]**	**[PROAT]**

**[PAGEPROSO]** Number of the page from where you extract statistical data:

**[ICCPROSO]** If the statistical analysis include cluster in MLM, please register the ICC for behavioural outcomes:

- **[INTERNAL]** Internalising problem behaviour
  (e.g., anxiety, depression, attention‐deficit and hyperactivity disorder (ADHD), attention deficit, hyperactivity). Specify:____________
  ⑥ 1. Yes
  ⑥ 0. No

**[MINTERNAL]** Measure (s) used to test the behaviour (name):

**[ALPHAINTERNAL]**

⑥ Reliability test. Specify alpha value:_____________
⑥ Non reported

**Table:** 

Groups	Effect size before	Effect size after
Control or comparison	**[PROBC]**	**[PROAC]**
Treatment	**[PROBT]**	**[PROAT]**

**[PAGEINTERNAL]** Number of the page from where you extract statistical data:

**[ICCINTERNAL]** If the statistical analysis include cluster in MLM, please register the ICC for behavioural outcomes:

- **[NAEXTERNAL]** Non‐aggressive externalising problem behaviour
  (e.g., stealing, lying, graffiti, illegal drugs). Specify: _____________
  ⑥ 1. Yes
  ⑥ 0. No

**[MNAEXTERNAL]** Measure used to test the behaviour (name):

**[ALPHANAEXTER]**

⑥ Reliability test. Specify alpha value:_____________
⑥ Non reported

**Table:** 

Groups	Effect size before	Effect size after
Control or comparison	**[PROBC]**	**[PROAC]**
Treatment	**[PROBT]**	**[PROAT]**

**[PAGENAXTERN]** Number of the page from where you extract statistical data:

**[ICCNAEXT]** If the statistical analysis include cluster in MLM, please register the ICC for behavioural outcomes:

- **[AAGRESEXT]** Aggressive externalising problem behaviour
  (e.g., Opposition/defiance, physical aggression, indirect aggression, instrumental aggressions/dominance, reactive aggression, school bullying). Specify:_____________
  ⑥ 1. Yes
  ⑥ 0. No

**[MAGRESSEXT]** Measure used to test the behaviour (name):

**[ALPHAAEXT]**

⑥ Reliability test. Specify alpha value:_____________
⑥ Non reported

**Table:** 

Groups	Effect size before	Effect size after
Control or comparison	**[PROBC]**	**[PROAC]**
Treatment	**[PROBT]**	**[PROAT]**

**[AGRESPAGE]** Number of the page from where you extract statistical data:

**[ICCAGREEX]** If the statistical analysis include cluster in MLM, please register the ICC for Behavioural outcomes:

#### Section H. Base‐line measurements

**[DATABAS]** Date of baseline assessment:

What measures were used?

**[SRMES]** Self‐report

⑥ 1. Yes
⑥ 0. No
⑥ 99. Unknown

**[TRMES]** Teachers’ report

⑥ 1. Yes
⑥ 0. No
⑥ 99. Unknown

**[SCHRMES]** School records

⑥ 1. Yes
⑥ 0. No
⑥ 99. Unknown

**[PAREP]** Parents

⑥ 1. Yes
⑥ 0. No
⑥ 99. Unknown

**[OMES]** Other:_________________

**[EXCBL]** Frequency of exclusion at baseline (register any measure given by the study)

#### Section I. Programme delivered

This section aims to codify data on the delivery process. Be aware that sometimes final reports do not describe all the data related to delivery. In those cases it would be helpful to search for registered protocols or earlier publications reporting more data on this.

**[PRONAME]** Name of the programme:

**[PROCURRI]** Was the programme curricular?

⑥ 1. Yes
⑥ 0. No
⑥ 99. Unknown
⑥ 999. Other. Specify:____________

**[PROEND]** The programme was conducted for:

⑥ 1. Research ends
⑥ 2. Demonstration ends
⑥ 3. Routine
⑥ 99. Unknown
⑥ 999. Other. Specify:_____________

**[PROSIT]** Primary programme site:

⑥ 1. Public school
⑥ 2. Private school
⑥ 3. Other, (specify):_________
⑥ 99. Unknown

**[PROSCH]** Was at least one of the components of the intervention was settled at school?

⑥ 1. Yes
⑥ 0. No
⑥ 99. Unknown

**[PRODEL]** Who delivered the programme?

⑥ 1. External facilitators
⑥ 2. School facilitators
⑥ 3. Both
⑥ 99. Unknown

**[PDBACK]** Deliverer's background 1

⑥ 1. Social worker
⑥ 2. Psychologist
⑥ 3. Teacher
⑥ 4. Police officers
⑥ 5. Peers
⑥ 999. Other. Specify:_____________
⑥ 99. Unknown

**[PDBACK]** Deliverer's background 2

⑥ 1. Social worker
⑥ 2. Psychologist
⑥ 3. Teacher
⑥ 4. Police officers
⑥ 5. Peers
⑥ 999. Other. Specify:_____________
⑥ 99. Unknown

**[TRAINBEF]** Did the deliverer receive training BEFORE implementing the programme?

⑥ 1. Yes.
⑥ 0. No.
⑥ 99. Unknown

**[THOURS]** How long was the training in hours?:____________

**[TRAINDUR]** Did the deliverer receive training DURING the implementation?

⑥ 1. Yes.
⑥ 0. No.
⑥ 99. Unknown

**[THOURS2]** How long was the training in hours?:

What type of intervention was delivered? If the manuscript indicates a mixture of interventions you can select more than one using TYPEPRO 1, 2 and 3.

**Table:** 

** **	**[TYPEPRO1]**	**[TYPEPRO2]**	**[TYPEPRO3]**
1. Mentoring programme	** **	** **	** **
2. Restorative programme	** **	** **	** **
3. Skills training programme	** **	** **	** **
4. School‐wide systemic intervention	** **	** **	** **
5. Classroom management	** **	** **	** **
6. Counselling/therapy	** **	** **	** **
999. Other	** **	** **	** **

Theoretical background of the intervention. If the manuscript indicates a mixture of theories, you can select more than one using THEORY 1, 2 and 3.

**Table:** 

** **	**[THEORY1]**	**[THEORY2]**	**[THEORY3]**
1. Cognitive behavioural	** **	** **	** **
2. Learning theory	** **	** **	** **
3. Restorative theories	** **	** **	** **
4. Organisational theories or principles	** **	** **	** **
99. Unknown	** **	** **	** **
999. Other (Specify)	** **	** **	** **

**[PROCONT]** What happened to the control group?

⑥ 1. No intervention
⑥ 2. Wait‐list control
⑥ 3. Minimal contact
⑥ 4. Treatment as usual
⑥ 5. Alternative treatment
⑥ 5. Placebo
⑥ 999. Other. Specify:____________

**[PROFORM]** Delivery format:

⑥ 1. Manualised programme
⑥ 2. Unstructured programme
⑥ 3. Mixed
⑥ 99. Unknown
⑥ 999. Other. Specify:____________

What was the programme dosage?

**[PRODOSW]** AVERAGE Duration in weeks:

**[PRODOSH]** AVERAGE Hours per week:

**[PROFREQ]** What was the frequency of the programme counted?

⑥ 1. Less than a week
⑥ 2. Once a week
⑥ 3. Twice a week
⑥ 4. 3‐4 times a week
⑥ 5. Daily
⑥ 99. Unknown

**[EVROLE]** What was the “evaluator” role?

⑥ 1. Deliver the programme
⑥ 2. Designed the programme
⑥ 3. Both design and delivery
⑥ 4. Independent evaluator
⑥ 99. Unknown

**[MONITOR]** Was the programme implementation monitored?

⑥ 1. Yes
⑥ 0. No
⑥ 99. Unknown. Not enough information

**[IMPROB]** Does the report provide information about implementation problems?

⑥ 1. Yes, there were clear problems which are reported
⑥ 0. No, non‐reported problems, reasonably well implemented
⑥ 2. Possible problems based on the description of the intervention
⑥ 99. Unknown. Not enough information

**[PROCOST]** Is the cost of the intervention mentioned?

⑥ 1. Yes
⑥ 0. No

**[AMOUNT]** Cost:

**[UNITCURR]** Currency:

#### Section J. Follow‐up measurement

**[DATEFALL]** Date of follow up:

Multiple follow‐ups

**[MONTHFO1]** Nº of months from baseline to 1^st^ follow‐up:

**[MONTHFO2]** Nº of months from baseline to 2^nd^ follow‐up:

**[MONTHFO3]** Nº of months from baseline to 3^rd^ follow‐up:

**[MONTHFO4]** Nº of months from baseline to 4^th^ follow‐up:

What measures were used?

**[POSTSR]** Children/adolescent self‐report

⑥ 1. Yes
⑥ 0. No

**[POSTTR]** Teachers’ report

⑥ 1. Yes
⑥ 0. No

**[POSTSR]** School records

⑥ 1. Yes
⑥ 0. No

**[POSTPR]** Parents report

⑥ 1. Yes
⑥ 0. No

**[POSTO]** Other:__________

**[FREQEXFOLL]** Frequency of exclusion at follow‐up (register any measure given by the study)

#### Section K. Effect sizes of intervention on school exclusion

- Effect size: outcomes expressed in continuous data.

**[CSSEX]** Sample size for the ES (Treatment group)

**[CSSCON]** Sample size for the ES (Control group)

**[MEANEX]** Mean (Treatment group)

**[MEANCON]** Mean (Control group)

**[MEANADJ]** Are the Means adjusted?

⑥ 1. Yes.
⑥ 0. No

**[ADJBY]** Adjusted by (describe):______________

**[SDEX]** Standard deviation (Treatment group)

**[SDCON]** Standard deviation (Control group)

**[SEEX]** Standard error (Treatment group)

**[SECON]** Standard error (Control group)

**[CORREX]** Correlation coefficient + *p* value (Treatment group)

**[CORRCON]** Correlation coefficient + *p* value (Control group)

**[SMDTREAT]** Standardised mean difference + confidence intervals

- Effect size: outcomes expressed in dichotomous data.

**[DSSTRE]** Sample size for the ES (Treatment group)

**[DSSCONT]** Sample size for the ES (Control group)

**[NUMTRE]** Treatment group; number of successful cases:

**[NUMCON]** Control group; number of successful cases:

**[PROPTRE]** Treatment group; proportion of successful cases:

**[PROPCON]** Control group; proportion of successful cases:

**[ORTRE]** Treatment group; odds ratios:

Confidence Intervals:
*p*‐value:

**[ORCON]** Control group; odds ratios:

Confidence Intervals:
*p*‐value:

**[ORADJ]** Are the odds ratios adjusted?

⑥ 1. Yes.
⑥ 0. No

Adjusted by (explain):_________

**[CHISC]** X^2^ value with *df:*

**[PAGEEFFECT]** Number of the page from where you extract statistical data:

- Effect sizes at follow‐up

**[ESFOLLOW1]** Calculated effect at follows up 1:______

[ESFOLL1] Number of months after intervention for follow‐up 1:______

**[ESFOLLOW2]** Calculated effect at follows up 2:______

[ESFOLL2] Number of months after intervention for follow‐up 2:______

**[ESFOLLOW3]** Calculated effect at follows up 3:______

[ESFOLL3] Number of months after intervention for follow‐up 3:______

**[ESFOLLOW4]** Calculated effect at follows up 4:______

[ESFOLL4] Number of months after intervention for follow‐up 4:______

### 12.3 EPOC ‘RISK OF BIAS’ TOOL

**Table:** 

**Study:**	** **	**Date:**	**Coder:**
**Item**	**Criteria**	**Evaluation**	**Justification**
**1**	**Was the allocation sequence adequately generated?** Score “Low risk” if a random component in the sequence generation process is described (eg., Referring to a random number table). Score “High risk” when a non‐random method is used (eg performed by date of admission). NRCTs and CBA studies should be scored “High risk”. Score “Unclear risk” if not specified in the paper.		
**2**	**Was the allocation adequately concealed?** Score “Low risk” if the unit of allocation was by institution, team or professional and allocation was performed on all units at the start of the study; or if the unit of allocation was by patient or episode of care and there was some form of centralised randomisation scheme, an on‐site computer system or sealed opaque envelopes were used. CBA studies should be scored “High risk”. Score “Unclear risk” if not specified in the paper.		
**3**	**Were baseline outcome measurements similar?^1,2^** Score “Low risk” if performance or patient outcomes were measured prior to the intervention, and no important differences were present across study groups. In RCTs, score “Low risk” if imbalanced but appropriate adjusted analysis was performed (e.g. Analysis of covariance). Score “High risk” if important differences were present and not adjusted for in analysis. If RCTs have no baseline measure of outcome, score “Unclear risk”		
**4**	**Were baseline characteristics similar?** Score “Low risk” if baseline characteristics of the study and control providers are reported and similar. Score “Unclear risk” if it is not clear in the paper (e.g. characteristics are mentioned in text but no data were presented). Score “High risk” if there is no report of characteristics in text or tables or if there are differences between control and intervention providers. Note that in some cases imbalance in patient characteristics may be due to recruitment bias whereby the provider was responsible for recruiting patients into the trial.		
**5**	**Were incomplete outcome data adequately addressed?** ^1^ Score “Low risk” if missing outcome measures were unlikely to bias the results (e.g. the proportion of missing data was similar in the intervention and control groups or the proportion of missing data was less than the effect size i.e. unlikely to overturn the study result). Score “High risk” if missing outcome data was likely to bias the results. Score “Unclear risk” if not specified in the paper (Do not assume 100% follow up unless stated explicitly).		
**6**	**Was knowledge of the allocated interventions adequately prevented during the study?** ^1^ Score “Low risk” if the authors state explicitly that the primary outcome variables were assessed blindly, or the outcomes are objective, e.g. length of hospital stay. Primary outcomes are those variables that correspond to the primary hypothesis or question as defined by the authors. Score “High risk” if the outcomes were not assessed blindly. Score “Unclear risk” if not specified in the paper.		
**7**	**Was the study adequately protected against contamination?** Score “Low risk” if allocation was by community, institution or practice and it is unlikely that the control group received the intervention. Score “High risk” if it is likely that the control group received the intervention (e.g. if patients rather than professionals were randomised). Score “Unclear risk” if professionals were allocated within a clinic or practice and it is possible that communication between intervention and control professionals could have occurred (e.g. physicians within practices were allocated to intervention or control) ** **		
**8**	**Was the study free from selective outcome reporting?** Score “Low risk” if there is no evidence that outcomes were selectively reported (e.g. all relevant outcomes in the methods section are reported in the results section). Score “High risk” if some important outcomes are subsequently omitted from the results. Score “Unclear risk” if not specified in the paper.		

### 12.4 TRICHOTOMOUS SCALE FOR POTENTIAL FINANCIAL CONFLICT OF INTEREST

**Table:** 

**Study:**	** **	**Date:**	**Coder:**
**Financial COI**	**Coding Rule**	**Justification**	
**Unlikely**	None of the study authors is programme developer, collaborator of programme developer or license holder.		
**Possible**	(Programme developer or collaborator of programme developer is study author) AND ((programme is not (yet) commercially available) OR (business model is ‘not‐for‐profit’))		
**Likely**	(Programme developer or collaborator of programme developer is study author) AND (programme is commercially available) AND (business model is ‘for profit’)		

^*^This instrument has been developed by Eisner & Humphreys (2012)

## 13. Electronic searches

**Australian Education Index**

Date: 05/10/ 2015

Output of the searches: 148

Saved hits: 1

Final searches string:

1) school exclusion
2) school suspension
3) school suspended
4) school expelled
5) school expulsion
6) school stand down

**British Education Index**

Date: 05/10/ 2015

Output of the searches: 202

Saved hits: 5

Final searches string:

1) school exclusion
2) school suspension
3) school suspended
4) school expelled
5) school expulsion
6) school AND stand down

**BMJ Controlled Trials**

Date: 06/10/ 2015

Output of the searches: 550

Saved hits: 0

Final searches string:

1) school exclusion
2) school suspension
3) school suspended
4) school expelled
5) school expulsion
6) school stand down

**CBCA Education (Canada)**

Date: 14/10/2015

Output of the searches: 5652

Saved hits: 58

Final searches string:

1) school exclusion
2) school suspension
3) school expelled
4) school expulsion
5) school stand down

ClinicalTrials.gov

Date: 07/10/ 2015

Output of the searches: 285

Saved hits: 3

Final searches string:

1) school exclusion
2) school suspension
3) school suspended
4) school expelled
5) school expulsion
6) school stand down

**Criminal Justice Abstract**

Date: 06/10/ 2015

Output of the searches: 369

Saved hits: 19

Final searches string:

1) school exclusion
2) school suspension
3) school suspended
4) school expelled
5) school expulsion
6) school stand down

**Cochrane Central Register of Controlled Trials (CENTRAL)**

Date: 16/10/2015

Output of the searches: 154

Saved hits: 12

Final searches string:

1) school exclusion
2) school suspension
3) school suspended
4) school expelled
5) school expulsion
6) school stand down

**Educational Resources Information Center ‐ ERIC**

Date: 14/10/2015

Output of the searches: 1491

Saved hits: 48

Final searches string:

1) school exclusion
2) school suspension
3) school suspended
4) school expelled
5) school expulsion
6) school stand down

**Ethos Beta**

Date: 08/10/ 2015

Output of the searches: 482

Saved hits: 5

Final searches string:

1) school exclusion
2) school suspension
3) school suspended
4) school expelled
5) school expulsion
6) school stand down

**EMBASE**

Date: From 08/10/ 2015 to 12/10/2015

Output of the searches: 1569

Saved hits: 12

Final searches string:

1) “school exclusion” AND
2) “school exclusion” AND evaluation
3) “school exclusion” AND random*
4) “school exclusion” AND intervention,
5) “school exclusion” AND effective*,
6) “school exclusion” AND efficacy,
7) “school exclusion” AND quasi,
8) “school exclusion” AND impact,
9) “school exclusion” AND RCT,
10) “school exclusion” AND school management
11) “school exclusion” AND classroom management
12) “school exclusion” AND school support project*
13) “school exclusion” AND skills training
14) “school exclusion” AND disciplinary methods
15) “school exclusion” AND token economy
16) “school exclusion” AND program*
17) “school exclusion” AND *intervention*
18) “school exclusion” AND strateg*
19) “school exclusion” AND schoolchildren
20) “school exclusion” AND *children*
21) “school exclusion” AND school‐age*
22) “school exclusion” AND adolescent*
23) “school exclusion” AND pupil*
24) “school exclusion” AND student*
25) “school suspension” AND experiment*
26) “school suspension” AND evaluation
27) “school suspension” AND random*
28) “school suspension” AND intervention,
29) “school suspension” AND effective*,
30) “school suspension” AND efficacy,
31) “school suspension” AND quasi,
32) “school suspension” AND impact,
33) “school suspension” AND RCT,
34) “school suspension” AND school management
35) “school suspension” AND classroom management
36) “school suspension” AND school support project*
37) “school suspension” AND skills training
38) “school suspension” AND disciplinary methods
39) “school suspension” AND token economy
40) “school suspension” AND program*
41) “school suspension” AND *intervention*
42) “school suspension” AND strateg*
43) “school suspension” AND schoolchildren
44) “school suspension” AND *children*
45) “school suspension” AND school‐age*
46) “school suspension” AND adolescent*
47) “school suspension” AND pupil*
48) “school suspension” AND student*
49) “school expulsion” AND experiment*
50) “school expulsion” AND evaluation
51) “school expulsion” AND random*
52) “school expulsion” AND intervention,
53) “school expulsion” AND effective*,
54) “school expulsion” AND efficacy,
55) “school expulsion” AND quasi,
56) “school expulsion” AND impact,
57) “school expulsion” AND RCT,
58) “school expulsion” AND school management
59) “school expulsion” AND classroom management
60) “school expulsion” AND school support project*
61) “school expulsion” AND skills training
62) “school expulsion” AND disciplinary methods
63) “school expulsion” AND token economy
64) “school expulsion” AND program*
65) “school expulsion” AND *intervention*
66) “school expulsion” AND strateg*
67) “school expulsion” AND schoolchildren
68) “school expulsion” AND *children*
69) “school expulsion” AND school‐age*
70) “school expulsion” AND adolescent*
71) “school expulsion” AND pupil*
72) “school expulsion” AND student*

**Google Scholar**

Date: From 27/10/ 2015 to 04/11/2015

Output of the searches: 13525

Saved hits: 165

Final searches string:

1) “school exclusion” AND experiment OR evaluation experiment OR evaluation OR quasi OR effective* OR RCT OR impact OR efficacy OR intervention OR random*
2) “school exclusion” AND school management OR classroom management OR school support project* OR skills training OR disciplinary methods OR token economy OR program* OR *intervention* OR strateg*
3) “school suspen*” AND experiment* OR evaluation OR quasi OR effective* OR RCT OR impact OR efficacy OR intervention OR random*
4) “school suspen*” AND school management OR classroom management OR school support project* OR skills training OR disciplinary methods OR token economy OR program* OR *intervention* OR strateg*
5) “school expulsion” AND experiment OR evaluation experiment OR evaluation OR quasi OR effective* OR RCT OR impact OR efficacy OR intervention OR random*
6) “school expulsion” AND school management OR classroom management OR school support project* OR skills training OR disciplinary methods OR token economy OR program* OR *intervention* OR strateg*
7) “stand‐down” AND experiment* OR evaluation OR quasi OR effective* OR RCT OR impact OR efficacy OR intervention OR random*
8) “stand‐down” AND school management OR classroom management OR school support project* OR skills training OR disciplinary methods OR token economy OR program* OR *intervention* OR strateg*

[In order to manage the searches we break down the searches by year. For instance 1980‐1990, 1991‐2005 and 2006 to date]

**Google**

Date: From 27/10/ 2015 to 04/11/2015

Output of the searches: 4092

Saved hits: 22

Final searches string:

1) “school exclusion” AND experiment* OR evaluation OR quasi OR RCT OR impact OR efficacy OR intervention
2) “school exclusion” AND random*
3) “school exclusion” AND effective*
4) “school exclusion” AND school management OR classroom management OR school support project* OR skills training OR disciplinary methods OR token economy
5) “school exclusion” AND *intervention*
6) “school exclusion” AND strateg*
7) “school exclusion” AND program*
8) “school suspension” AND experiment* OR evaluation OR quasi OR RCT OR impact OR efficacy OR intervention
9) “school suspension” AND random*
10) “school suspension” AND effective*
11) “school suspension” AND school management OR classroom management OR school support project* OR skills training OR disciplinary methods OR token economy
12) “school suspension” AND *intervention*
13) “school suspension” AND strateg*
14) “school suspension” AND program*
15) school expulsion AND…intervention* OR strategy* OR program*
16) school expulsion AND…random* OR effective*
17) school expulsion AND…school management OR classroom management OR school support project* OR skills training OR disciplinary methods OR token economy
18) school expulsion AND…random* OR effective
19) school expulsion AND…experiment* OR evaluation OR quasi OR RCT OR impact OR efficacy OR intervention

**Institute of Education Sciences – What Works Clearinghouse**

Date: 30/09/ 2015

Output of the searches: 2

Saved hits: 0

Final searches string:

1) school exclusion
2) school suspension
3) school suspended
4) school expelled
5) school expulsion
6) school stand down

**ISI Web of Knowledge**

Date: from 07/09/ 2015 to 17/09/2015

Output of the searches: 4391

Saved hits: 270

Final searches string:

1) TOPIC: (experiment*) OR TOPIC: (evaluation) OR TOPIC: (random*) OR TOPIC: (intervention) OR TOPIC: (effective*) OR TOPIC: (efficacy) ORTOPIC: (quasi) OR TOPIC: (impact) OR TOPIC: (RCT)
  AND
  TOPIC: (school) AND TOPIC: (*exclusion)
2) TOPIC: (school) AND TOPIC: (*suspension*)
  AND
  TOPIC: (experiment*) OR TOPIC: (evaluation) OR TOPIC: (random*) OR TOPIC: (intervention) OR TOPIC: (effective*) OR TOPIC: (efficacy) ORTOPIC: (quasi) OR TOPIC: (impact) OR TOPIC: (RCT)
3) TOPIC: (school) AND TOPIC: (suspended)
  AND
  TOPIC: (experiment*) OR TOPIC: (evaluation) OR TOPIC: (random*) OR TOPIC: (intervention) OR TOPIC: (effective*) OR TOPIC: (efficacy) ORTOPIC: (quasi) OR TOPIC: (impact) OR TOPIC: (RCT)
  Refined by: RESEARCH AREAS: (PSYCHOLOGY OR FAMILY STUDIES OR EDUCATION EDUCATIONAL RESEARCH OR BEHAVIORAL SCIENCES OR PSYCHIATRY OR CRIMINOLOGY PENOLOGY OR SOCIOLOGY OR ETHNIC STUDIES OR SOCIAL WORK OR URBAN STUDIES OR SOCIAL SCIENCES OTHER TOPICS OR SOCIAL ISSUES)
  Timespan=1980‐2015
4) TOPIC: (school) AND TOPIC: (expelled)
  AND
  TOPIC: (experiment*) OR TOPIC: (evaluation) OR TOPIC: (random*) OR TOPIC: (intervention) OR TOPIC: (effective*) OR TOPIC: (efficacy) ORTOPIC: (quasi) OR TOPIC: (impact) OR TOPIC: (RCT)
5) TOPIC: (school) AND TOPIC: (expulsion)
  AND
  TOPIC: (experiment*) OR TOPIC: (evaluation) OR TOPIC: (random*) OR TOPIC: (intervention) OR TOPIC: (effective*) OR TOPIC: (efficacy) ORTOPIC: (quasi) OR TOPIC: (impact) OR TOPIC: (RCT)
6) TOPIC: (school management) OR TOPIC: (classroom management) OR TOPIC: (school support project*) OR TOPIC: (skills training) OR TOPIC:(disciplinary methods) OR TOPIC: (token economy) OR TOPIC: (program*) OR TOPIC: (*intervention*) OR TOPIC: (strateg*)
  AND
  TOPIC: (school) AND TOPIC: (*exclusion)
7) TOPIC: (school management) OR TOPIC: (classroom management) OR TOPIC: (school support project*) OR TOPIC: (skills training) OR TOPIC:(disciplinary methods) OR TOPIC: (token economy) OR TOPIC: (program*) OR TOPIC: (*intervention*) OR TOPIC: (strateg*)
  AND
  TOPIC: (school) AND TOPIC: (*exclusion)
8) TOPIC: (school management) OR TOPIC: (classroom management) OR TOPIC: (school support project*) OR TOPIC: (skills training) OR TOPIC:(disciplinary methods) OR TOPIC: (token economy) OR TOPIC: (program*) OR TOPIC: (*intervention*) OR TOPIC: (strateg*)
  AND
  TOPIC: (school) AND TOPIC: (*suspension*)
9) TOPIC: (school management) OR TOPIC: (classroom management) OR TOPIC: (school support project*) OR TOPIC: (skills training) OR TOPIC:(disciplinary methods) OR TOPIC: (token economy) OR TOPIC: (program*) OR TOPIC: (*intervention*) OR TOPIC: (strateg*)
  AND
  TOPIC: (school) AND TOPIC: (suspended)
10) TOPIC: (school management) OR TOPIC: (classroom management) OR TOPIC: (school support project*) OR TOPIC: (skills training) OR TOPIC:(disciplinary methods) OR TOPIC: (token economy) OR TOPIC: (program*) OR TOPIC: (*intervention*) OR TOPIC: (strateg*)
  AND
  TOPIC: (school) AND TOPIC: (expelled)
11) TOPIC: (school management) OR TOPIC: (classroom management) OR TOPIC: (school support project*) OR TOPIC: (skills training) OR TOPIC:(disciplinary methods) OR TOPIC: (token economy) OR TOPIC: (program*) OR TOPIC: (*intervention*) OR TOPIC: (strateg*)
  AND
  TOPIC: (school) AND TOPIC: (expulsion)
12) TOPIC: (schoolchildren) OR TOPIC: (*children*) OR TOPIC: (school‐age*) OR TOPIC: (adolescent*) OR TOPIC: (pupil*) OR TOPIC: (student)
  AND
  TOPIC: (school) AND TOPIC: (*exclusion)
13) TOPIC: (schoolchildren) OR TOPIC: (*children*) OR TOPIC: (school‐age*) OR TOPIC: (adolescent*) OR TOPIC: (pupil*) OR TOPIC: (student)
  AND
  TOPIC: (school) AND TOPIC: (*suspension*)
14) TOPIC: (schoolchildren) OR TOPIC: (*children*) OR TOPIC: (school‐age*) OR TOPIC: (adolescent*) OR TOPIC: (pupil*) OR TOPIC: (student)
  AND
  TOPIC: (school) AND TOPIC: (suspended)
15) TOPIC: (schoolchildren) OR TOPIC: (*children*) OR TOPIC: (school‐age*) OR TOPIC: (adolescent*) OR TOPIC: (pupil*) OR TOPIC: (student)
  AND
  TOPIC: (school) AND TOPIC: (expelled)
16) TOPIC: (school management) OR TOPIC: (classroom management) OR TOPIC: (school support project*) OR TOPIC: (skills training) OR TOPIC:(disciplinary methods) OR TOPIC: (token economy) OR TOPIC: (program*) OR TOPIC: (*intervention*) OR TOPIC: (strateg*)
  AND
  TOPIC: (school) AND TOPIC: (expulsion)

All searches in ISI Web of Knowledge were Refined by: RESEARCH AREAS: (PSYCHOLOGY OR FAMILY STUDIES OR EDUCATION EDUCATIONAL RESEARCH OR BEHAVIORAL SCIENCES OR PSYCHIATRY OR CRIMINOLOGY PENOLOGY OR SOCIOLOGY OR ETHNIC STUDIES OR SOCIAL WORK OR URBAN STUDIES OR SOCIAL SCIENCES OTHER TOPICS OR SOCIAL ISSUES)

Timespan=1980‐2015

**MEDLINE**

Date: 05/10/ 2015

Output of the searches: 142

Saved hits: 0

Final searches string:

1) “school exclusion” AND experiment*
2) “school exclusion” AND evaluation
3) “school exclusion” AND random*
4) “school exclusion” AND intervention,
5) “school exclusion” AND effective*,
6) “school exclusion” AND efficacy,
7) “school exclusion” AND quasi,
8) “school exclusion” AND impact,
9) “school exclusion” AND RCT,
10) “school exclusion” AND school management
11) “school exclusion” AND classroom management
12) “school exclusion” AND school support project*
13) “school exclusion” AND skills training
14) “school exclusion” AND disciplinary methods
15) “school exclusion” AND token economy
16) “school exclusion” AND program*
17) “school exclusion” AND *intervention*
18) “school exclusion” AND strateg*
19) “school exclusion” AND schoolchildren
20) “school exclusion” AND *children*
21) “school exclusion” AND school‐age*
22) “school exclusion” AND adolescent*
23) “school exclusion” AND pupil*
24) “school exclusion” AND student*
25) “school suspension” AND experiment*
26) “school suspension” AND evaluation
27) “school suspension” AND random*
28) “school suspension” AND intervention,
29) “school suspension” AND effective*,
30) “school suspension” AND efficacy,
31) “school suspension” AND quasi,
32) “school suspension” AND impact,
33) “school suspension” AND RCT,
34) “school suspension” AND school management
35) “school suspension” AND classroom management
36) “school suspension” AND school support project*
37) “school suspension” AND skills training
38) “school suspension” AND disciplinary methods
39) “school suspension” AND token economy
40) “school suspension” AND program*
41) “school suspension” AND *intervention*
42) “school suspension” AND strateg*
43) “school suspension” AND schoolchildren
44) “school suspension” AND *children*
45) “school suspension” AND school‐age*
46) “school suspension” AND adolescent*
47) “school suspension” AND pupil*
48) “school suspension” AND student*
49) “school expulsion” AND experiment*
50) “school expulsion” AND evaluation
51) “school expulsion” AND random*
52) “school expulsion” AND intervention,
53) “school expulsion” AND effective*,
54) “school expulsion” AND efficacy,
55) “school expulsion” AND quasi,
56) “school expulsion” AND impact,
57) “school expulsion” AND RCT,
58) “school expulsion” AND school management
59) “school expulsion” AND classroom management
60) “school expulsion” AND school support project*
61) “school expulsion” AND skills training
62) “school expulsion” AND disciplinary methods
63) “school expulsion” AND token economy
64) “school expulsion” AND program*
65) “school expulsion” AND *intervention*
66) “school expulsion” AND strateg*
67) “school expulsion” AND schoolchildren
68) “school expulsion” AND *children*
69) “school expulsion” AND school‐age*
70) “school expulsion” AND adolescent*
71) “school expulsion” AND pupil*
72) “school expulsion” AND student*

**The National dropout prevention centre network**

Date: 16/10/2015

Output of the searches: 26

Saved hits: 0

Final searches string:

1) school exclusion
2) school suspension
3) school suspended
4) school expelled
5) school expulsion
6) school stand down

**The Netherlands Trial Register ‐ NTR**

Date: 07/10/ 2015

Output of the searches: 0

Saved hits: 0

Final searches string:

1) school exclusion
2) school suspension
3) school suspended
4) school expelled
5) school expulsion
6) school stand down

**Open Grey**

Date: 07/10/ 2015

Output of the searches: 169

Saved hits: 3

Final searches string:

1) school exclusion
2) school suspension
3) school suspended
4) school expelled
5) school expulsion
6) school stand down

**ProQuest Dissertations & Theses A&I: Social Sciences**

**Including: Dissertation Abstracts & Index to Theses Database**

Date: 19/10/2015

Output of the searches: 5280

Saved hits: 344

Final searches string:

1) “school exclusion” AND experiment
2) “school exclusion” AND evaluation
3) “school exclusion” AND random*
4) “school exclusion” AND intervention
5) “school exclusion” AND effective*
6) “school exclusion” AND efficacy
7) “school exclusion” AND quasi
8) “school exclusion” AND impact
9) “school exclusion” AND RCT
10) “school exclusion” AND school management
11) “school exclusion” AND classroom management
12) “school exclusion” AND school support project*
13) “school exclusion” AND skills training
14) “school exclusion” AND disciplinary methods
15) “school exclusion” AND token economy
16) “school exclusion” AND program*
17) “school exclusion” AND strateg*
18) “school suspension” AND experiment
19) “school suspension” AND evaluation
20) “school suspension” AND random*
21) “school suspension” AND intervention
22) “school suspension” AND effective*
23) “school suspension” AND efficacy
24) “school suspension” AND quasi
25) “school suspension” AND impact
26) “school suspension” AND RCT
27) “school suspension” AND school management
28) “school suspension” AND classroom management
29) “school suspension” AND school support project*
30) “school suspension” AND skills training
31) “school suspension” AND disciplinary methods
32) “school suspension” AND token economy
33) “school suspension” AND program*
34) “school suspension” AND strateg*
35) “school stand‐down” AND experiment
36) “school stand‐down” AND evaluation
37) “school stand‐down” AND random*
38) “school stand‐down” AND intervention
39) “school stand‐down” AND effective*
40) “school stand‐down” AND efficacy
41) “school stand‐down” AND quasi
42) “school stand‐down” AND impact
43) “school stand‐down” AND RCT
44) “school stand‐down” AND school management
45) “school stand‐down” AND classroom management
46) “school stand‐down” AND school support project*
47) “school stand‐down” AND skills training
48) “school stand‐down” AND disciplinary methods
49) “school stand‐down” AND token economy
50) “school stand‐down” AND program*
51) “school stand‐down” AND strateg*
52) “school expelled” AND experiment
53) “school expelled” AND evaluation
54) “school expelled” AND random*
55) “school expelled” AND intervention
56) “school expelled” AND effective*
57) “school expelled” AND efficacy
58) “school expelled” AND quasi
59) “school expelled” AND impact
60) “school expelled” AND RCT
61) “school expelled” AND school management
62) “school expelled” AND classroom management
63) “school expelled” AND school support project*
64) “school expelled” AND skills training
65) “school expelled” AND disciplinary methods
66) “school expelled” AND token economy
67) “school expelled” AND program*
68) “school expelled” AND strateg*
69) “school expulsion” AND experiment
70) “school expulsion” AND evaluation
71) “school expulsion” AND random*
72) “school expulsion” AND intervention
73) “school expulsion” AND effective*
74) “school expulsion” AND efficacy
75) “school expulsion” AND quasi
76) “school expulsion” AND impact
77) “school expulsion” AND RCT
78) “school expulsion” AND school management
79) “school expulsion” AND classroom management
80) “school expulsion” AND school support project*
81) “school expulsion” AND skills training
82) “school expulsion” AND disciplinary methods
83) “school expulsion” AND token economy
84) “school expulsion” AND program*
85) “school expulsion” AND strateg*
86) “school suspended” AND experiment
87) “school suspended” AND evaluation
88) “school suspended” AND random*
89) “school suspended” AND intervention
90) “school suspended” AND effective*
91) “school suspended” AND efficacy
92) “school suspended” AND quasi
93) “school suspended” AND impact
94) “school suspended” AND RCT
95) “school suspended” AND school management
96) “school suspended” AND classroom management
97) “school suspended” AND school support project*
98) “school suspended” AND skills training
99) “school suspended” AND disciplinary methods
100) “school suspended” AND token economy
101) “school suspended” AND program*
102) “school suspended” AND strateg*

**PsychINFO**

Date: From 12/10/ 2015 to 14/10/2015

Output of the searches: 1538

Saved hits: 86

Final searches string:

1) school exclusion
2) school suspension
3) school suspended
4) school expelled
5) school expulsion
6) school stand down

**Sociological Abstract (ProQuest)**

Date: From 21/09/ 2015 to 23/09/2015

Output of the searches: 2440

Saved hits: 355

Final searches string:

1) “school exclusion” AND experiment*
2) “school exclusion” AND evaluation
3) “school exclusion” AND random*
4) “school exclusion” AND intervention,
5) “school exclusion” AND effective*,
6) “school exclusion” AND efficacy,
7) “school exclusion” AND quasi,
8) “school exclusion” AND impact,
9) “school exclusion” AND RCT,
10) “school exclusion” AND school management
11) “school exclusion” AND classroom management
12) “school exclusion” AND school support project*
13) “school exclusion” AND skills training
14) “school exclusion” AND disciplinary methods
15) “school exclusion” AND token economy
16) “school exclusion” AND program*
17) “school exclusion” AND *intervention*
18) “school exclusion” AND strateg*
19) “school exclusion” AND schoolchildren
20) “school exclusion” AND *children*
21) “school exclusion” AND school‐age*
22) “school exclusion” AND adolescent*
23) “school exclusion” AND pupil*
24) “school exclusion” AND student*
25) “school suspension” AND experiment*
26) “school suspension” AND evaluation
27) “school suspension” AND random*
28) “school suspension” AND intervention,
29) “school suspension” AND effective*,
30) “school suspension” AND efficacy,
31) “school suspension” AND quasi,
32) “school suspension” AND impact,
33) “school suspension” AND RCT,
34) “school suspension” AND school management
35) “school suspension” AND classroom management
36) “school suspension” AND school support project*
37) “school suspension” AND skills training
38) “school suspension” AND disciplinary methods
39) “school suspension” AND token economy
40) “school suspension” AND program*
41) “school suspension” AND *intervention*
42) “school suspension” AND strateg*
43) “school suspension” AND schoolchildren
44) “school suspension” AND *children*
45) “school suspension” AND school‐age*
46) “school suspension” AND adolescent*
47) “school suspension” AND pupil*
48) “school suspension” AND student*
49) “school expulsion” AND experiment*
50) “school expulsion” AND evaluation
51) “school expulsion” AND random*
52) “school expulsion” AND intervention,
53) “school expulsion” AND effective*,
54) “school expulsion” AND efficacy,
55) “school expulsion” AND quasi,
56) “school expulsion” AND impact,
57) “school expulsion” AND RCT,
58) “school expulsion” AND school management
59) “school expulsion” AND classroom management
60) “school expulsion” AND school support project*
61) “school expulsion” AND skills training
62) “school expulsion” AND disciplinary methods
63) “school expulsion” AND token economy
64) “school expulsion” AND program*
65) “school expulsion” AND *intervention*
66) “school expulsion” AND strateg*
67) “school expulsion” AND schoolchildren
68) “school expulsion” AND *children*
69) “school expulsion” AND school‐age*
70) “school expulsion” AND adolescent*
71) “school expulsion” AND pupil*
72) “school expulsion” AND student*

**SciElo**

Date: 06/10/ 2015

Output of the searches: 32

Saved hits: 0

Final searches string:

1) “school exclusion evaluation”
2) “school exclusion experiment”
3) “school suspension evaluation”
4) “school suspension experiment”
5) “school expulsion evaluation”
6) “school expulsion experiment”
7) suspension escolar
8) suspension escolar evaluación
9) suspension escolar experiment
10) expulsion escolar
11) expulsion escolar evaluación
12) expulsion escolar experiment
13) expulsion escuela

**Sciencegov**

Date: From 05/10/ 2015 to 06/10/2015

Output of the searches: 319

Saved hits: 61

Final searches string:

1) “school suspension” AND evaluation
2) “school suspension” AND experiment*
3) “school exclusion” AND evaluation
4) “school exclusion” AND experiment*
5) “school expulsion” AND evaluation
6) “school expulsion” AND experiment*

**The Campbell Collaboration Social, Psychological, Educational and Criminological Trials Register (C2‐SPECTR)**

Date: 16/10/2015

Output of the searches: 1

Saved hits: 1

Final searches string:

1) school exclusion
2) school suspension
3) school suspended
4) school expelled
5) school expulsion
6) school stand down

**Trials Journal**

Date: 06/10/ 2015

Output of the searches: 56

Saved hits: 1

Final searches string:

1) school exclusion
2) school suspension
3) school suspended
4) school expelled
5) school expulsion
6) school stand down

**WHO‐ International Clinical Trials Registry Platform (ICTRP)**

Date: 30/09/2015

Output of the searches: 3

Saved hits: 3

Final searches string:

1) “school exclusion” (other outcome terms searched with no results)

## Footnotes

1 In this document school exclusion and school suspension are used synonymously.

2 Odds ratios are presented without confidence intervals or p‐values as in the source.

3 The strong link between school exclusion and subsequent school drop‐out/entry into the juvenile justice system has been termed the school‐to‐prison pipeline (Snyder & Sickmund, 2006; Fenning et al., 2012;Chin et al., 2012 Christle et al., 2005; Nicholson‐ Crotty et al., 2009). It describes the escalating punitive consequences of harsh discipline in school and its exclusionary practices.

4 Studies reporting a large difference between the treatment and control group at pre‐test were said to be excluded as they would not help in distinguishing intervention effects from other effects. The protocol also stated that quasi‐experimental studies based on one group pre‐test and post‐test designs or one group post‐test‐only designs should be excluded from the review.

5 The assumption of equal sample size seems to be a good approximation for the calculation of effect sizes. Hedges, (2007) asserts that effect size calculation based on equal and unequal cluster sizes are not substantially different.

6 The presented flow chart has been adapted from Moher, Liberati, Tetzlaff, & Altman, (2009)

7 At the beginning of the evaluation we selected two studies and coded them as a pilot assessment to ensure we understood the criteria consistently. This exercise helped to test the instrument and also to adjust some criteria. Following the complete coding of all studies, the coders met in late June 2016 to discuss and moderate their ratings.

8 How many variables would we expect to be out of balance by chance in an RCT? This figure is usually obtained by the following calculation (k*0.05) where k is the number of variables, so 20*0.05 = 1.

9 Many of our included studies were non‐published PhD thesis (i.e., 18 out of 37). As a result, it was impossible to find pre‐registration publications for these studies. We observed that most of the theses reported small or null effects (i.e., Arter, 2005; Bragdon, 2010; Brett, 1993; Burcham, 2002; Crowder, 2001; Harding, 2011; Peck, 2006). Since null and small effects were reported, we assumed low risk of bias regarding selective outcome reporting.

10 Substance misuse was not included in the calculation.

11 The original effect sizes are presented in Fig. 2.1, page 63. They are reported in OR; we have converted those values to make it more comparable with the metric we use in the present review.

12 To calculate the impact of exclusion on dropout rates, the study used longitudinal data collected via surveys and standardised tests to observe the percentage of students who graduated on time, two years after the expected date of graduation, and four years after. The independent variable was exclusion and the study involved independent controls such as demographic variables, family variables and school performance variables. All in all, the study showed that in the U.S., only 71 percent of tenth graders who received a suspension graduated from high school, compared to 94 percent of tenth graders who did not receive a suspension. In other words, being suspended [was] associated with a 23 percentage‐point decrease in the likelihood of graduating (Rumberger & Losen, 2016; p.12).

13 Based on Lipsey & Wilson (2001)
